# A Nomenclator of *Croton* (Euphorbiaceae) in Madagascar, the Comoros Archipelago, and the Mascarene Islands

**DOI:** 10.3897/phytokeys.90.20586

**Published:** 2017-11-15

**Authors:** Paul E. Berry, Kent Kainulainen, Benjamin W. van Ee

**Affiliations:** 1 Herbarium, Department of Ecology and Evolutionary Biology, University of Michigan, 3600 Varsity Drive, Ann Arbor, Michigan 48108, U.S.A.; 2 Department of Biology, Universidad de Puerto Rico, Recinto Universitario de Mayagüez, Mayagüez, PR 00680, Puerto Rico, U.S.A.

**Keywords:** Euphorbiaceae, *Croton*, Madagascar, Comoros, Mascarenes, nomenclator, synonymy

## Abstract

All published names of *Croton* from Madagascar, the Comoros, and the Mascarenes are treated here. We indicate which names are currently accepted (123 native species and 1 introduced), which ones we consider to be heterotypic synonyms (188), which ones are doubtful (25), and which ones should be excluded (5). We newly designate lectotypes for 108 names, and epitypes for *C. anisatus* Baill., *C. nobilis* Baill., and *C. submetallicus* Baill. A total of 133 names are newly treated as synonyms. One new combination is made, *Croton basaltorum* (Leandri) P.E.Berry for C. antanosiensis var. basaltorum Leandri, and one new name is proposed, *Croton toliarensis* B.W.vanEe & Kainul. for C. tranomarensis var. rosmarinifolius Radcl.-Sm.

## Introduction

This work is part of an effort to lay the taxonomic foundation for a thorough phylogenetic-based revision of *Croton* (Euphorbiaceae) in the Western Indian Ocean Region (WIOR). Madagascar alone harbors around one third of the roughly 450 species recognized to occur in the Old World (Van Ee et al. 2011). There are also several species known to occur on the Mascarene Islands (Mauritius and the French Department of Réunion), as well as on the Comoros Islands (Union of the Comoros and the French Department of Mayotte). There are no known species of *Croton* in the Seychelles or in any of the smaller, “Scattered Islands” of the WIOR. Because of the relative proximity of these islands to Madagascar, and the phylogenetic findings of Haber et al. (2017) that recovered all sampled *Croton* species from the Comoros and Mascarenes within a Malagasy clade, all described *Croton* taxa from this region are treated together here. The main purpose of this paper is to provide a comprehensive nomenclatural index of the published names of *Croton* from the Western Indian Ocean Region and to indicate their current taxonomic and nomenclatural status, based on our ongoing taxonomic and phylogenetic work on the genus.

Until 2016, taxa of *Croton* native to the Comoros, Madagascar, and the Mascarenes were published by just eight botanists in the following 27 books or journal articles: Lamarck (1786); Geiseler (1807); Baillon (1861, 1890, 1891a); Müller (1864, 1865, 1866); Baker (1882, 1883, 1887); De Candolle (1901); Leandri (1931, 1935, 1939, 1948, 1957, 1970a, 1970b, 1970c, 1972a, 1972b, 1973a, 1973b, 1974, 1976); and Croizat (1944). In 2016, a large number of new *Croton* taxa from the WIOR were described. Ten new species of *Croton* from Madagascar were published by Kainulainen et al. (2016) and Berry et al. (2016a, 2016b). Then, taking advantage of an overly literal interpretation of Article 29.1 of the International Code of Nomenclature for algae, fungi, and plants (McNeill et al. 2012), Martin Cheek of the Royal Botanic Gardens, Kew, submitted three “preprint” copies of a long-dormant manuscript on Malagasy *Croton* by the late botanist Alan Radcliffe-Smith to the libraries of Kew, Wisley, and the Natural History Museum in London toward the end of December 2016. By early January 2017, most of the names from that manuscript had been posted on the International Plant Names Index website (www.ipni.org), with an effective publication date of 23 December 2016 (Radcliffe-Smith 2016). This manuscript added 150 newly described names of *Croton* from Madagascar and the Comoros Archipelago, consisting of 26 new species, three new subspecies, and 121 new varieties. All of these recent names have been evaluated here, and our assessment of their current taxonomic status is presented in Tables 1 and 2 and in the “Incertae Sedis” section at the end of the nomenclator.

## Materials and methods

The main herbaria consulted for the WIOR *Croton* types were G, K, MICH, MAO, MO, P, TAN, and TEF. Scanned images of types from these and other herbaria available on JSTOR Global Plants (http://plants.jstor.org/) were also consulted. All original literature sources were also reviewed.

Specimens are considered holotypes when a single gathering in a particular herbarium is cited in the protologue, and there is only one specimen of that gathering housed there, or if there was a single gathering with no herbarium mentioned in the protologue, or else a single specimen exists in the herbarium where the author was based (ICN Art. 9.1, McNeill et al. 2012; McNeill 2014). In some publications, such as Leandri (1970b, 1973a, 1974) and Radcliffe-Smith (2016), the protologue indicates a holotype, but there is more than one sheet of that collection at the herbarium cited. In such cases, we rely on the notations on the sheets to determine whether a holotype can be identified; if not, a lectotype is designated. When two or more syntypes were cited in the protologue, a lectotype is designated, using our informed assessment of the most appropriate specimen. In a few cases, an epitype is also newly designated, when the condition of the holotype or lectotype is so poor as to make the identity of the species unclear.

Since the name *Croton* is derived from the Greek κροτων (tick), which is masculine, this is the proper gender for *Croton.* However, it was sometimes treated as neuter or as feminine by Baillon and Leandri, and this now requires that the epithets with those endings be orthographically corrected to the masculine ending. In such cases, the original spelling of an epithet is given, and users should be aware of the need to search databases using both the original spelling and subsequent, corrected spellings.

Within the taxon citations, if a lectotype is designated among the extant syntypes, the remaining syntypes are also cited. Type locality information is taken directly from the type specimen labels, even if it does not entirely agree with the location given in the protologue. If we add information not present on the specimen labels, such as the province name, or a more modern or accepted spelling of a place name, that information is placed within brackets. Barcode numbers of type specimens are cited when available, but as of late October 2017, the type specimens cited by Radcliffe-Smith (2016) have not yet been assigned barcodes and do not yet appear on the JSTOR Global Plants website. Whenever we newly treat a taxon as a synonym, we include the annotation “**syn. nov.**” at the end of the entry. Lastly, under the “Habit and Distribution” section of each entry from Madagascar, we list the general distribution of the species on the island and include the former province names where they occur in parentheses.

## Results and discussion

In the present treatment we recognize 114 accepted species native to Madagascar. All of these species are endemic to the island, with the exception of *C. adenophorus* Baill., which also occurs on Mayotte in the Comoros. Besides this species, there are four species endemic to the Comoros (*C. bifurcatus* Baill., *C. emeliae* Baill., *C. humblotii* Baill., and *C. mayottae* P.E.Berry & Kainul.). Another four species are endemic to Mauritius (*C. fothergillifolius* Baill., *C. grangerioides* Bojer ex Baill., *C. tiliifolius* Lam., and *C. vaughanii* Croizat), and a fifth (*C. mauritianus* Lam.) is endemic to Réunion. *Croton bonplandianus* Baill., a native of South America, is the only documented non-native, naturalized species of *Croton* in the WIOR, having previously been reported from the Mascarenes (Mauritius, Réunion, and Rodrigues; Croizat 1944, Coode 1982). We also report here the occurrence of *C. bonplandianus* from Mayotte. Other species that are likely to become naturalized weeds, but have not been reported from the region, include *Astraea lobata* L. (formerly *Croton lobatus* (L.) Klotzsch), *C. hirtus* L’Hér., and *C. glandulosus* L. Several non-native species have been cultivated on the islands in the past, presumably for their medicinal or shade properties, such as *C. aromaticus* L., *C. haumanianus* J.Léonard, *C. laccifer* L., and *C. tiglium* L., but there is no evidence that they have persisted.

For the 114 species that are native to Madagascar, only six are truly widespread throughout the island, occurring in all six former provinces, namely *C. catatii* Baill., *C. goudotii* Baill., *C. macrobuxus* Baill., *C. mongue* Baill., *C. myriaster* Baker, and *C. stanneus* Baill. Three others occur in five provinces (*C. chrysodaphne* Baill., *C. hypochalibaeus* Baill., and *C. nitidulus* Baker), five in four provinces, six in three provinces, and 29 in two provinces. Sixty-five species are known from just a single province. Of the six provinces, Toliara is the richest in *Croton* species with 53, followed by Antsiranana with 43, Toamasina with 39, Mahajanga with 35, Fianarantsoa with 27, and Antananarivo with 15. While we realize that these six administrative provinces of Madagascar have been superseded by a system of 22 administrative regions that represent subdivisions of the provinces, we did not attempt to further refine the distribution of species, although this will be a promising avenue to pursue in the future for conservation planning efforts. See Fig. 2 for a summary of these figures.

Given the small size of the islands in the Mascarenes and the Comoros Archipelago, and the small area of existing natural habitats, we believe that it is fairly unlikely that any additional *Croton* taxa will be discovered there. On the other hand, Madagascar harbors a large number of undescribed species, which are currently under study by the authors and awaiting formal description. We anticipate the description of at least 20 more new species from Madagascar based on the material we now have on hand. Many of these come from recently explored areas in the eastern part of the island, but also from relatively well-explored areas in the north and south but belonging to difficult species complexes, such as the coppery, lepidote-leaved species.

The sometimes extensive synonymy presented in this nomenclator (viz., *C. adenophorus, C. catatii*, *C. chapelieri*, or *C. stanneus*) suggests that many species have been poorly understood in the past, sometimes known only from sparse descriptions or poor type material, and numerous taxa are based on a single or just a few specimens. For instance, from Baillon (1861) to Leandri (1939) to Radcliffe-Smith (2016), all three authors recognized *Croton chapelieri* as an accepted species, but known only from the type, which was of unspecified provenance. The re-evaluation of this species by Kainulainen et al. (2017a), and again in this paper, reveals that *C. chapelieri* is in fact a widespread species along the eastern littoral zone, and we now consider that it has eight heterotypic synonyms. We should point out that without first-hand knowledge of the species in the field, it may have been impossible to sort out the variability of this species and recognize its restriction to a fairly narrow ecological and elevational zone in sandy coastal habitats. Among earlier workers on the genus in the region, the only one who had any first-hand knowledge of Madagascar was Jacques Leandri. In contrast, we have made four collecting trips to Madagascar dedicated to studying *Croton*, covering much of the geography of the island, although there are still numerous areas we have not been able to visit. Especially for deciduous species, it is important to collect specimens at different times of the year, since some species flower when leafless and produce their leaves during the rainy season. Ultimately, the greatest progress in understanding the diversity of a large, complex genus like *Croton* on Madagascar will be achieved by local botanists who are able to study populations in the field, revisit sites at different phenological stages, and assess their local and regional variability.

**Figure 1.**
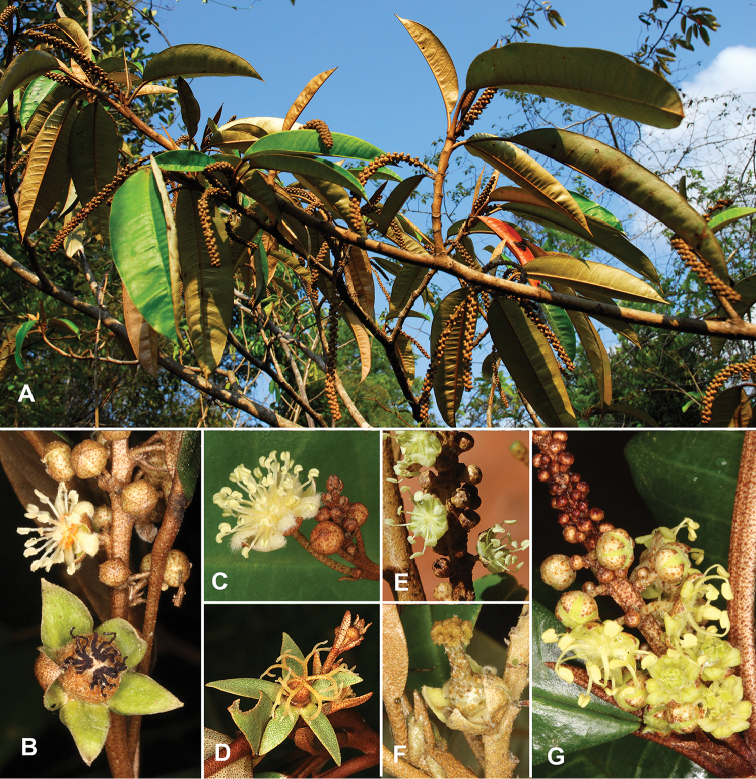
Diversity of flowers in coppery-lepidote tree species of *Croton* from Madagascar that are vegetatively very similar. **A** *Croton argyrodaphne*, with leaves that are similar to those of several other species **B** Part of an inflorescence of *Croton nobilis* showing pistillate flower (below) with thick, reduplicate sepals and no petals, and staminate flower (above) with an intermediate number of stamens (ca. 18) **C** Staminate flower of *C. chrysodaphne*, with numerous (ca. 40) stamens and the unusual feature of ten (vs. normally five) petals **D** Pistillate flower of *C. chrysodaphne*, with patent, slender bifurcating styles and no petals **E** Staminate flowers of *C. argyrodaphne*, with only 11 stamens **F** Pistillate flower of *C. argyrodaphne*, with a stylar column topped by tightly bunched, short stigmas and also with recurved petals between the sepals (typically the pistillate flowers of this species are apetalous) **G** Base of an inflorescence of *C. multicostatus* showing three open pistillate flowers at the base (with well-developed, ligulate petals) and several open staminate flowers showing a low stamen number of 10 or 11. Photos by P. Berry.

Another instructive example of a previously misunderstood species is *Croton chrysodaphne* Baill., a tree species that Baillon (1861) described based on three syntypes from Madagascar. Baillon's concept of this species was followed by both Leandri (1939) and Radcliffe-Smith (2016), but Berry et al. (2011) determined that the three syntypes in the protologue actually represent three different species, and while one of the others had a valid name (*C. argyrodaphne*), it took several more years to determine with certainty that the third one was a new species, *C. cupreolepis* P.E.Berry, B.W.van Ee, & Kainul. (Berry et al. 2016b). As a further example, Berry and Van Ee (2011) also deciphered the history of *C. multicostatus* Müll.Arg., a Malagasy species which had erroneously been attributed to Hispaniola in the Caribbean, and included as synonyms two species names from Madagascar, *C. vernicosus* Baker and *C. sclerodorus* Baill. Figure 1A shows an example of foliage that is similar in all these coppery-lepidote tree species, while Fig. 1B–G show major differences in flowers of four species in this assemblage in terms of sepal shape, stigma morphology, and presence or absence of petals in the pistillate flowers, as well as differences in the number of stamens in the staminate flowers.

**Figure 2.**
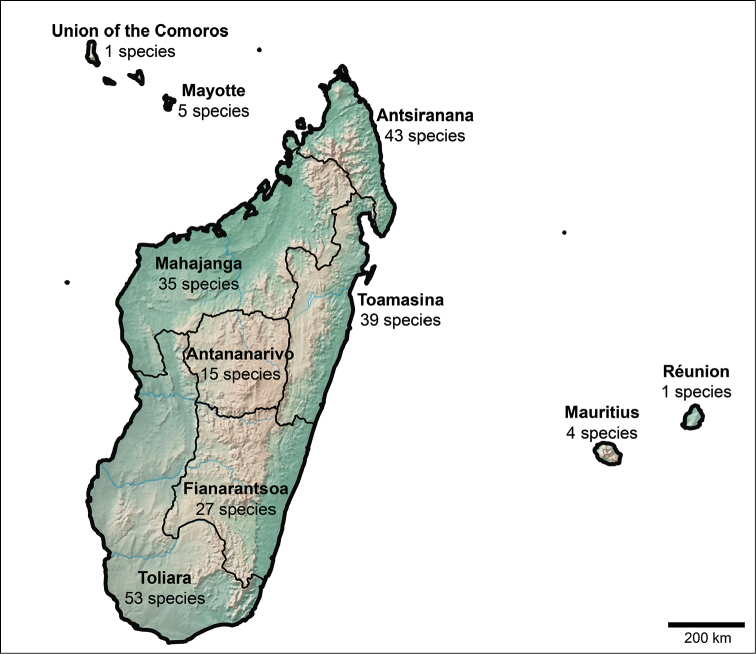
Distribution of the number of native species of *Croton* in the Western Indian Ocean Region. There are 123 native species overall in the region, with 114 native to Madagascar; one of them (*C. adenophorus*) is shared with Mayotte, and the single species in the Union of the Comoros (*C. humblotii*) also occurs on Mayotte. The species on Mauritius and Réunion only occur there. The map of Madagascar shows how many species occur in each of the six former provinces (there are varying levels of overlap between provinces; see text for details).

The recent, hastily published work of Radcliffe-Smith (2016) appears to us to illustrate the problem of a lack of field knowledge in the region and the consequent lack of understanding of the variability in many *Croton* species. In some cases, Radcliffe-Smith picked up on important differences in specimens, but he was very inconsistent in how he treated them. Out of the 150 new taxa that he described, 89 were known only from the type collection, and another 29 were known from just two collections. Such a narrow circumscription tends to recognize every minor variation as a separate taxon. On the other hand, in some cases very distinctive new species were treated as varieties of previously known species. For example, *Croton bemaranus* Leandri is a small shrub, and what Radcliffe-Smith (2016) described as C. bemaranus var. pseudolepidotus Radcl.-Sm. is a very distinctive large tree that was rediscovered in the field and described by Berry et al. (2016a) a few months earlier as *C. aleuritoides* P.E.Berry. Our general stance for recognizing varieties for Malagasy crotons is that we do not yet have a sufficient level of understanding of the majority of the species to make meaningful subspecific designations. This may come in time, but the fact that more of Radcliffe-Smith's (2016) published varieties fell into synonymy under different species than the ones to which they were assigned (see below) is an indication that we are not yet at the stage where widespread designations of infraspecific taxa are advisable.

The overall disposition of the taxa described by Radcliffe-Smith (2016) is given in Tables 1 and 2. Of the 26 species and three subspecies that he described, only four are maintained here as accepted taxa (Table 1), and all others are considered to be synonyms of previously described species. Of the 121 new varieties described, we synonymize 97, more often than not under different species than the ones to which they were assigned (Table 2). Of the remaining 24 varieties described, 22 are included in the ‘Incertae Sedis’ section and two are not validly published (see Names Not Validly Published). Based on this outcome, we contend that the manner in which the new taxa in Radcliffe-Smith (2016) were brought to effective and valid publication, namely by the hand-delivering of three copies of a minimally reviewed and edited manuscript to several London libraries while simultaneously having the taxa entered into IPNI at Kew, avoided a badly needed, more rigorous review of the manuscript and led to the useless creation of a great many names, in contravention of Preamble paragraph 1 of the ICN (McNeill et al. 2012). In our view, the Radcliffe-Smith (2016) publication is not at all an accurate reflection of our current knowledge of Malagasy *Croton* taxonomy, and it should not be consulted as such. Rather, with this paper, previous ones we have published (Berry and Van Ee 2011; Berry and Kainulainen 2017; Berry et al. 2011, 2016a, 2016b; Kainulainen et al. 2016, 2017a, 2017b), our initial molecular results (Van Ee et al. 2011, 2015; Haber et al. 2017) and with more complete molecular studies forthcoming shortly, as well as additional taxonomic novelties and revisions in preparation, we are generating a significantly different and better substantiated vision of the rich diversity of *Croton* in the Western Indian Ocean Region.

**Table 1.** New species and subspecies of Malagasy *Croton* described by Radcliffe-Smith (2016) and their treatment in this paper. Radcliffe-Smith described 26 new species and 3 new subspecies, besides the 121 varieties treated in Table 2. The four Radcliffe-Smith names in bold below are the only ones maintained here as accepted taxa.

Radcliffe-Smith (2016) name	As treated in this paper
*C. adenophoroides* Radcl.-Sm.	*C. loucoubensis* Baill.
*C. alaotrensis* Radcl.-Sm.	C. *droguetioides* Kainul. & Radcl.-Sm.
*C. alceicornu* Radcl.-Sm.	*C. hypochalibaeus* Baill.
***C. alchorneifolius*** Radcl.-Sm.	***C. alchorneifolius*** Radcl.-Sm.
C. bracteatus subsp. manongarivensis Radcl.-Sm.	*C. nitidulus* Baill.
C. bracteatus subsp. populifolius Radcl.-Sm.	*C. danguyanus* Leandri
*C. commiphoroides* Radcl.-Sm.	*C. indrisilvae* Kainul., B.W.vanEe & P.E.Berry
*C. daphniphylloides* Radcl.-Sm.	*C. chapelieri* Baill.
*C. daphniphyllus* Radcl.-Sm.	*C. chapelieri* Baill.
*C. dasygyne* Radcl.-Sm.	*C. radiatus* P.E.Berry & Kainul.
*C. delicatulus* Radcl.-Sm.	*C. menabeensis* Leandri
*C. domohineifolius* Radcl.-Sm.	*C. chapelieri* Baill.
*C. droguetioides* Radcl.-Sm.	*C. droguetioides* Kainul. & Radcl.-Sm.
*C. echinatus* Radcl.-Sm.	*C. dissimilis* Baill.
***C. gracilior*** Radcl.-Sm.	***C. gracilior*** Radcl.-Sm.
*C. hirsutissimus* Radcl.-Sm.	*C. nudatus* Baill.
*C. lepidotoides* Radcl.-Sm.	*C. ferricretus* Kainul., B.W.vanEe & P.E.Berry
***C. minimimarginiglandulosus*** Radcl.-Sm.	***C. minimimarginiglandulosus*** Radcl.-Sm.
C. mocquerysii subsp. meridionalis Radcl.-Sm.	*C. thouarsianus* Baill.
*C. neoholstiifolius* Radcl.-Sm.	*C. menabeensis* Leandri
*C. oligostemon* Radcl.-Sm.	*C. hypochalibaeus* Baill.
*C. parietarioides* Radcl.-Sm.	*C. droguetioides* Kainul. & Radcl.-Sm.
*C. parvifructus* Radcl.-Sm.	*C. stanneus* Baill.
*C. remotiflorus* Radcl.-Sm.	*C. trichotomus* Geisel.
*C. rhododendroides* Radcl.-Sm.	*C. chapelieri* Baill.
*C. scorpistogyne* Radcl.-Sm.	*C. heteranthus* Aug.DC.
*C. submetallicoides* Radcl.-Sm.	*C. chyrsodaphne* Baill.
***C. ustulatus*** Radcl.-Sm.	***C. ustulatus*** Radcl.-Sm.
*C. vohemarensis* Radcl.-Sm.	*C. argyrodaphne* Baill.

**Table 2.** New varieties of Malagasy *Croton* described by Radcliffe-Smith (2016) and their treatment in this paper. Radcliffe-Smith described 121 new varieties besides the 26 new species and 3 new subspecies listed in Table 1. Of the 121 new varietal names, 97 are reduced to synonymy (45 under the nominal species name and 52 under a different species), 22 are considered as Incertae Sedis, and two are invalid.

**Radcliffe-Smith (2016) name**	**As treated in this paper**
*C. adabolavensis* var. *hippophaëoïdes*	*C. adabolavensis* Leandri
C. adabolavensis var. microlepidotus	*C. adabolavensis* Leandri
C. adabolavensis var. ovalifolius	*C. adabolavensis* Leandri
C. adabolavensis var. stellatipilus	*C. adabolavensis* Leandri
C. alaotrensis var. integrifolius	*C. dissimilis* Baill.
C. ambovombensis var. lepidotus	nom. inval. (Excluded Taxa)
C. anisatus var. hirsutus	*C. hovarum* Leandri
C. ankarensis var. ankarafantsikae	Incertae Sedis
C. anosiravensis var. pilosus	*C. anosiravensis* Leandri
C. antanosiensis var. ambohibyi	*C. trichotomus* Geisel.
C. antanosiensis var. fianarantsoae	*C. hypochalibaeus* Baill.
C. antanosiensis var. pubescens	*C. greveanus* Baill.
C. bastardii var. bongolavae	Incertae Sedis
C. bastardii var. meridionalis	*C. muricatus* Vahl
C. bathianus var. ambatondrazakae	*C. scoriarum* Leandri
C. bathianus var. ihosianus	*C. ihosianus* Leandri
C. bathianus var. toliarae	*C. crocodilorum* Baill.
C. bemaranus var. parvistipulatus	Incertae Sedis
C. bemaranus var. pseudolepidotus	*C. aleuritoides* P.E.Berry
C. betiokensis var. haplostylis	Incertae Sedis
C. bifurcatus var. humblotii	*C. bifurcatus* Baill.
C. boinensis var. parcelepidotus	*C. greveanus* Baill.
C. boinensis var. tomentosus	*C. catatii* Baill.
C. boivinianus var. brevifolius	*C. nudatus* Baill.
C. cassinoides var. alaotrensis	*C. hovarum* Leandri
C. catatii var. schizoplepis	Incertae Sedis
C. catatii var. setosus	*C. catatii* Baill.
C. catatii var. tricholepis	*C. catatii* Baill.
C. chapelieri var. longepetiolata	*C. submetallicus* Baill.
C. chrysodaphne var. meridionalis	*C. cupreolepis* P.E.Berry, B.W.vanEe, & Kainul.
C. crocodilorum var. meridionalis	*C. crocodilorum* Leandri
C. crocodilorum var. platyaster	*C. stanneus* Baill.
C. daphniphylloides var. hirsutus	*C. nitidulus* Baker
C. daphniphylloides var. stellatipilus	Incertae Sedis
C. daphniphylloides var. triplinervius	Incertae Sedis
C. daphniphyllum var. hirsutus	*C. fianarantsoae* Leandri
C. decaryi var. subglaber	*C. muricatus* Vahl
C. elaeagni var. antsirananae	*C. elaeagni* Baill.
C. elaeagni var. argyrocarpos	Incertae Sedis
C. elaeagni var. brevipedicellatus	Incertae Sedis
C. elaeagni var. chrysocarpos	*C. catatii* Baill.
C. fianarantsoae var. ambremontanus	*C. minimimarginiglandulosus* Radcl.-Sm.
C. fianarantsoae var. coursii	*C. fianarantsoae* Leandri
C. fianarantsoae var. masoalae	*C. glomeratus* Aug.DC.
C. fianarantsoae var. microphyllus	*C. fianarantsoae* Leandri
C. fianarantsoae var. obovalifolius	*C. submetallicus* Baill.
C. fianarantsoae var. petiolaris	Incertae Sedis
C. fianarantsoae var. ranomafanae	*C. fianarantsoae* Leandri
C. fianarantsoae var. tandrokensis	*C. fianarantsoae* Leandri
C. geayi var. paucisquamatus	*C. geayi* Leandri
C. geayi var. pubescens	*C. geayi* Leandri
C. goudotii var. tsaratananae	*C. goudotii* Baill.
C. greveanus var. ambositrensis	*C. hovarum* Leandri
C. greveanus var. micraster	*C. catatii* Baill.
C. hovarum var. hirsutifructus	Incertae Sedis
C. hovarum var. lepidotus	*C. hovarum* Leandri
C. hovarum var. subglaber	*C. hovarum* Leandri
C. incisus var. minor	*C. incisus* Baill.
C. isomonensis var. microcarpus	*C. trichotomus* Geisel.
C. ivohibensis var. aesculops	*C. minimimarginiglandulosus* Radcl.-Sm.
C. ivohibensis var. alaotrensis	*C. humbertii* Leandri
*C. ivohibensis var. ankaranaënsis*	*C. minimimarginiglandulosus* Radcl.-Sm.
C. ivohibensis var. furfuraceus	*C. heteranthus* Aug.DC
C. ivohibensis var. integrifolius	Incertae Sedis
C. ivohibensis var. lepidotus	*C. hovarum* Leandri
C. ivohibensis var. macrocalyx	Incertae Sedis
C. ivohibensis var. polygynus	Incertae Sedis
C. ivohibensis var. puncticulatus	*C. bracteatus* Lam.
C. ivohibensis var. verticillatus	Incertae Sedis
C. kimosorum var. pubescens	*C. kimosorum* Leandri
C. leandri var. pubescens	*C. toliarensis* B.W.vanEe & Kainul.
C. lichenisilvae var. oligostemon	*C. lichenisilvae* Leandri
C. macrobuxus var. dolichobotrys	*C. macrobuxus* Baill.
C. macrobuxus var. glandulifer	*C. macrobuxus* Baill.
C. macrobuxus var. polygynus	*C. chapelieri* Baill.
C. macrobuxus var. subfoliaceus	*C. macrobuxus* Baill.
C. macrobuxus var. substrigosus	*C. macrobuxus* Baill.
C. manampetsae var. angustifolius	*C. manampetsae* Leandri
C. manampetsae var. chaetogyne	*C. manampetsae* Leandri
C. manampetsae var. lepidotus	Incertae Sedis
C. mavoravina var. concinnus	*C. boiteaui* Leandri
C. mavoravina var. gracilis	*C. mavoravina* Leandri
C. mavoravina var. gymnolepis	*C. mavoravina* Leandri
C. mavoravina var. imanombensis	*C. mavoravina* Leandri
C. mavoravina var. rotundifolius	*C. mavoravina* Leandri
C. mavoravina var. thysanolepis	*C. crossolepis* P.E.Berry & Kainul.
C. menabeensis var. furfuraceus	*C. nudatus* Baill.
C. menarandrae var. pubescens	*C. menarandrae* Leandri
C. meridionalis var. latifolius	Incertae Sedis
C. meridionalis var. pseudolepidotus	*C. meridionalis* Leandri
C. meridionalis var. stipularis	Incertae Sedis
C. miarensis var. monadenius	*C. miarensis* Leandri
C. mongue var. borealis	*C. mongue* Baill.
C. nitidulus var. acuminatus	*C. submetallicus* Baill.
C. nitidulus var. angustiglans	*C. nitidulus* Baker
C. nitidulus var. bekolosiensis	*C. nitidulus* Baker
C. nitidulus var. cinereus	*C. submetallicus* Baill.
C. nitidulus var. eglandulosus	Incertae Sedis
C. nitidulus var. fuscicrameus	*C. nitidulus* Baker
C. nitidulus var. hypopoliotes	*C. submetallicus* Baill.
C. nitidulus var. macrophyllus	*C. submetallicus* Baill.
C. nitidulus var. microphyllus	*C. macrobuxus* Baill.
C. nitidulus var. pubescens	*C. macrobuxus* Baill.
C. oreades var. borealis	*C. mongue* Baill.
C. oreades var. craspedadenius	*C. mongue* Baill.
C. oreades var. periphoradenius	*C. mongue* Baill.
C. peltieri var. hazofotsiensis	*C. miarensis* Leandri
C. regeneratrix var. mayottensis	*C. mayottae* P.E.Berry & Kainul.
C. regeneratrix var. ranomafanae	*C. myriaster* Baker
C. rubricapitirupis var. macrophyllus	Incertae Sedis
C. stanneus var. hirsutus	*C. stanneus* Baill.
C. subaemulans var. tsingyensis	*C. bemaranus* Leandri
C. submetallicus var. tomentosus	*C. submetallicus* Baill.
C. thuarsianus var. angustifolius	Incertae Sedis
C. thuarsianus var. longifolius	Incertae Sedis
C. thuarsianus var. macrocalyx	*C. minimimarginiglandulosus* Radcl.-Sm.
C. thuarsianus var. robustior	*C. thouarsianus* Baill.
C. tranomarensis var. rosmariniifolius	*C. toliarensis* B.W.vanEe & Kainul.
C. tsiampiensis var. ankaranensis	*C. tsiampiensis* Leandri
C. tsiampiensis var. macrophyllus	*C. tsiampiensis* Leandri
C. tsiampiensis var. microphyllus	*C. tsiampiensis* Leandri

### Nomenclator of Malagasy, Comoros, and Mascarene *Croton*

#### 1. Croton adabolavensis

Taxon classificationPlantaeMalpighialesEuphorbiaceae

Leandri, Ann. Mus. Colon. Marseille, sér. 5, 7(1): 34. 1939

Croton adabolavensis var. *hippophaëoïdes* Radcl.-Sm., Gen. Croton Madag. Comoro 92. 2016, **syn. nov.** Type. Madagascar. Prov. Toliara: Cap Ste. Marie Reserve, SW of Tsiombe, 25°35'S, 45°09'E, 27 Dec 1988, *P.B. Phillipson 3008* (holotype: K!; isotypes: MO!, P [P00433074]!).
Croton adabolavensis var. microlepidotus Radcl.-Sm., Gen. Croton Madag. Comoro 92. 2016, **syn. nov.** Type. Madagascar. Prov. Toliara: Réserve Naturelle Intégrale d’Andohahela, along border road, 25°00'S, 46°40'E, 19 Oct 1990, *A. Randrianasolo*, *D. Faber-Langendoen*, *N. Dumetz* & *R. Rabevohitra 174* (holotype: K!; isotypes: MO!, P [P00433039]!).
Croton adabolavensis var. ovalifolius Radcl.-Sm., Gen. Croton Madag. Comoro 92. 2016, **syn. nov.** Type. Madagascar. Prov. Toliara: Beza Mahafaly Reserve, near Betioky, ridge E of Sakamena River, valley of Analafahy River, 23°58'S, 44°39'E, 28 Nov 1987, *P.B. Phillipson 2621* (holotype: K!; isotype: MO!, P [P00133395]!).
Croton adabolavensis var. stellatipilus Radcl.-Sm., Gen. Croton Madag. Comoro 93. 2016, **syn. nov.** Type. Madagascar. Prov. Toliara: 27 km from Tulear (Toliara), 23°21'S, 43°51'E, 30 Dec 1987, *P.B. Phillipson 2759* (holotype: K!; isotype: MO!, P [P00133397]!).

##### Type.

Madagascar. Prov. Toliara: Anosy Region, vallée moyenne du Mandrare, près d’Anadabolava, forêt sèche, 200–250 m, Dec 1933, *H. Humbert 12498* (lectotype, designated here: P [P00248927]!; isolectotype: P [P00312384]!).

##### Habit and distribution.

Shrubs; southeastern Madagascar (Toliara).

##### Notes.

Leandri (1939) named the species after the type locality, Anadabolava, but he may have inadvertently or even purposely omitted the first two letters of the name. The different varieties described by Radcliffe-Smith (2016) all appear to be just minor variants within the normal variability of this species that do not merit taxonomic status, but more intensive work is needed on this and the other small-leaved species from Toliara Province.

#### 2. Croton adenophorus

Taxon classificationPlantaeMalpighialesEuphorbiaceae

Baill., Adansonia 1: 153. 1861, as ‘adenophorum’

Croton payerianus Baill., Adansonia 1: 154. 1861, as ‘*payerianum*’. Type. Madagascar. Prov. Antsiranana: Diana Region, Nossibé, 1849, *L.H. Boivin 2187* (lectotype, designated here: P [P00389508]!; isolectotypes: G-DC [G00311984]!, G [G00446358]!, G [G00446359]!, P [P00389509]!, P [P00404480]!, P [P00404481]!, P [P00123684]!).
Croton tulasnei Baill., Adansonia 1: 156. 1861. Type. Mayotte [French Overseas Department]: Bouzi [Ilot M’Bouzi], 1850, *L.H. Boivin s.n.* (lectotype, designated by Kainulainen et al. 2017b, pg. 382: P [P00133305]!; isolectotypes: P [P00133306]!, [P00466148]).
Croton subaemulans Baill., Bull. Mens. Soc. Linn. Paris 2: 850. 1890. Type. Madagascar. sin. loc., *R. Baron 5795* (holotype: K [K000422590]!; isotype: P [P00133593]!).
Oxydectes adenophora (Baill.) Kuntze, Revis. Gen. Pl. 2: 610. 1891. Type. Based on Croton adenophorus Baill.
Oxydectes payeriana (Baill.) Kuntze, Revis. Gen. Pl. 2: 612. 1891. Type. Based on Croton payerianus Baill.
Oxydectes tulasnei (Baill.) Kuntze, Revis. Gen. Pl. 2: 613. 1891. Type. Based on Croton tulasnei Baill.
Croton tenuicuspis Baill., Bull. Mens. Soc. Linn. Paris 2: 927. 1891. Type. Madagascar. sin. loc., *R. Baron 5846* (holotype: P [P00133364]!; isotype: K [K000422590]!).

##### Type.

Madagascar. Prov. Antsiranana: Diana Region, Nossi-bé [Nosy Be], 1837, *J.M.C. Richard 214* (lectotype, designated by Kainulainen et al. 2017b, pg. 382: P [P00123689]!; isolectotype: P [P00123690]!). Madagascar. Prov. Antsiranana: Diana Region. Nossi-bé [Nosy Be], *J.M.C. Richard 574* (syntype: P [P00123691]!); ibid. loc., 1849, *L.H. Boivin 2187* (syntype: P [P00301515]!).

##### Habit and distribution.

Shrubs or small trees; northern and northwestern Madagascar (Antsiranana, Mahajanga) and Mayotte in the Comoros Archipelago.

##### Notes.

*Croton adenophorus* was substantially recircumscribed from the concept of Leandri (1939) and Radcliffe-Smith (2016) by Kainulainen et al. (2017b). In both earlier publications, *C. loucoubensis* was treated as a synonym of *C. adenophorus*, and *C. subaemulans* was recognized as a distinct species. This earlier synonymy was due to a fundamental misunderstanding of *C. adenophorus*. See Kainulainen et al. (2017b) for further details and the distinguishing features of *C. adenophorus* and *C. loucoubensis*.

The sheet P00466148 is listed in Sonnerat under *Croton tulasnei*, but without an image. We never saw this specimen at P either before or after the herbarium renovation in 2015, and the whole folder of *C. tulasnei* was missing during visits to P in 2016 and 2017.

Radcliffe-Smith (2016) mentioned *Boivin 2187* at P as the lectotype for *Croton adenophorus* but failed to state “designated here” or an equivalent statement, so his designation was not validly published.

#### 3. Croton alchorneifolius

Taxon classificationPlantaeMalpighialesEuphorbiaceae

Radcl.-Sm., Gen. Croton Madag. Comoro 135. 2016

##### Type.

Madagascar. Prov. Antsiranana: SAVA Region, summit of Marojejy on path from Mandena, 14°26'56"S, 49°43'58"E, 30 Sep 1994, *B. Lewis*, *F. Rasoavimbahoaka* & *J. 1206* (holotype: K!; isotypes: MO!, P [P00131484]!).

##### Habit and distribution.

Small trees; restricted to the Marojejy Massif in northern Madagascar (Antsiranana).

##### Notes.

This is a distinctive high-elevation species with well-developed petals in the pistillate flowers and large capsules, belonging to the Mongue Group of Leandri (1939).

#### 4. Croton aleuritoides

Taxon classificationPlantaeMalpighialesEuphorbiaceae

P.E.Berry, Candollea 71: 182. 2016 [17 Jun 2016]

Croton bemaranus var. pseudolepidotus Radcl.-Sm., Gen. Croton Madag. Comoro 209. 2016 [23 Dec 2016], as ‘*bemarana*’, **syn. nov.** Type. Madagascar. Prov. Antsiranana: Diana Region, Montagne des Français, à l’Est de Diego Suarez, 26 Nov 1958, *Service Forestier 20088* (lectotype, designated here: P [P00312410]!; isolectotypes: B, G [G00341674]!, K [K001040349]!, MO!, P [P00206489]!, S!, TEF!, WAG).

##### Type.

Madagascar. Prov. Antsiranana: Diana Region, Montagne des Français, à l’Est de Diégo-Suarez, 26 Nov 1958, *Service Forestier 20088* (holotype: P [P00312410]!; isotypes: B, G [G00341674]!, K [K001040349]!, MO!, P [P00206489]!, S!, TEF!, WAG).

##### Habit and distribution.

Trees; known only from Montagne des Français in northernmost Madagascar (Antsiranana).

#### 5. Croton ambanivoulensis

Taxon classificationPlantaeMalpighialesEuphorbiaceae

Baill., Adansonia 1: 165. 1861, as ‘ambanivoulense’

Oxydectes ambanivoulensis (Baill.) Kuntze, Revis. Gen. Pl. 2: 611. 1891. Type. Based on Croton ambanivoulensis Baill.

##### Type.

Madagascar. Prov. Toamasina: [region of the] Ambanivoules, [west of] Tamatave, dans les forêts, Dec. 1836, *J.P. Goudot s.n.* (lectotype, designated here: G [G00446336]!; isolectotype: P [P00301514]!).

##### Habit and distribution.

Shrubs; eastern lowland Madagascar (Toamasina).

##### Notes.

There are two sheets of *Goudot s.n.* at G within the same jacket, meaning they are considered as being part of the same collection (see Gautier et al. 2016). However, they do not fit the criterion of bearing a single label in common. Each sheet has its own label, and they are not identical. One, the lectotype, has a precise collecting date and the locality name of Ambanivoules, whereas the other sheet (G00446337) does not, and the written field description of the plants differs between the two. Also, the lectotype has mainly pistillate flowers open whereas the other syntype has only staminate flowers open. This leads us to conclude that the two sheets are actually different gatherings, even though both were annotated in Baillon's hand as “*Croton ambanivoulense*.” We have designated the sheet that is most consistent with the protologue (G00446336) as the lectotype.

Ambanivoules were an ethnic group of eastern Madagascar located approximately 80–100 km west of Tamatave, their name derived from the Malagasy “Antanbanivolo,” or “people living at the base of the mountains covered with bamboos” (Schatz 2013).

#### 6. Croton ambovombensis

Taxon classificationPlantaeMalpighialesEuphorbiaceae

Radcl.-Sm. & Govaerts, Kew Bull. 52: 186. 1997

Croton divaricatus Leandri, Ann. Mus. Colon. Marseille, sér. 5, 7(1): 59. 1939, as ‘*divaricata*’, nom. illeg. non Croton divaricatus Sw., 1788.

##### Type.

Madagascar. Prov. Toliara: Androy Region, Ambovombe, 19 Dec 1924, *R. Decary 3174* (lectotype, designated by Radcliffe-Smith 2016, pg. 191: P [P00133060]!; isolectotype: TAN [TAN000529]!). Madagascar. Prov. Toliara: Androy Region, environs d’Ambovombe (extrème sud), 9 Sep 1928, *H. Humbert & C.F. Swingle 5631* (syntypes: G [G00446382]!, P [P00133062]!; Madagascar. Prov. Toliara: Andrahomana, 21 Jun 1926, *R. Decary 4017* (syntype: P [P00133061]!).

##### Habit and distribution.

Shrubs; southern lowland Madagascar (Toliara).

#### 7. Croton androiensis

Taxon classificationPlantaeMalpighialesEuphorbiaceae

(Leandri) Leandri, Adansonia, n.s., 9: 507. 1970

Croton geayi var. androiensis Leandri, Ann. Mus. Colon. Marseille, sér. 5, 7(1): 35. 1939. Type. Madagascar. Prov. Toliara: Androy/Anosy Regions, Bevilany, limite entre l’Anosy et l’Androy, 14 Nov 1932, *R. Decary 10940* (lectotype, designated here): P [P00123697]!; isolectotypes: G, K [K001044841]!, TAN [TAN000533]!). Madagascar. Prov. Toliara: Androy Region, Ambovombe, 6 Jan 1931, *R. Decary 8361* (syntypes: P [P00123696]!), TAN [TAN000532]!). Madagascar. Prov. Toliara: Kotoala, sables et dunes littorales, 21 Jan 1931, *R. Decary 8408* (syntypes: K [K001040395]!, MO [sheet #04861163]!, P [P00404477]!, P [P00123698]!).

##### Type.

Based on Croton geayi var. androiensis Leandri

##### Habit and distribution.

Shrubs; southern Madagascar (Toliara).

##### Notes.

Radcliffe-Smith (2016) mentioned *Decary 10940* as the lectotype for Croton geayi var. androiensis, but this is not validly published as he failed to state “designated here” or an equivalent phrase.

#### 8. Croton anisatus

Taxon classificationPlantaeMalpighialesEuphorbiaceae

Baill., Adansonia 1: 159. 1861, as ‘anisatum’

Oxydectes anisata (Baill.) Kuntze, Revis. Gen. Pl. 2: 611. 1891. Type. Based on Croton anisatus Baill.

##### Type.

[Cult. ex Madagascar]: Cultivated in the garden of M. Hubert, Saint Benoît, Réunion, s.d., *M. Lepervanche s.n.* (holotype: P [P00301513]!). Madagascar. Prov. Toamasina: Atsinanana Region, Vohibola, N to NNW of village of Andranokoditra, N of Lac Ampitabe, 18°33'34"S, 49°15'01"E, 5 m, 12 Feb 2003, *P. Lowry, R. Rabevohitra, J. Rabenantoandro, R. Razakamalala & S.W.J. Lowry 6072* (epitype, designated here): P [P00548219]!; additional duplicates: DAV!, K!, MICH [MICH1210799]!, MO [sheet # 5902002]!).

##### Habit and distribution.

Shrubs; eastern coast of Madagascar (Toamasina).

##### Notes.

Since the holotype of *Croton anisatus* has only young inflorescences in bud and is known only from cultivation on a quite different island, we designate an epitype with open flowers. This littoral species can be characterized by its very congested inflorescences and the pseudoverticillate, anisophyllous, and sparsely lepidote-pubescent leaves with an entire margin. The plant is apparently quite aromatic as indicated by the descriptions of both the holotype and epitype, as well as by its specific epithet.

#### 9. Croton ankarensis

Taxon classificationPlantaeMalpighialesEuphorbiaceae

Leandri, Ann. Mus. Colon. Marseille, sér. 5, 7(1): 75. 1939

##### Type.

Madagascar. Prov. Mahajanga: Causse d’Ankara, bois rocailleux et secs sur calcaire jurassique, Dec 1900, *H. Perrier de la Bâthie 9830* (lectotype, designated here: P [P00123702]!; isolectotype: P [P00123703]!). Madagascar. Prov. Mahajanga: Tsingy du Bemaraha (9^e^ Réserve), 4 Oct 1932, *J. Leandri 103bis* (syntypes: K [K001040394]!, P [P00123700]!, P [P00123701]!).

##### Habit and distribution.

Shrubs; western Madagascar (Mahajanga).

##### Notes.

The *Leandri 103bis* syntype corresponds to *Croton tsiampiensis*.

#### 10. Croton ankeranae

Taxon classificationPlantaeMalpighialesEuphorbiaceae

Kainul., Candollea 71: 329. 2016

##### Type.

Madagascar. Prov. Toamasina: Atsinanana Region, District Brickaville, Commune Maroseranana, Fokontany Ambodilendemy, Andrangato River, 18°26'37"S, 48°46'31"E, 446 m, 13 Mar 2011, *P. Antilahimena 7554* (holotype: MICH [MICH1513200]!; isotypes: MO!, P!, TAN!).

##### Habit and distribution.

Shrubs to small trees; eastern Madagascar (Toamasina).

#### 11. Croton anosiravensis

Taxon classificationPlantaeMalpighialesEuphorbiaceae

Leandri, Adansonia, n.s., 12: 69. 1972

Croton anosiravensis var. pilosus Radcl.-Sm., Gen. Croton Madag. Comoro 207. 2016, **syn. nov.** Type. Madagascar. Prov. Antsiranana: Analamera, 50-400 m, Jan 1938, *H. Humbert 19141* (lectotype, designated here: P [P00133307]!; isolectotype: P [P00133308]!).

##### Type.

Madagascar. Prov. Antsiranana: Base des escarpements de l’Anosiravo, poteau kilométrique 6 de la route de Diego Suarez à Orangéa, 12 Dec 1963, *Service Forestier 22930*-*SF* (holotype: P [P00404432]!; isotypes: K [K001040369]!, TEF [TEF000193]!).

##### Habit and distribution.

Shrubs; northern Madagascar (Antsiranana).

##### Notes.

In its stellate-pubescent and cordate leaves, *Croton anosiravensis* is superficially similar to some of the species in the Adenophorus Group. However, it does not have opposite leaves or laminar glands, and is probably not closely related. It appears to be a rare species, because besides the type from the northern slopes of Montagne des Français, it is otherwise only known from Analamera (*Humbert 19141* [P]) and Befarafara in Daraina (*Rakotonandrasana et al. 1048* [CNARP, MICH, MO, P, TAN]). Radcliffe-Smith (2016) stated “holo: P” for var. pilosus, but there are two sheets of the type collection there, so we have selected the more complete of the sheets at P as the lectotype.

#### 12. Croton antanosiensis

Taxon classificationPlantaeMalpighialesEuphorbiaceae

Leandri, Ann. Mus. Colon. Marseille, sér. 5, 7(1): 45. 1939

##### Type.

Madagascar. Prov. Toliara: environs de Fort-Dauphin, près de Bévilany, 200-300 m, 14 Sep 1928, *H. Humbert & C.F. Swingle 5695* (lectotype, designated here: P [P00123716]!; isolectotype: P [P00123717]!). Madagascar. Prov. Toliara: Massif de Beampingaratra (Sud-Est), du col de Bevava au sommet de Bekoho, forêt sur latérite de gneiss, 1100-1500 m, 6-7 Nov 1928, *H. Humbert 6416* (syntypes: P [P00123718]!, P [P00154301]!, TAN [TAN000521]!). Madagascar. Prov. Toliara: Fort Dauphin, *J. Cloisel 60* (syntype: P [P00123710]!). Madagascar. Prov. Toliara: Behara, 9 Jul 1926, *R. Decary 4321* (syntypes: P [P00123711]!, S [S07-14102]). Madagascar. Prov. Toliara: Ranofotsy, 29 Jul 1932, *R. Decary 10175* (syntypes: G [G00446368]!, P [P00123712]!).

##### Habit and distribution.

Shrubs to small trees; southern Madagascar (Toliara).

##### Notes.

We believe that the lectotype chosen here best conforms to the protologue among the syntypes cited by Leandri (1939). Although most of the other syntypes correspond to the same species, one of them, *Humbert 6416*, appears to belong instead to *C. trichotomus*.

#### 13. Croton argyrodaphne

Taxon classificationPlantaeMalpighialesEuphorbiaceae

Baill., Adansonia 1: 146. 1861

Oxydectes argyrodaphne (Baill.) Kuntze, Revis. Gen. Pl. 2: 611. 1891. Type. Based on Croton argyrodaphne Baill.
Croton argyrodaphne var. occidentalis Leandri, Ann. Mus. Colon. Marseille, sér. 5, 7(1): 44. 1939, **syn. nov.** Type. Madagascar. Prov. Mahajanga: région du Cap Saint-André, dunes, 8 Jun 1930, *R. Decary 7893* (lectotype, designated here: P [P00127457]!). Madagascar. Prov. Toliara: bassin de la Tsiribihina, Jul 1911, *H. Perrier de la Bâthie 9656* (syntypes: P [P00127460]!, P [P00127461]!, P [P00127462]!).] !). Madagascar. Prov. Toliara: Morondava, received 26 Dec 1878, *H. Grevé 11* (syntype: P [P00127459]!).
Croton argyrodaphne var. boinensis Leandri, Adansonia, sér. 2, 12: 404. 1972, **syn. nov.** Type. Madagascar. Prov. Mahajanga: Ampalony, 9 Aug 1971, *L.P. Schmitt 515* (holotype: P [P00389629]!).
Croton argyrodaphne var. orientalis Leandri, Adansonia, sér. 2, 12: 404. 1972, **syn. nov.** Type. Madagascar. Prov. Toamasina: Intendro, près de Fénérive, 9 Jul 1958, *Service Forestier 19160-SF* (holotype: P [P00418607]!).
Croton vohemarensis Radcl.-Sm., Gen. Croton Madag. Comoro 78. 2016, **syn. nov.** Type. Madagascar. Prov. Antsiranana: Mantamena, Bekaroaka Range, 7 km N of Daraina (Vohemar), 13°08'S, 49°42'E, 112-330 m, 26 Nov 1990, *D. Meyers 206* (holotype: MO!).

##### Type.

Madagascar. Prov. Antsiranana: Diana Region, Nossibé, 1837, *J.M.C. Richard 218* (lectotype, first step designated by Leandri 1972b, pg. 404, second step designated here): P [P00127450]!; isolectotype: K [K001040357]!). Madagascar. Prov. Antsiranana: Diana Region, Nossibé, *L.M.A. Du Petit-Thouars s.n.* (syntype: P [P00123733]!), ibid. loc., 1837, *J.M.C. Richard 571* (syntypes: P [P00127451]!, K [K000347495]!), ibid. loc., 15 Nov 1840, *A. Pervillé 236* (syntypes: K [K000347493]!, P [P00127436]!, P [P00127437]!). Madagascar. Prov. Antsiranana: Loucoubé, 1848, *L.H. Boivin 2182* (syntypes: G-DC [G00311741]!, G [G00311741]!, K [K000347494]!, P [P00123729]!, P [P00123730]!, P [P00835716]!).

##### Habit and distribution.

Trees to large shrubs; mainly in northern Madagascar, but extending as far south as northern Toliara Province on the west coast and northern Toamasina on the east coast (Antsiranana, Mahajanga, Toamasina, Toliara).

##### Notes.

Leandri (1972b) designated *Richard 218* as the type of *Croton argyrodaphne*. Given that he did not specify an herbarium in his selection of this collection, we complete the lectotypification here by designating P00127450 as a second-step lectotype. The type of C. argyrodaphne var. orientalis comes from an area in northern Toamasina that is well south of the range of most other *C. argyrodaphne* specimens. However, there is a second collection of the species from the same area, *SF-10816* (TEF), which confirms its occurrence near Fénérive.

The type of *Croton vohemarensis* consists of two small twigs with leaves that are unusually small and wide for *C. argyrodaphne*. However, the low stamen number (11) and the characteristic stylar column are very typical of *C. argyrodaphne* (see Fig. 1E–F), and it falls within the geographic and altitudinal range for the species. An additional paratype cited by Radcliffe-Smith (2016), *Meyers & Bolz 170* (G, MO), comes from the type locality and is a tree 7 m tall, again with unusually wide and long-petiolate leaves for *C. argyrodaphne*, but it only has young floral buds.

What Leandri recognized as Croton argyrodaphne var. occidentalis is a rather distinctive element of this species, with a western, subcoastal distribution. The plants are small trees, and the leaves have yellowish pigmentation along the midvein on the adaxial leaf surfaces, but other than that they conform well to the general aspect of *C. argyrodaphne*.

#### 14. Croton aubrevilecta

Taxon classificationPlantaeMalpighialesEuphorbiaceae

Leandri, Adansonia, sér. 2, 10: 309. 1970

##### Type.

Madagascar. Prov. Toliara: Sud-Ouest, s.d., *M.G. Cours 4641* (holotype: P [P00312369]!; isotypes: K [K001044848], MO [sheet # 5737746]!), P [P00380443]!, P [P00380444]!).

##### Habit and distribution.

Shrubs; southwestern Madagascar (Toliara).

##### Notes.

Leandri (1970b) designated *Cours 4641* as the holotype of *C. aubrevilecta* but there are three sheets of this collection at P. One of them, P00312369, has a label in Leandri's handwriting saying “type,” and the other two duplicates at P have labels stating “isotype.” The isotypes at P have preprinted labels stating “Itinéraire de Didy à Brickaville (forêt orientale),” but P00380444 has a note added later stating “Localité très douteuse, voir récolte *Homolle 1944* (avec *M.G. Cours*).” The holotype has a penciled note stating “Probablement région de Tranoroa.” In the protologue, Leandri (1970b) also alluded to the erroneous labels from Didy and states that the collection likely came from far southern Madagascar close to Lake Tsimanampetsotsa.

#### 15. Croton barorum

Taxon classificationPlantaeMalpighialesEuphorbiaceae

Leandri, Ann. Mus. Colon. Marseille, sér. 5, 7(1): 66. 1939

##### Type.

Madagascar. Prov. Toliara: Antanimora, 16 Jun 1926, *R. Decary 4346* (holotype: P [P00301486]!; isotype: K [K001044842]!).

##### Habit and distribution.

Shrubs; southwestern Madagascar (Toliara).

#### 16. Croton basaltorum

Taxon classificationPlantaeMalpighialesEuphorbiaceae

(Leandri) P.E.Berry, comb. et stat. nov.

urn:lsid:ipni.org:names:77167302-1

Croton antanosiensis var. basaltorum Leandri, Ann. Mus. Colon. Marseille, sér. 5, 7(1): 46. 1939. Type. Madagascar. Prov. Mahajanga: Pl. [Plateau] d’Antanimena, entre la Mahavavy et le Betsiboka, Jun 1906, *H. Perrier de la Bâthie 9593* (lectotype, designated here: P [P00123726]!; isolectotype: P [P00123725]!).

##### Type.

Based on Croton antanosiensis var. basaltorum Leandri

##### Habit and distribution.

Shrubs; western Madagascar (Mahajanga).

##### Notes.

Geographically and morphologically, this species is sufficiently distinct from *C. antanosiensis* to merit recognition at the species level. Morphologically, it appears more similar to *C. cupreolepis*.

#### 17. Croton bastardii

Taxon classificationPlantaeMalpighialesEuphorbiaceae

Leandri, Ann. Mus. Colon. Marseille, sér. 5, 7(1): 64. 1939, as ‘bastardi’

Croton appertii Leandri, Adansonia, sér. 2, 15: 331. 1976, **syn. nov.** Type. Madagascar. Prov. Toliara: Beharana, près de Manja, 300 m, 17 Nov 1961, *O. Appert 37* (holotype: P [P00312368]!; isotypes: K [K001044847]!, MO [sheet # 2287361]!, Z [Z-000015970]!, Z [Z-000015971]!).

##### Type.

Madagascar. Prov. Toliara: forêt de Besomaty, entre le Fiherena et l’ (Mangoky), 750-800 m, Dec 1933, *H. Humbert 11236* (lectotype, designated here: P [P00404492]!; isolectotypes: K [K001044843]!, P [P00127500]!).

##### Habit and distribution.

Shrubs; southwestern Madagascar (Mahajanga, Toliara).

#### 18. Croton bathianus

Taxon classificationPlantaeMalpighialesEuphorbiaceae

Leandri, Ann. Mus. Colon. Marseille, sér. 5, 7(1): 80. 1939, as ‘bathiana’

##### Type.

Madagascar. Prov. Mahajanga: Haut Bemarivo, Oct 1907, *H. Perrier de la Bâthie 9545* (lectotype, designated by Kainulainen et al. 2017b, p. 386: P [P00301483]!; isolectotype: P [P00127503]!). Madagascar. Prov. Mahajanga: collines sèches de haut Bemarivo, Dec 1906, *H. Perrier de la Bâthie 9633* (syntype: P [P00389630]!). Madagascar. Prov. Mahajanga: Maromandia, presqu’île Radama, 13 Oct 1922, *R. Decary 1133* (syntype: P [P00389631]!), ibid. loc., 11 Oct 1922, *R. Decary 1174* (syntype: P [P00301482]!).

##### Habit and distribution.

Shrubs; northwestern Madagascar (Antsiranana, Mahajanga).

#### 19. Croton bemaranus

Taxon classificationPlantaeMalpighialesEuphorbiaceae

Leandri, Ann. Mus. Colon. Marseille, sér. 5, 7(1): 69. 1939, as ‘bemarana’

Croton subaemulans var. tsingyensis Radcl.-Sm., Gen. Croton Madag. Comoro 120. 2016, **syn. nov.** Type. Madagascar. Prov. Mahajanga: Tsingy du Bemaraha (9e Réserve), reçu Feb -Apr 1933, *J. Leandri 116* (holotype: K!; isotypes: P [P00133368!, P00133369!]).

##### Type.

Madagascar. Prov. Mahajanga: causse d’Ankara, Oct 1900, *H. Perrier de la Bâthie 1153* (lectotype, designated here: P [P00206491]!; isolectotypes: K [K001040396]!, P [P00389502]!). Madagascar. Prov. Mahajanga: Bemara, partie Nord de l’Antsingy, entre Ambatomiloloha et Anjohinomby, 29 Nov 1932, *J. Leandri 656* (syntypes: P [P00127512]!, P [P00389503]!).

##### Habit and distribution.

Shrubs; western and northern Madagascar (Antsiranana, Mahajanga).

#### 20. Croton bemarivensis

Taxon classificationPlantaeMalpighialesEuphorbiaceae

Leandri, Adansonia, sér. 2, 13: 423. 1974

##### Type.

Madagascar. Prov. Antsiranana: entre Andrangana et la rivière Anjambazamba, route de Sambava à Antsirabe-Nord, 2-7 Oct 1966, *Service Forestier 27197-SF* (holotype: P [P00312371!; isotypes: K [K001044846]!, MO [sheet #04861161]!, P [P00404483]!, TEF [TEF000192]!).

##### Habit and distribution.

Shrubs; northeastern Madagascar (Antsiranana, Toamasina).

#### 21. Croton bergassae

Taxon classificationPlantaeMalpighialesEuphorbiaceae

Leandri, Adansonia, sér. 2, 13: 176. 1973

##### Type.

Madagascar. Prov. Toamasina: Menagisy, Brickaville, 11 Oct 1956, *Service de Eaux et Forêts de Madagascar 12358-SF* (lectotype, designated here: P [P00312375]!; isolectotypes: P [P00127519]!, TEF!).

##### Habit and distribution.

Shrubs or small trees; eastern lowland Madagascar (Antsiranana, Toamasina).

##### Notes.

Although the sheet designated here as lectotype has a sticker stating “TYPE” and the other sheet at P has one stating “ISOTYPE,” it is not clear who applied those labels and if it was done after Leandri's publication. In any case, there is no annotation in Leandri's hand on either sheet to indicate which of the two he intended to be the holotype.

#### 22. Croton bernieri

Taxon classificationPlantaeMalpighialesEuphorbiaceae

Baill., Adansonia 1: 152. 1861

Oxydectes bernieri (Baill.) Kuntze, Revis. Gen. Pl. 2: 611. 1891. Type. Based on Croton bernieri Baill.
Croton bernieri var. namorokensis Leandri, Ann. Mus. Colon. Marseille, sér. 5, 7(1): 54. 1939, **syn. nov.** Type. Madagascar. Prov. Mahajanga: Tsingy de Namoroka (8^e^ Réserve), 1933, *Service Forestier 11* (holotype: P [P00127543]!).

##### Type.

Madagascar. Prov. Antsiranana: Diégo-Suarez, 1835, *A.C.J. Bernier 306* (lectotype, designated here: P [P00127520]!; isolectotypes: G [G00434420]!, P [P00127521]!, P [P00301481]!). Madagascar. Prov. Antsiranana: Baie de Diégo-Suarez, Dec 1848, *L.H. Boivin 2657* (syntypes: G [G00311744]!, G-DC [G00311744]!, P [P00127522]!, P [P00127523]!, P [P00127524]!, P [P00301480]!).

##### Habit and distribution.

Shrubs to small trees; northern and northwestern Madagascar (Antsiranana, Mahajanga).

#### 23. Croton betiokensis

Taxon classificationPlantaeMalpighialesEuphorbiaceae

Leandri, Adansonia, sér. 2, 10: 183. 1970

##### Type.

Madagascar. Prov. Toliara: Plateau Mahafaly à l’ouest de Betioky, 100-300 m, 17-20 Mar 1955, *H. Humbert & R. Capuron 29489* (holotype: P [P00312386]!; isotypes: P [P00127545], P [P00127546]!).

##### Habit and distribution.

Shrubs; southern Madagascar (Toliara).

#### 24. Croton bifurcatus

Taxon classificationPlantaeMalpighialesEuphorbiaceae

Baill., Adansonia 1: 164. 1861, as ‘bifurcatum’

Croton bifurcatus var. genuinus Müll.Arg. in A.P.de Candolle, Prodr. 16(2): 584. 1866, nom. inval.
Oxydectes bifurcata (Baill.) Kuntze, Revis. Gen. Pl. 2: 611. 1891. Type. Based on Croton bifurcatus Baill.
Croton bifurcatus var. humblotii Leandri ex Radcl.-Sm., Gen. Croton Madag. Comoro 25. 2016, **syn. nov.** Type. Mayotte [French Overseas Department]: forêt de Mazé, M. Bini [Majimbini], 24 May 1884, *L. Humblot 1162* (holotype: K; isotype: P [P00196067]!).

##### Type.

Mayotte [French Overseas Department]: Cascade du Msapéré, 1849, *L.H. Boivin 3380* (holotype: P [P00196066]!; isotypes: G-DC [G00311982]!), G [G00446373]!, P [P00196065]!, P [P00466147]!).

##### Habit and distribution.

Shrubs; known only from the French island of Mayotte in the Comoro Islands.

##### Notes.

The type of C. bifurcatus var. humblotii differs in only minor ways from typical *C. bifurcatus*.

#### 25. Croton bocquillonii

Taxon classificationPlantaeMalpighialesEuphorbiaceae

Baill., Adansonia 1: 161. 1861, as ‘bocquilloni’

Oxydectes bocquillonii (Baill.) Kuntze, Revis. Gen. Pl. 2: 611. 1891. Type. Based on Croton bocquillonii Baill.
Croton brevispicatus var. bocquillonii (Baill.) Leandri, Ann. Mus. Colon. Marseille, sér. 5, 7(1): 27. 1939. Type. Based on Croton bocquillonii Baill.

##### Type.

Madagascar. Prov. Mahajanga: Ambongo, 16 Feb 1841, *A. Pervillé 648* (lectotype, designated here: P [P00127552]!; isolectotypes: P [P00127553]!, P [P00127554]!, P [P00131420]!).

##### Habit and distribution.

Shrubs; western Madagascar (Mahajanga).

##### Notes.

The sheet chosen here as lectotype bears two labels, one on the left that lists *Pervillé 648* as the collector, and one on the right in Baillon's hand that seemingly attributes the collection to Boivin, stating “ex Ambongo, cum cl. Pervillé et Bernier comm. (1846).” Baillon lists this in his protologue as a separate collection, but it looks identical to the other syntypes, so we believe it is actually part of the same collection by Pervillé.

The types lack pistillate flowers, but they are distinctive from *C. brevispicatus* in bearing a pair of sessile acropetiolar glands and in having relatively long petioles for the size of the leaf, and blades with a rounded-cuneate base and an acuminate apex.

#### 26. Croton boinensis

Taxon classificationPlantaeMalpighialesEuphorbiaceae

Leandri, Ann. Mus. Colon. Marseille, sér. 5, 7(1): 29. 1939

##### Type.

Madagascar. Prov. Mahajanga: Bongolava, Boïna, Nov 1906, *H. Perrier de la Bâthie 9567* (lectotype, designated here: P [P00127563]!). Madagascar. Prov. Mahajanga: Ankarafantsika, 7^e^ reserve, chemin de Ste. Marie, Ankorika, 150-200 m, *Service Forestier Madagascar 154* (syntypes: K [K001040380]!, P [P00127566]!). Madagascar. Prov. Mahajanga: environs de Madirovalo, Boïny, Nov 1902, *H. Perrier de la Bâthie 9803* (syntypes: P [P00127564!], P [P00127565]!. Madagascar. sin. loc., *Service Forestier Madagascar 64* (syntype: P [P00127567]!)).

##### Habit and distribution.

Shrubs; western Madagascar (Mahajanga).

#### 27. Croton boiteaui

Taxon classificationPlantaeMalpighialesEuphorbiaceae

Leandri, Adansonia, sér. 2, 10: 313. 1970

Croton mavoravina var. concinnus Radcl.-Sm., Gen. Croton Madag. Comoro 31. 2016, **syn. nov.** Type. Madagascar. Prov. Toliara: environs d’Ampandrandava, entre Bekily et Tsivory, 1000 m, Nov 1942, *Herbier du Jardin Botanique de Tananarive 5730* (holotype: P [P00154405]!).

##### Type.

Madagascar. Prov. Toliara: environs de Bekily, 12 Oct 1966, *P. Boiteau 384* (lectotype, designated here: P [P00312385]!; isolectotypes: P [P00131001]!, P [P00131002]!).

##### Habit and distribution.

Shrubs; southern Madagascar (Toliara).

#### 28. Croton boivinianus

Taxon classificationPlantaeMalpighialesEuphorbiaceae

(Baill.) Baill., Adansonia 1: 163. 1861, as ‘boivinanum’

Furcaria boiviniana Baill., Étude Euphorb.: 356. 1858. Type. Madagascar. Prov. Antsiranana: Diana Region, Ile Nossibé, 1841, *A. Pervillé 267* (lectotype, designated here: P [P00312458]!; isolectotypes: G [G00446376]!, P [P00131010]!). Madagascar. Prov. Antsiranana: Diana Region, Nossibé, 1847-1852, *L.H. Boivin 2183* (syntype: P [P00312459]!).
Oxydectes boiviniana (Baill.) Kuntze, Revis. Gen. Pl. 2: 611. 1891. Type. Based on Furcaria boiviniana Baill.

##### Type.

Based on *Furcaria boiviniana* Baill.

##### Habit and distribution.

Shrubs; northern Madagascar (Antsiranana).

##### Notes.

Baillon (1858) did not cite any specific specimens in the description of *Furcaria boiviniana*, just “*F. boiviniana* (herb. Mus.).” Baillon (1861) cited *Boivin 2183* and *Pervillé 267* when he transferred the taxon to *Croton*. Both specimens are annotated in Baillon's hand as “Croton (Furcaria) boivinianum H. Bn., Et. Gen. Euph., 356,” so we therefore consider them to be original material for the taxon.

#### 29. Croton bojerianus

Taxon classificationPlantaeMalpighialesEuphorbiaceae

Baill., Adansonia 1: 151. 1861, as ‘bojerianum’

Croton bakerianus Baill., Bull. Mens. Soc. Linn. Paris 2: 849. 1890, as ‘*bakeriana*’. Type. Madagascar. sin. loc., s.d., *R. Baron 2091* (holotype: K [K001040351]!; isotype: P [P0013152]!).
Oxydectes bojeriana (Baill.) Kuntze, Revis. Gen. Pl. 2: 611. 1891. Type. Based on Croton bojerianus Baill.

##### Type.

Madagascar. sin. loc., s.d., *W. Bojer s.n.* (holotype: P [P00131353]!; isotypes: K [K000347489]!, M [M0110365]!).

##### Habit and distribution.

Shrubs; central highland Madagascar (Antananarivo, Fianarantsoa, possibly Mahajanga).

#### 30. Croton bonplandianus

Taxon classificationPlantaeMalpighialesEuphorbiaceae

Baill., Adansonia 4: 339. 1864, as ‘bonplandianum’

##### Type.

Argentina. Prov. Corrientes: 1833, *A. Bonpland s.n.* (lectotype, designated here: P [P00623061]!; isolectotype: P [P00623060]!). Paraguay: Apr-May 1845, *H.A. Weddell 3207* (syntypes: P [P00623063]!, P [P00623062]!).

##### Habit and distribution.

Herbs to subshrubs; native to southern South America, but naturalized in the Mascarene islands of Mauritius, Réunion, and Rodrigues (Croizat 1944, Coode 1982), as well as on Mayotte (Grande Terre – Mamoudzou, Kawéni, 9 Sep 2005, *Barthelat & Changama 1504*, K).

##### Notes.

*Croton bonplandianus* is currently the only non-native, naturalized species of *Croton* in the Western Indian Ocean Region. Oddly, it is known from Mayotte and the Mascarenes, but it has not yet been observed or collected in Madagascar.

#### 31. Croton bracteatus

Taxon classificationPlantaeMalpighialesEuphorbiaceae

Lam., Encycl. 2: 208. 1786, as ‘bracteatum’

Andrichnia bracteata (Lam.) Baill., Étude. Euphorb. 362. 1858. Type. Based on Croton bracteatus Lam.
Oxydectes bracteata (Lam.) Kuntze, Revis. Gen. Pl. 2: 611. 1891. Type. Based on Croton bracteatus Lam.
Croton ivohibensis var. puncticulatus Radcl.-Sm., Gen. Croton Madag. Comoro 150. 2016, **syn. nov.** Type. Madagascar. Prov. Fianarantsoa: Farafangana, Réserve spéciale de Manombo, 23°03'42"S, 47°44'26"E, 30 m, 25 Aug 1995, *P. Rakotomalaza et al. 452* (holotype: K!; isotype: MO!).

##### Type.

Madagascar. sin. loc., s.d., *P. Commerson s.n.* (holotype: P-LA [P00382050]!; isotypes: G-DC [G00311196]!, G [G00446380]!, MPU [MPU014765]!, MPU [MPU014766]!, P [P00312457]!; probable isotype: P-JU (P00131377]!).

##### Habit and distribution.

Shrubs or small trees; eastern coastal Madagascar (southern Fianarantsoa).

##### Notes.

The collection locality of the type of *Croton bracteatus* is uncertain. Although most of Commerson's collections from Madagascar came from the Fort Dauphin area in Toliara Province, the type of *C. bracteatus* matches well specimens from the Manombo Reserve south of Farafangana in Fianarantsoa Province.

#### 32. Croton brevispicatus

Taxon classificationPlantaeMalpighialesEuphorbiaceae

Baill., Adansonia 1: 152. 1861, as ‘brevispicatum’

Croton brachybotryus Müll.Arg., Prodr. 15(2): 571. 1866, nom. superfl. Type. Based on Croton brevispicatus Baill.
Oxydectes brevispicata (Baill.) Kuntze, Revis. Gen. Pl. 2: 609. 1891. Type. Based on Croton brevispicatus Baill.

##### Type.

Madagascar. Prov. Antsiranana: [Diana Region] côte orientale, Baie de Rigny, Dec 1848, *L.H. Boivin 2658* (holotype: P [P00131378]!; isotypes: G [G00018191]!, G-DC [G00311751]!), P [P00131406]!).

##### Habit and distribution.

Shrubs; northern and western Madagascar (Antsiranana, Mahajanga).

##### Notes.

Boivin's collection number 2658 was applied to several different collection events. Baillon (1861) was apparently aware of this given the greater detail in which he cited Boivin's specimens, such as “Boivin (1848), n. 2658, Madag., baie de Rigny (h. Mus.)” under *Croton brevispicatus* and “Boivin, n. 2658, cap d’Ambre” under *C. squamiger* Baill. Müller (1866) cited *C. brevispicatus* Baill., as well as the type of that species (“Boivin n. 2658! in hb. Mus. Paris”), in his treatment of *C. brachybotryus*, which we interpret as an illegitimate name for *C. brevispicatus*. The isotype at P was a gift from the Caen herbarium received by the Paris herbarium in 1974, and it was never annotated by Baillon, although it had been annotated by Müller.

#### 33. Croton campenonii

Taxon classificationPlantaeMalpighialesEuphorbiaceae

Baill., Bull. Mens. Soc. Linn. Paris 2: 847. 1890, as ‘campenoni’

##### Type.

Madagascar. “Madagascar central,” *P. Campenon s.n*. (lectotype, designated here: P [P00389501]!; isolectotype: P [P00131482]!).

##### Habit and distribution.

Trees; central and northern upland Madagascar (Antananarivo, Antsiranana, Fianarantsoa, Mahajanga).

#### 34. Croton cassinoides

Taxon classificationPlantaeMalpighialesEuphorbiaceae

Lam., Encycl. 2: 211. 1786

Oxydectes cassinoides (Lam.) Kuntze, Revis. Gen. Pl. 2: 610. 1891. Type. Based on Croton cassinoides Lam.
Croton delphinianus Baill., Bull. Mens. Soc. Linn. Paris 2: 928. 1891. Type. Madagascar. Prov. Toliara: Fort Dauphin, s.d., *G.F. Scott-Elliot 1557* (lectotype, designated here: P [P00133052]!). Madagascar. Prov. Toliara: Fort Dauphin, s.d., *G.F. Scott-Elliot s.n.* (possible original material: P [P00131487]!).

##### Type.

Madagascar. sin. loc., s.d., *P. Commerson s.n.* (lectotype, designated here: P [P00131494]!; isolectotypes: G-DC [G00311976]!, MPU [MPU014767]!), P-JU [Catal. 16372]!).

##### Habit and distribution.

Shrubs; southeastern coastal Madagascar (Toliara).

##### Notes.

Unlike most other Malagasy *Croton* species described by Lamarck from Commerson specimens, there is no specimen of *C. cassinoides* found in the Lamarck Herbarium at P, so we designate the sheet in the general herbarium at P as lectotype. Most of Commerson's collections from Madagascar came from the Fort Dauphin area in Toliara Province, and all other specimens of *C. cassinoides* that we have determined are from near Fort Dauphin, so this is likely where the type came from. In the description of *C. delphinianus* (Baillon 1891a), there was no collection or locality cited (it was the last in the section of Baillon's text and appeared to be cut off). In the descriptions of other new species in the same publication, the Latin description was always followed by a paragraph containing specimen information. At P there is a sheet [P00133052] that has a label with a note in ink stating “*Croton cassinoides* Lamk. et type du *C. Delphinianus* H. Bn.” It also has a small envelope that reads “*Croton Delphinianus* Scott-Elliot. n. 1557 Fort-Dauphin.” Based on that information, we designate it as lectotype for *C. delphinanus*.

#### 35. Croton catatii

Taxon classificationPlantaeMalpighialesEuphorbiaceae

Baill., Bull. Mens. Soc. Linn. Paris 2: 851. 1890, as ‘catati’

Croton hilaris Baill., Bull. Mens. Soc. Linn. Paris 2: 927. 1891. Type. Madagascar. Prov. Toliara: Fort-Dauphin, *G.F. Scott-Elliot 2650* (holotype: P [P00131522]!; isotype: K [K001040342]!).
Croton ranohirae Leandri, Adansonia, sér. 2, 9: 502. 1970, **syn. nov.** Type. Madagascar. Prov. Toliara: Plateaux et vallées de l’Isalo à l’Ouest de Ranohira, 900 m, 2 Nov-4 Dec 1946, *H. Humbert 19591* (lectotype, designated here: P [P00312378]!; isolectotypes: K [K000422598]!, K [K000422599]!, P [P00389515]!, P [P00389516]!).
Croton boinensis var. tomentosus Radcl.-Sm., Gen. Croton Madag. Comoro 78. 2016, **syn. nov.** Type. Madagascar. “NW Madagascar”, sin. loc. cert., recd. Sep 1887, *R. Baron 5477* (holotype: K!).
Croton catatii var. setosus Radcl.-Sm., Gen. Croton Madag. Comoro 45. 2016, **syn. nov.** Type. Madagascar. Prov. Antsiranana: Forêt de Sahafary, S/P de Diego Suarez, 2 Dec 1970, *M. Debray 1554* (holotype: P [P00154364]!).
Croton catatii var. tricholepis Radcl.-Sm., Gen. Croton Madag. Comoro 45. 2016, **syn. nov.** Type. Madagascar. Prov. Toliara: bassin de réception de la Mananara, affluent du Mandrare, entre l’Andohahela et l’Elakelaka, près de Mahamavo, Jan-Feb 1934, *H. Humbert 13876* (holotype: K!; isotypes: P [P00127556!, P00127557!, P00127558]!).
Croton elaeagni var. chrysocarpos Radcl.-Sm., Gen. Croton Madag. Comoro 91. 2016, **syn. nov.** Type. Madagascar. Prov. Toliara: Monte Isalo, in nemore Zombitsy, 800 m, 7 Nov 1967, *L. Bernardi 11281* (holotype: K!; isotypes: G!, P [P00154383]!).
Croton greveanus var. micraster Radcl.-Sm., Gen. Croton Madag. Comoro 41. 2016, **syn. nov.** Type. Madagascar. Prov. Antananarivo: , Tsinjoarivo, 21 Nov 1949, *Service Forestier 1041*-*SF* (holotype: P [P00133806]!).

##### Type.

Madagascar. Prov. Toamasina: Alaotra-Mangoro Region, Didy, 14 Aug 1889, *L.D.M. Catat 1819* (holotype: P [P00131523]!).

##### Habit and distribution.

Shrubs to normally trees; widespread in forested areas of Madagascar (Antananarivo, Antsiranana, Fianarantsoa, Mahajanga, Toamasina, Toliara).

##### Notes.

In this circumscription, *Croton catatii* is a variable but distinctive species. It is usually a tree and typically occurs in montane habitats, but in the drier areas of Isalo and Zombitse area in Fianarantsoa and Toliara Provinces, it can be shrubby, and plants there have more verrucose fruits than elsewhere. Although plants of *C. catatii* are typically finely lepidote, plants such as what was desribed as C. catatii var. setosus from Sahafary in Antsiranana Province can be softly pubescent, with trichomes having long-protruding central rays.

#### 36. Croton chapelieri

Taxon classificationPlantaeMalpighialesEuphorbiaceae

Baill., Adansonia 1: 166. 1861

Oxydectes chapelieri (Baill.) Kuntze, Revis. Gen. Pl. 2: 611. 1891. Type. Based on Croton chapelieri Baill.
Croton louvelii Leandri, Ann. Mus. Colon. Marseille, sér. 5, 7(1): 40. 1939, as ‘*louveli*’. Type. Madagascar. Prov. Toamasina: forêt côtière, , Jan. 1924, *M. Louvel 217* (holotype: P [P00312340]!).
Croton aymoniniorum Leandri, Adansonia, sér. 2, 13: 175. 1973, as ‘*aymoninorum*’. Type. Madagascar. Prov. Toliara: Forêt de Mandena, Fort Dauphin, 19 Oct 1970, *M. Keraudren-Aymonin & G. Aymonin 24940* (holotype: P [P00312374]!).
Croton daphniphylloides Radcl.-Sm., Gen. Croton Madag. Comoro 164. 2016. Type. Madagascar. Prov. Toamasina: Ambila-Lemaitso, 7 Nov 1951, *Service Forestier 4228* (holotype: P [P00133469]!).
Croton daphniphyllus Radcl.-Sm., Gen. Croton Madag. Comoro 161. 2016, as ‘daphniphyllum’. Type. Madagascar. Prov. Toliara: Fort Dauphin in Mandena, 2 km E of Botanic Garden, 24°58'S, 47°00'E, 9 Oct 1990, *D. Faber-Langendoen, N. Dumetz & A. Randrianasolo 2226* (holotype: P [P00133462]!; isotype: MO).
Croton daphniphyllus var. hirsutus Radcl.-Sm., Gen. Croton Madag. Comoro 163. 2016. Type. Madagascar. Prov. Toliara: Préfecture de Tôlañaro (Fort Dauphin), Canton de Mananbaro, Petriky Forest, c. 1.5 km W of large dune near N shore of Lake Andranany, c. 12 km WSW of Tôlañaro (Fort Dauphin), 25°03'S, 46°53'E, 14 Apr 1989, *R. Gereau, R. Rabevohitra & N. Dumetz 3374* (holotype: K!; isotypes: MO [sheet # 3683138], P [P00133120]!).
Croton domohineifolius Radcl.-Sm., Gen. Croton Madag. Comoro 157. 2016, **syn. nov.** Type. Madagascar. Prov. Fianarantsoa: Fanajazana-Mananjary, Jan 1955, *Service Forestier 15451-SF* (holotype: P [P00133471]!).
Croton macrobuxus var. polygynus Radcl.-Sm., Gen. Croton Madag. Comoro 177. 2016, **syn. nov.** Type. Madagascar. Prov. Toliara: near Fort Dauphin, s.d., *J. Cloisel 178* (holotype: P [P00133612]!).
Croton rhododendroides Radcl.-Sm., Gen. Croton Madag. Comoro 163. 2016. Type. Madagascar. Prov. Toliara: Préfecture Tôlañaro (Fort Dauphin), forêt à 5 km de Ste. Luce, au nord de Maliafolaky, 24°47'S, 47°10'E, 21 Oct 1989, *N. Dumetz, G. McPherson & R. Rabevohitra 775* (holotype: P [P00133460]!; isotype: MO!).

##### Type.

Madagascar. sin. loc., s.d., *L.A. Chapelier s.n.* (holotype: P [P00389523]!). Madagascar. Prov. Toliara: Sainte Luce, at entrance to preserve, 24°46'46"S, 47°10'17"E, 10 m, 17 Feb 2009, *B. van Ee, P.E. Berry, B.L. Dorsey & H. Razanatsoa 925* (epitype, designated by Kainulainen et al. 2017a, pg. 37: MICH [MICH1514617]!; additional duplicates: G, K, MAPR, MO, NY, P, TAN).

##### Habit and distribution.

Shrubs; southeastern and eastern coastal Madagascar (Antsiranana, Fianarantosoa, Toamasina, Toliara).

##### Notes.

*Croton chapelieri* was accepted by Leandri (1939) but was restricted to the type specimen, due at least in part to the meager type specimen and the lack of any reported collection locality. Similarly, *C. aymoniniorum* has been recognized only from the type collection. Extensive collections and our own field studies from coastal areas of southeastern Toliara Province (Mandena, Petriky, and Sainte Luce) show that both of these type specimens correspond to a locally common species found in sandy, littoral forests to the west and north of Fort Dauphin, in the Mahabo area of Fianarantosa Province, and then in Toamasina Province much farther north. Chapelier collected mainly in the Foulpointe and Tamatave area (Dorr 1997), and his specimen is similar to the type of *C. louvelii*, from a nearby area. Many herbarium specimens of this species were identified by the late Alan Radcliffe-Smith and subsequent botanists as *Croton daphniphyllus* Radcl.-Sm., or sometimes as *Croton rhododendroides* Radcl.-Sm. for a more pubescent form. See Kainulainen et al. (2017a) for more details about the circumscription of *C. chapelieri*. Newly added to the synonymy here are *C. domohineifolius* Radcl.-Sm. and C. macrobuxus var. polygynus Radcl.-Sm., both from littoral sites on the east coast where *C. chapelieri* is one of the few species to occur.

#### 37. Croton chauvetiae

Taxon classificationPlantaeMalpighialesEuphorbiaceae

Leandri, Adansonia, sér. 2, 10: 310. 1970

##### Type.

Madagascar. Prov. Toliara: P.K. 30, route de Tulear à Tana, 16 Feb 1962, *F. Chauvet 273* (lectotype, designated here: P [P00312370]!; isolectotypes: G [G00074264]!, K [K001044839]!, MO [sheet #04861159]!, P [P00380439]!, P [P00380440]!, P [P00380441]!, TEF [TEF000188]!).

##### Habit and distribution.

Shrubs; southern Madagascar (Toliara).

#### 38. Croton chlaenacicomes

Taxon classificationPlantaeMalpighialesEuphorbiaceae

Leandri, Ann. Mus. Colon. Marseille, sér. 5, 7(1): 50. 1939

Croton arenicola Leandri, Ann. Mus. Colon. Marseille, sér. 5, 7(1): 57. 1939, nom. illeg. non Croton arenicola Small 1905, **syn. nov.** Type. Madagascar. Prov. Toliara: Ambovombe, 10 Aug 1924, *R. Decary 2949* (lectotype, designated by Radcliffe-Smith 2016, pg. 187: K [K001040383]!; isolectotypes: BR [BR0000009424357]!, GB [GB-0047690], G [G00446374]!, P [P00131546]!, P [P04787784]!, S [S07-14591]!, TAN [TAN000523]!). Madagascar. Prov. Toliara: Ambovombe, 1-50 m, 9 Sep 1928, *R. Decary 9182* (syntypes: MO [5598316]!), P [P00131547]!, ibid. loc., 9 Sep 1928, *H. Humbert & C.F. Swingle 5624* (syntype: P [P00418490]!. Madagascar. Prov. Toliara: Ampasimpolaka, 13 Jun 1924, *R. Decary 2872* (syntypes: P [P00133410], MO [1604979]!). Madagascar. Prov. Toliara: environs de Fort-Dauphin, près de Bevilany, 200-300 m, 14 Sep 1928, *H. Humbert & C.F. Swingle 5679* (syntypes: K [K001040350]!, MO [1709286]!, MO [1842163]!, P [P00422331]!, P [P00422332], TAN [TAN000522]); Bevilany, District de Fort-Dauphin, 8 Aug 1932, *R. Decary 10254* (syntypes: MO [1592371]!, P [P00133412]). Madagascar. Prov. Toliara: Manampany, s.d., *M.F. Geay 6385* (syntype: P [P00131548]). Madagascar. Prov. Toliara: moyenne Mananara, a la limite orientale de l’Androy, 27 Nov 1931, *R. Decary 9443* (syntypes: G [G00446375], MO [5598315]!, MO [5608957]!, P [P00133411]! = Croton toliarensis).
Croton leandrii Croizat, Trop. Woods 77: 15. 1944, as ‘leandri’, **syn. nov.** Type. Based on (replacement name for) Croton arenicola Leandri

##### Type.

Madagascar. Prov. Toliara: vallée moyenne du Mandrare, près d’Anadabolava, mont Vohitrotsy, 850 m, Dec 1933, *H. Humbert 12659* (lectotype, designated here: P [P00131549]!; isolectotypes: G [G00018190]!, K [K001040365]!, P [P00131550]!; TAN [TAN000527]!).

##### Habit and distribution.

Shrubs or small trees; southern Madagascar (Toliara).

##### Notes.

Radcliffe-Smith (2016) noted that *Croton leandrii* and *C. chlaenacicomes* are very similar, and in fact there is no reason to keep them separate. Some specimens that were identified under these names in the past, but with pubescence on the upper surface of the leaves as well as below, are now treated under *C. toliarensis* B.W. vanEe & Kainul.

#### 39. Croton chrysodaphne

Taxon classificationPlantaeMalpighialesEuphorbiaceae

Baill., Adansonia 1: 147. 1861

Oxydectes chrysodaphne (Baill.) Kuntze, Revis. Gen. Pl. 2: 611. 1891. Type. Based on Croton chrysodaphne Baill.
Croton lepidotus Aug.DC., Bull. Herb. Boissier, sér. 2, 1: 565. 1901. Type. Madagascar. Prov. Toamasina: Maroantsetra, forêts à l’intérieur de la baie d’Antongil, 1897, *A. Mocquerys 274* (holotype: G-DC [G00018155]!; isotypes: Z [Z-000015984], Z [Z-000015985]).
Croton meeusei Leandri, Notul. Syst. (Paris) 13: 184. 1948, **syn. nov.** Type. Madagascar. Prov. Toamasina: Soanierana-Antasibe, 350 m, 13 Oct 1938, *H.J. Lam & B. Meeuse 5957* (holotype: P [P00312381]!; isotypes: L [L0234889]!, WAG!).
Croton submetallicoides Radcl.-Sm., Gen. Croton Madag. Comoro 58. 2016, **syn. nov.** Type. Madagascar. Prov. Toamasina: Betampona Réserve Naturelle Intégrale, 40 km NW of Toamasina, 275-650 m, 17°31'S, 49°07'E, 28 Sep 1993, *B. Lewis & S. Razafimandimbison 656* (holotype: K!; isotypes: MO!, P [P00433129]!).

##### Type.

Madagascar. Prov. Toamasina: Foulpointe or near Tamatave, 21 Jul 1794 [date on attached letter], *L.A. Chapelier s.n.* (lectotype, designated by Leandri 1972b: P [P00389522]!; isolectotype: P [P00133024]!). Madagascar. sin. loc., s.d., *L.M.A. Dupetit-Thouars s.n.* (syntype: P [P00133022]! = *C. cupreolepis*). Madagascar. Prov. Mahajanga: in montosis in Mazangay, s.d., *W. Bojer s.n.* (syntype: P [P00133021]! = *C. argyrodaphne*); Madagascar. Prov. Toamasina: Analalava Forest Reserve, 7 km W of Foulpointe, 17°42.586'S, 49°27.175'E, 22 m, 2 Mar 2009, *B. van Ee, P.E. Berry & H. Razanatsoa 998* (epitype, designated by Berry et al. 2011, pg. 113: MICH [MICH1514785]!; additional duplicates: G!, MO!, P!, TAN).

##### Habit and distribution.

Large shrubs or trees; eastern lowland Madagascar (Antsiranana, Fianarantsoa, Mahajanga, Toamasina, eastern Toliara).

##### Notes.

Baillon (1861) included material of *Croton argyrodaphne* (*Bojer s.n*.) and *C. cupreolepis* (*Dupetit-Thouars s.n.*), among the syntypes of *C. chrysodaphne*, which was lectotypified on a Chapelier specimen by Leandri (1972b). See Berry et al. (2011) and Berry et al. (2016b) for further discussions.

Both *Croton meeusei* and *C. submetallicoides* fit well within the variation of leaf size and shape of *C. chrysodaphne*, but they are reported to have the normal five petals in staminate flowers, whereas specimens from the Foulpointe area (the presumed lectotype locality as well as the epitype locality) have the unusual number of ten petals. We judge this to be somewhat of an anomaly and not a feature that has been fixed in the species overall. The type of *C. meeusei* lacks pistillate flowers altogether, whereas the type of *C. submetallicoides* has the short, curved inflorescence and pistillate flowers that are typical of *C. chrysodaphne*.

#### 40. Croton chypreae

Taxon classificationPlantaeMalpighialesEuphorbiaceae

Leandri, Adansonia, sér. 2, 9: 498. 1970

##### Type.

Madagascar. Prov. Antsiranana/Mahajanga border: Massif du Tsaratanana, crête séparant les bassins du Sambirano et de la Mahavavy, entre la cote 2362 et la base du piton coté 2831 m, 11-13 Nov 1966, *Service Forestier 27057-SF* (holotype: P [P00312380]!; isotype: TEF [TEF000189]!).

##### Habit and distribution.

Shrubs or small trees; high elevations of north-central Madagascar (border area of Antsiranana and Mahajanga).

#### 41. Croton cotoneaster

Taxon classificationPlantaeMalpighialesEuphorbiaceae

Müll.Arg., Flora 47: 484. 1864

Oxydectes cotoneaster (Müll.Arg.) Kuntze, Revis. Gen. Pl. 2: 611. 1891. Type. Based on Croton cotoneaster Müll.Arg.
Croton mahafaliensis Leandri, Ann. Mus. Colon. Marseille, sér. 5, 7(1): 75. 1939. Type. Madagascar. Prov. Toliara: plateau Mahafaly, Menarandra, Feb 1910, *H. Perrier de la Bâthie 9776* (lectotype, designated here: P [P00312455]!; isolectotype: P [P00133488]!) Madagascar. Prov. Toliara: Beheloka, 80 m, Jun 1910, *H. Perrier de la Bâthie 9767* (syntypes: P [P00312456], P [P00133487]). Madagascar. Prov. Toliara: environs de Tuléar, Aug 1919, *H. Perrier de la Bâthie 12804* (syntype: P [P00312454]). Madagascar. Prov. Toliara: Lac Manampetsa [Tsimanampetsotsa], Apr 1933, *H. Perrier de la Bâthie 19047* (syntype: P [P00312453]).

##### Type.

Madagascar. Prov. Toliara: St. Augustin, 1837, *L. Bouton s.n.* (holotype: K [K001040358]!).

##### Habit and distribution.

Shrubs; southern Madagascar (Toliara).

#### 42. Croton crocodilorum

Taxon classificationPlantaeMalpighialesEuphorbiaceae

Leandri, Ann. Mus. Colon. Marseille, sér. 5, 7(1): 80. 1939

Croton bathianus var. toliarae Radcl.-Sm., Gen. Croton Madag. Comoro 115. 2016, **syn. nov.** Type. Madagascar. Prov. Toliara: Beza Mahafaly Reserve near Betioky, E of Sakamena River, near Ambinda, 23°40'S, 44°38'E, 3 Nov 1987, *P.B. Phillipson 2511* (holotype: K!; isotypes: MO, P [P00127504]!).
Croton crocodilorum var. meridionalis Radcl.-Sm., Gen. Croton Madag. Comoro 112. 2016, **syn. nov.** Type. Madagascar. Prov. Fianarantsoa: Ihosy, in monte Lalanandro, 6 Nov 1967, *L. Bernardi 11259bis* (holotype: K!; isotypes: G!, P [P00154382]!).

##### Type.

Madagascar. Prov. Mahajanga: vallée de la Betsiboka à Marovoay, colline de Marovoay, 50 m, 4–5 Sep 1924, *H. Humbert & H. Perrier de la Bâthie 2347* (lectotype, designated here: G [G00018189]!; isolectotypes: P [P00133026]!, P [P00312451]!, TAN [TAN000528]!). Madagascar. Prov. Mahajanga: Boiny, Nov., *H. Perrier de la Bâthie 9831* (syntypes: P [P00133027]!, P [P00312452]!).

##### Habit and distribution.

Shrubs; mainly southern and western Madagascar (Mahajanga, Toliara), but also in Fianarantsoa Province.

##### Notes.

The Geneva sheet was chosen as the lectotype of *Croton crocodilorum*, since it is the most complete sheet among the syntypes and contains many seeds in the packet. It was annotated by Leandri as ‘*Croton crocodilorum* Leandri n. sp.,’ before it was acquired by Geneva as part of the Herbier Delessert in 1949.

The type of Croton bathianus var. toliarae supposedly differs from the type of *C. crocodilorum* in its rugulose (vs. smooth) seeds (Radcliffe-Smith 2016). However, wrinkled seeds were also seen in the type of *C. crocodilorum*, whereas the type of *C. bathianus* has no fruits. The type of C. crocodilorum var. meridionalis supposedly differs from the type of *C. crocodilorum* by its denser cinereous indumentum on the lower side of the leaves and larger capsules and seeds (Radcliffe-Smith 2016), but we consider this merely an extreme pubescence outlier in the morphological variation of the species.

#### 43. Croton crossolepis

Taxon classificationPlantaeMalpighialesEuphorbiaceae

P.E.Berry & Kainul., Phytotaxa 307(1): 95. 2017

Croton mavoravina var. thysanolepis Radcl.-Sm., Gen. Croton Madag. Comoro 32. 2016. Type. Madagascar. Prov. Toliara: environs de Tuléar, 10-12 Jan 1947, *H. Humbert 19816* (holotype: K!; isotype: P [P00433491]!).

##### Type.

Madagascar. Prov. Toliara: Atsimo-Andrefana Region, along Route Nationale 7 on S side or road, ca. 30 km as the bird flies E of Toliara, 23°19'54"S, 43°55'15"E, 190 m, 8 Feb 2009, *B. van Ee, P.E. Berry, B.L. Dorsey & H. Razanatsoa 823* (holotype: MICH [MICH1210791]!; isotypes: G!, MO!, P!, TAN).

##### Habit and distribution.

Shrubs; dry forest and scrub in southwestern Madagascar (Toliara).

#### 44. Croton cupreolepis

Taxon classificationPlantaeMalpighialesEuphorbiaceae

P.E.Berry, B.W.van Ee & Kainul., Syst. Bot. 41: 977. 2016

Croton nobilis var. delphinensis Leandri, Ann. Mus. Colon. Marseille, sér. 5, 7(1): 48. 1939. Type. Madagascar. Prov. Toliara: Anosy Region, environs de Fort-Dauphin, forêt de Manantantely, 60–300 m, 15 Sep 1928, *H. Humbert & C.F. Swingle 5742* (lectotype, designated by Berry et al. 2016b, pg. 977: P [P00133655]!; isolectotypes: G, [00446398]!, P [P00133656]!).
Croton chrysodaphne var. meridionalis Radcl.-Sm., Gen. Croton Madag. Comoro 62. 2016, **syn. nov.** Type. Madagascar. Prov. Toliara: Préfecture de Fort-Dauphin, Forêt de Manantantely, 50-200 m, 9 Nov 1990, *R. Rabevohitra 2428* (holotype: K!; isotypes: MO!, P [P00133653]!).

##### Type.

Madagascar. Prov. Toliara: Anosy Region, Domaine de la Cascade private preserve (Manantantely-Soanerana), several km north of Route Nationale 13 at Point Kilométrique 9 west of Fort Dauphin, 24°59'05"S, 46°55'39"E, 168–283 m, 2 Aug 2015, *B. van Ee & P.E. Berry 2153* (holotype: MICH!; isotypes: MO, P, TAN!).

##### Habit and distribution.

Trees, eastern montane forests of Madagascar (Antsiranana, Fianarantsoa, Toamasina, Toliara).

#### 45. Croton danguyanus

Taxon classificationPlantaeMalpighialesEuphorbiaceae

Leandri, Ann. Mus. Colon. Marseille, sér. 5, 7(1): 66. 1939, as ‘danguyana’

Croton bracteatus subsp. populifolius Radcl.-Sm., Gen. Croton Madag. Comoro 138. 2016, **syn. nov.** Type. Madagascar. Prov. Mahajanga: Route Tananarive-Majunga, environs de la Betsiboka, Jan 1949, *Service des Eaux et Forêts 148SF* (holotype: P [P00154434]!).

##### Type.

Madagascar. Prov. Mahajanga: environs de Madirovalo (Boïny), Nov 1907, *H. Perrier de la Bâthie 9832* (lectotype, designated here: P [P00389520]!; isolectotype: P [P00327989]!). Madagascar. Prov. Mahajanga: Bongolava, Boïny, Nov 1906, *H. Perrier de la Bâthie 9565* (syntypes: P [P00389519]!, P [P00133040])!). Madagascar. [probably Prov. Mahajanga, Réserve de Namoroka], *Service Forestier 76* (syntype: P [P00389517]!). Madagascar. sin. loc., received 4 Apr 1933, *Service Forestier 104* (syntype: P [P00389518]!).

##### Habit and distribution.

Shrubs; western Madagascar (Mahajanga).

#### 46. Croton decaryi

Taxon classificationPlantaeMalpighialesEuphorbiaceae

Leandri, Bull. Mus. Natl. Hist. Nat., sér. 2, 3: 370. 1931

Croton bevilaniensis Leandri, Ann. Mus. Colon. Marseille, sér. 5, 7(1): 79. 1939, **syn. nov.** Type. Madagascar. Prov. Toliara: confines de l’Anosy et de l’Androy, Bevilany, 8 Aug 1932, *R. Decary 10258* (lectotype, designated here: P [P00127548]!; isolectotypes: K [K000253646]!, TAN [TAN000525]!).

##### Type.

Madagascar. Prov. Toliara: Massif de l’Angavo, à l’Est d’Antanimora, 19 Jul 1926, *R. Decary 4446* (lectotype, designated here: P [P00133044]!; isolectotypes: K [K000253647]!, P [P00133043]!, TAN [TAN000531]!).

##### Habit and distribution.

Shrubs; southern Madagascar (Toliara).

#### 47. Croton dissimilis

Taxon classificationPlantaeMalpighialesEuphorbiaceae

Baill., Bull. Mens. Soc. Linn. Paris 2: 861. 1890

Croton echinatus Radcl.-Sm., Gen. Croton Madag. Comoro 13. 2016, **syn. nov.** Type. Madagascar. Prov. Antsiranana: environs d’Antalaha, 23 Nov. 1948, *H. Humbert & R. Capuron 21918* (holotype: P [P00131511]!).
Croton alaotrensis var. integrifolius Radcl.-Sm., Gen. Croton Madag. Comoro 11. 2016, **syn. nov.** Type. Madagascar. Prov. Toamasina: Mananara Avaratra, Antanambe, above Mahavohobe River, 16°27'S, 49°47'E, 26 Oct 1994, *G. Prance* & *J. Andriantiana 30783* (holotype: K!).

##### Type.

Madagascar. Prov. Toamasina: Fito (Ambanivoules), 1833, *J.P. Goudot 7* (holotype: P [P00154404]!).

##### Habit and distribution.

Shrubs; eastern lowland Madagascar (Antsiranana, Toamasina).

##### Notes.

Leandri (1939) considered *Croton dissimilis* to be a synonym of *C. ambanivoulensis*, but *C. dissimilis* differs in its larger, usually crenate leaves and echinate ovary with trichomes with a long, porrect central ray. Although the type of *C. echinatus* has entire leaves (the type of *C. dissimilis* has crenate leaves), it has similar whitish bark with contrasting tufts of brown trichomes, acropetiolar glands that are cylindrical and shortly stipitate, and the ovary shares the distinctive feature of being covered by long porrect trichomes that give it the appearance of being “echinate.” Similarly, the type of C. alaotrensis var. integrifolius has entire to slightly crenate leaves, and it comes from eastern lowland Toamasina Province, near the presumed type locality of *C. dissimilis*.

#### 48. Croton droguetioides

Taxon classificationPlantaeMalpighialesEuphorbiaceae

Kainul. & Radcl.-Sm., Candollea 71: 331. 2016 [23 Nov 2016]

Croton droguetioides Radcl.-Sm., Gen. Croton Madag. Comoro 16. 2016 [23 Dec 2016]. Type. Madagascar. Prov. Toamasina: marais et bordures de Torotorofotsy, 18°52'S, 48°20'E, 24 Feb 1997, *P. Rakotomalaza et al. 1170* (holotype: K!; isotype: MO!), nom. illeg.
Croton alaotrensis Radcl.-Sm., Gen. Croton Madag. Comoro 10. 2016, **syn. nov.** Type. Madagascar. “Central Madagascar”, received Dec 1883, *R. Baron 3006* (holotype: K!, isotype: P [P00131488]!).
Croton parietarioides Radcl.-Sm., Gen. Croton Madag. Comoro 16. 2016, **syn. nov.** Type. Madagascar. Prov. Toamasina: Ambatoharanana, près Antsevabe, 1000 m, 6 Mar 1951, *G. Cours 4111* (holotype: K!; isotype: P [P00418635]!).

##### Type.

Madagascar. Prov. Toamasina: Alaotra-Mangoro Region, Moramanga District, Andasibe, Berano, Ambatovy mine concession, 18°47'59"S, 48°20'31"E, 1009 m, 22 Mar 2016, *B. van Ee, P. Antilahimena, K. Kainulainen & P.E. Berry 2447* (holotype: MICH [MICH1513195]!, isotypes: MO!, P!, TAN!).

##### Habit and distribution.

Shrubs; eastern montane Madagascar (Toamasina).

##### Notes.

Radcliffe-Smith (2016) erroneously placed the type of *Croton parietarioides* in Antananarivo Province, mistaking Antsevabe for Antsirabe. Antsevabe is close to the Ankeniheny-Zahamena eastern montane forest corridor and lies ca. 25 km southeast of Ambatondrazaka.

#### 49. Croton elaeagni

Taxon classificationPlantaeMalpighialesEuphorbiaceae

Baill., Bull. Mens. Soc. Linn. Paris 2: 848. 1890

Croton elaeagni var. antsingyensis Leandri, Ann. Mus. Colon. Marseille, sér. 5, 7(1): 34. 1939, **syn. nov.** Type. Madagascar. Prov. Mahajanga: Andranoboka, 21 Nov 1932, *J. Leandri 551* (lectotype, designated here: P [P00133092]!; isolectotypes: P [P00133093]!, S [S07-14588]!). Madagascar. Prov. Mahajanga: Amborokontsy, 6 Oct 1932, *J. Leandri 124* (syntypes: P [P00133090]!, P [P00133091]!).
Croton elaeagni var. antsirananae Radcl.-Sm., Gen. Croton Madag. Comoro 90. 2016, **syn. nov.** Type. Madagascar. Prov. Antsiranana: Réserve Spéciale d’Ankarana, à environ 106 km au Sud d’Antsiranana par route, et 12 km à l’Ouest de au Lac Vert, 12°55'13"S, 49°05'10"E, 12 Dec 1995, *O. Andrianantoanina* & *R. Bezara 908* (holotype: K!, isotypes: MO, P [P00433269]!).

##### Type.

Madagascar. Prov. Toliara: Andakabé, près Morondava, s.d., *H. Grevé 82* (lectotype, designated here: P [P00133081]!; isolectotypes: G [G00018188]!, K [K001040385]!, P [P00133082]!, P [P00133083]!, P [P00133084]!, P [P00133085]!, P [P05481486]!).

##### Habit and distribution.

Shrubs; northern, western and southern Madagascar (Antsiranana, Mahajanga, Toliara).

#### 50. Croton elliotianus

Taxon classificationPlantaeMalpighialesEuphorbiaceae

Baill., Bull. Mens. Soc. Linn. Paris 2: 863. 1890

##### Type.

Madagascar. Prov. Toliara: Fort-Dauphin, s.d., *G.F. Scott-Elliot 2970* (lectotype, designated here: P [P00133118]!, isolectotype: K [K000347488]!).

##### Habit and distribution.

Shrubs; southern Madagascar (Toliara).

#### 51. Croton emeliae

Taxon classificationPlantaeMalpighialesEuphorbiaceae

Baill., Adansonia 1: 166. 1861

Croton bifurcatus var. emeliae (Baill.) Müll.Arg. in A.P.de Candolle, Prodr. 15(2): 584. 1866. Type. Based on Croton emeliae Baill.

##### Type.

Mayotte [French Overseas Department]: Jul 1849, *H. Boivin 3381* (lectotype, designated here: P [P00404495]!; isolectotypes: G-DC [G00311983]!, P [P00133119]!, P [P00213565]!).

##### Habit and distribution.

Shrubs; known only from the French island of Mayotte in the Comoro Islands.

#### 52. Croton enigmaticus

Taxon classificationPlantaeMalpighialesEuphorbiaceae

P.E.Berry & B.W.van Ee, Candollea 71: 333. 2016

##### Type.

Madagascar. Prov. Toamasina: Alaotra-Mangoro Region, along dirt road north of Route Nationale 2, past village of Savahoana, 981 m, 18°55'06"S, 48°20'38"E, 14 Aug 2015, *B. van Ee, P.E. Berry & H. Razafindraibe 2212* (holotype: MICH [MICH1513196]!; isotypes: MICH [MICH1513197]!, MO, P, TAN).

##### Habit and distribution.

Shrubs; montane forests of eastern Madagascar (Toamasina).

#### 53. Croton ericius

Taxon classificationPlantaeMalpighialesEuphorbiaceae

Leandri, Cat. Pl. Madag., Euphorb.: 32. 1935

Croton horridulus Baill., Bull. Mens. Soc. Linn. Paris 2: 977. 1891, nom. illeg. non Croton horridulus (Baill.) Müll.Arg., 1866.
Croton lapiazicola Leandri, Ann. Mus. Colon. Marseille, sér. 5, 7(1): 42. 1939, **syn. nov.** Type. Madagascar. Prov. Mahajanga: Tsingy du Bemaraha (9^e^ Réserve), Feb. 1933, *J. Leandri 945* (lectotype, designated here: P [P00312338]!; isolectotype: P [P00133400]!). Madagascar. Prov. Mahajanga: Tsingy du Bemaraha (9^e^ Réserve), 5 Oct 1932, *J. Leandri 139* (syntype: P [P00312339]!), ibid. loc., Oct 1932, *J. Leandri 162* (syntype: P [P00389500]!).

##### Type.

Madagascar. *R. Baron 5579* (holotype: P [P00133163]!); isotype: K [K000422591]!.

##### Habit and distribution.

Shrubs; northern and western Madagascar (Antsiranana, Mahajanga).

#### 54. Croton farinosus

Taxon classificationPlantaeMalpighialesEuphorbiaceae

Lam., Encycl. 2: 211. 1786

Oxydectes farinosa (Lam.) Kuntze, Revis. Gen. Pl. 2: 611. 1891. Type. Based on Croton farinosus Lam.
Croton scottii Baill., Bull. Mens. Soc. Linn. Paris 2: 967. 1891, as ‘*scotti*’, **syn. nov.** Type. Madagascar. Prov. Toliara: Fort-Dauphin, s.d., *G.F. Scott-Elliot 2987* (holotype: P [P00154436]!; isotype: K [K000253644]!).
Croton moraharivensis Leandri, Ann. Mus. Colon. Marseille, sér. 5, 7(1): 68. 1939, **syn. nov.** Type. Madagascar. Prov. Toliara: vallée de la Manambolo, rive droite (bassin du Mandrare) aux environs d’Isomono (confluent de la Sakamalio), Mont Morahariva, 1000-1400 m, Dec 1933, *H. Humbert 13176* (lectotype, designated here: P [P00132989]!; isolectotypes: G [G00018151]!, P [P00132990]!). Madagascar. Prov. Toliara: bassin de réception de la Mananara, affluent du Mandrare, pentes occidentales des montagnes entre l’Andohahela et l’Elakelaka, 600-800m, Feb 1934, *H. Humbert 14041* (syntypes: K [K000815882]!, P [P00132991]!).
Croton vohibariensis Leandri, Ann. Mus. Colon. Marseille, sér. 5, 7(1): 78. 1939, **syn. nov.** Type. Madagascar. Prov. Toliara: vallée moyenne du Mandrare, près d’Anadabolava, Mont Vohibaria (sommet), 800-810 m, Dec 1933, *H. Humbert 12627* (lectotype, designated here: P [P00133322]!; isolectotypes: G [G00446404]!, K [K000253645]!, P [P00133323]!, P [P00133324]!). Madagascar. Prov. Toliara: vallée moyenne du Mandrare, près d’Anadabolava, Mont Vohitrotsy, sommet, vers 850 m, Dec 1933, *H. Humbert 12673* (syntype: P [P00133325]!).

##### Type.

Madagascar: sin. loc., s.d., *P. Commerson s.n.* (holotype: P-LA [P00382067]!; isotypes: G [G00446383]!, G [G00446384]!, LINN [LINN-HS1492-5], P [P00404490]!).

##### Habit and distribution.

Shrubs; southeastern Madagascar (Toliara).

##### Notes.

Although there is a wide elevational variation in this species as here circumscribed, the three names listed in synonymy share the characteristic glaucous-farinose lower leaf surface, and all appear to have an affinity for igneous outcroppings.

#### 55. Croton ferricretus

Taxon classificationPlantaeMalpighialesEuphorbiaceae

Kainul., B.W.van Ee & P.E.Berry, Candollea 71: 337. 2016 [23 Nov 2016]

Croton lepidotoides Radcl.-Sm., Gen. Croton Madag. Comoro 71. 2016 [23 Dec 2016], **syn. nov.** Type. Madagascar. Prov. Toamasina: ca. 15 air-km NE of Moramanga, ca. 11 km E of Antanambao, Ambatovy, 18°51'08"S, 48°18'40"E, 30 Jan 1997, *P. Rakotomalaza et al. 1024* (holotype: K!; isotype: MO!).

##### Type.

Madagascar. Prov. Toamasina: Alaotra-Mangoro Region, Moramanga district, Andasibe, Berano, Ambatovy mine concession, on “cuirasse” between the workers houses and the Ambatovy supply road, 18°51'02"S, 48°18'29"E, 1142 m, 21 Mar 2016, *B. van Ee, P. Antilahimena, K. Kainulainen & P.E. Berry 2436* (holotype MICH [MICH1513194]!; isotypes: G!, K!, MAPR!, MO!, P!, TAN!).

##### Habit and distribution.

Shrubs; eastern Madagascar (Moramanga District of Toamasina Province).

#### 56. Croton fianarantsoae

Taxon classificationPlantaeMalpighialesEuphorbiaceae

Leandri, Adansonia, sér. 2, 13: 295. 1973

Croton nitidulus var. grandifolius Leandri, Ann. Mus. Colon. Marseille, sér. 5, 7(1): 39. 1939, as ‘*grandifolia*’. Type. Madagascar. Prov. Fianarantsoa: Vondrozo, 17 Sep 1926, *R. Decary 5333* (holotype: P [P00133643]!; isotypes: K [K001040382]!, P [P00425637 - wood], S [S07-17118]!, TAN [TAN000539]!).
Croton nitidulus var. tandrokensis Leandri, Ann. Mus. Colon. Marseille, sér. 5, 7(1): 39. 1939, **syn. nov.** Type. Madagascar. Prov. Fianarantsoa: Massif d’Andringitra, col du Tandroka, versant Est, 1200 m, Sep 1910, *H. Perrier de la Bâthie 9749* (holotype: P [P00133638]!).
Croton fianarantsoae var. grandifolius Leandri, Adansonia, sér. 2, 13: 297. 1973, as ‘*grandifolia*’, **syn. nov**. Type. Madagascar. Prov. Toamasina: Sahasanato, Antajonomby, Canton Befody, Distr. Nosy-Varika [N de Manajary], 21 May 1955, *Service Forestier 14556* (lectotype, designated here: P [P00154399]!; isolectotypes: P [P00154400]!, P [P00154401]!, TEF [TEF000191]!).
Croton daphniphylloides var. hirsutus Radcl.-Sm., Gen. Croton Madag. Comoro 165. 2016, **syn. nov.** Type. Madagascar. Prov. Fianarantsoa: Parc National de Ranomafana, Talakately, 21°15'S, 47°27'E, 27 Jul–6 Aug 1993, *A. Kotozafy 131* (holotype: K!; isotypes: MO, P [P00433137]!).
Croton fianarantsoae var. coursii Radcl.-Sm., Gen. Croton Madag. Comoro 154. 2016, **syn. nov.** Type. Madagascar. Prov. Toamasina: Rahobevava, 1300 m, 14 Mar 1951, *G. Cours 4370* (holotype: P [P00133614]!).
Croton fianarantsoae var. microphyllus Radcl.-Sm., Gen. Croton Madag. Comoro 155. 2016, **syn. nov.** Type. Madagascar. Prov. Fianarantsoa: Parc National de Ranomafana, SE of Savondronona, Maharira, 21°18'S, 47°23'E, 1200-1400 m, 21-23 Apr 1993, *S. Malcomber, C. Hemingway & A. Randriamanantena 2441* (holotype: K!; isotypes: MO, P [P00422457]!).
Croton fianarantsoae var. ranomafanae Radcl.-Sm., Gen. Croton Madag. Comoro 156. 2016, **syn. nov.** Type. Madagascar. Prov. Fianarantsoa: Parc National de Ranomafana, trail S from Cabine de Recherche to Vato camp, 21°15'S, 47°27'E, 1060 m, 11-15 Nov 1991, *S. Malcomber, A. Leeuwenberg, C. Rakotomazana, H. Ranarljadna & G. Rahajasoa 1042* (holotype: K; isotypes: MO, P [P00422461]!).
Croton fianarantsoae var. tandrokensis (Leandri) Radcl.-Sm., Gen. Croton Madag. Comoro 156. 2016, **syn. nov.** Type. Based on Croton nitidulus var. tandrokensis Leandri

##### Type.

Madagascar. Prov. Fianarantsoa: Andrambovato, Parcelle B5 [Fort Carnot], 23 Mar 1954, *Service Forestier 10169-SF* (holotype: P [P00338570]!; isotype: P [P00154402]!, TEF [TEF000190]!).

##### Habit and distribution.

Shrubs to small trees; eastern Madagascar (Antsiranana, Fianarantsoa, Toamasina).

##### Notes.

*Croton fianarantsoae* is part of a difficult species complex, and further work is needed to differentiate it from similar species such as *C. nitidulus*.

#### 57. Croton fothergillifolius

Taxon classificationPlantaeMalpighialesEuphorbiaceae

Baill., Adansonia 1: 150. 1861, as ‘fothergillifolium’

Oxydectes fothergillifolia (Baill.) Kuntze, Revis. Gen. Pl. 2: 611. 1891. Type. Based on Croton fothergillifolius Baill.

##### Type.

Mauritius. sin. loc., *L.M.A. Du Petit-Thouars s.n.* (lectotype, designated here: P [P00404184]!; isolectotype: P [P00404183]!).

##### Habit and distribution.

Shrubs; Mauritius.

##### Notes.

Bojer (1837) listed a plant from the Grand Bassin of Mauritius as *Croton muricatus* Vahl, a native Malagasy species. Based on this, the name “*C. muricatus* Bojer, nom. nud.” has been included in several indices, including IPNI, Tropicos, and Govaerts et al. (2000). In the same manner as Leandri (1939), we interpret Bojer (1837) as a misidentification of what Baillon (1861) later described as *C. fothergillifolius*, rather than the publication of a nomen nudum.

#### 58. Croton geayi

Taxon classificationPlantaeMalpighialesEuphorbiaceae

Leandri, Bull. Mus. Natl. Hist. Nat., sér. 2, 3: 368. 1931

Croton geayi var. paucisquamatus Radcl.-Sm., Gen. Croton Madag. Comoro 94. 2016, **syn. nov.** Type. Madagascar. Prov. Toliara: Plateau Mahafaly à l’Ouest de Betioky, 17-20 Mar 1955, *H. Humbert* & *R. Capuron 29490* (holotype: P [P00123679]!).
Croton geayi var. pubescens Radcl.-Sm., Gen. Croton Madag. Comoro 95. 2016, **syn. nov.** Type. Madagascar. Prov. Toliara: vallée inférieure de l’Onilahy, 12 Aug 1928, *H. Humbert* & *C.F. Swingle 5235* (holotype: P [P00133137]!; isotype: G!).

##### Type.

Madagascar. Prov. Toliara: sin. loc., 1906, *M.F. Geay 28* (holotype: P [P00133132]!).

##### Habit and distribution.

Shrubs; southern Madagascar (Toliara).

##### Notes.

The two varieties of *Croton geayi* described by Radcliffe-Smith (2016) only differ from the nominal species in degree and type of pubescence, and this does not seem to justify their recognition as distinct taxonomic entities.

#### 59. Croton glomeratus

Taxon classificationPlantaeMalpighialesEuphorbiaceae

Aug.DC., Bull. Herb. Boissier, sér. 2, 1: 566. 1901

Croton lamianus Leandri, Notul. Syst. (Paris) 13: 184. 1948, as ‘Lamiana’, **syn. nov.** Type. Madagascar. Prov. Toamasina: Soanierana-Ambahoabe, 50 m, 5 Dec 1938, *H.J. Lam & B. Meeuse 5756* (lectotype, designated here: P [P00312383]!; isolectotypes: K [K001040375]!, L [93970419], P [P00312382]!). Madagascar. Prov. Toamasina: Soanierana-Ambahoabe, 100 m, 3 Dec 1938, *H.J. Lam & B. Meeuse 5604* (syntypes: P [P00133398]!, L [939171491]).
Croton fianarantsoae var. masoalae Radcl.-Sm., Gen. Croton Madag. Comoro 154. 2016, **syn. nov.** Type. Madagascar. Prov. Antsiranana: Manarivola, Sahamalaza, Vinanivao, Antalaha, Parc Masoala, 15°48'25"S, 50°17'15"E, 12–22 Feb 1996, *R. Bernard et al. 213* (holotype: K!; isotypes: MO, P [P00433259]!).

##### Type.

Madagascar. Prov. Toamasina: Maroa [Maroantsetra], 1898, *A. Mocquerys 298* (lectotype, designated here: G [G00446385]!). Madagascar. Prov. Toamasina: Maroa [Maroantsetra], s.d., *A. Mocquerys 317* (syntype: G [G00446386]!).

##### Habit and distribution.

Shrubs; northeastern Madagascar (Antsiranana, Toamasina).

#### 60. Croton goudotii

Taxon classificationPlantaeMalpighialesEuphorbiaceae

Baill., Adansonia 1: 157. 1861

Croton platanifolius Bojer ex Baker, J. Bot. 20: 268. 1882. Type. Madagascar. edges of woods in East Betsileo, received Jul 1880, *R. Baron 262* (holotype: K [K000422589]!; isotype(fragment): P [P00133700, upper packet]!).
Croton emirnensis Baker, J. Linn. Soc., Bot. 20: 252. 1883. Type. Madagascar. sin. loc. s.d., *R. Baron 1854* (lectotype, designated here: K [K000422588]!). Madagascar. sin. loc., s.d., *R. Baron 1841* (syntypes: K [K001040356]!, P [P00133701]!).
Oxydectes goudotii (Baill.) Kuntze, Revis. Gen. Pl. 2: 611. 1891. Type. Based on Croton goudotii Baill.
Croton mollivelus Baill. Bull. Mens. Soc. Linn. Paris 2: 926. 1891, as ‘*mollivelum*’. Type. Madagascar. sin. loc., s.d., *C.M. Le Myre de Vilers s.n.* (holotype: P [P00310180]!).
Croton tsaratananae Leandri, Ann. Mus. Colon. Marseille, sér. 5, 7(1): 74. 1939, **syn. nov.** Type. Madagascar. Prov. Antsiranana/Mahajanga border: Mont Tsaratanana, 2000 m, Dec 1912, *H. Perrier de la Bâthie 9720* (lectotype, designated here: P [P00389496]!; isolectotypes: P [P00154411]!, P [P00154412]!).
Croton goudotii var. tsaratananae (Leandri) Radcl.-Sm., Gen. Croton Madag. Comoro 128. 2016, **syn. nov.** Type. Based on Croton tsaratananae Leandri

##### Type.

Madagascar. Prov. Antananarivo: environs de Tananarivo, rec’d. 1840, *J.P. Goudot s.n.* (holotype: G [G00018187]!; isotype (fragment from G): P [P00133700, lower packet]!

##### Habit and distribution.

Trees; upland forests across Madagascar (Antananarivo, Antsiranana, Fianarantsoa, Mahajanga, Toamasina, Toliara).

#### 61. Croton gracilior

Taxon classificationPlantaeMalpighialesEuphorbiaceae

Radcl.-Sm., Gen. Croton Madag. Comoro 108. 2016

##### Type.

Madagascar. Prov. Mahajanga: Tsingy de Bemaraha, N of the Malambo River, 19°09'S, 44°49'E, 27 Nov 1996, *C.C.H. Jongkind, J. Andriantiana* & *H. Razanatsoa 3235* (holotype: K!; isotypes: MO!, WAG).

##### Habit and distribution.

Shrubs; western Madagascar (Mahajanga – Melaky Region).

#### 62. Croton grangerioides

Taxon classificationPlantaeMalpighialesEuphorbiaceae

Bojer ex Baill., Adansonia 1: 149. 1861

Croton boutonianus Müll.Arg., Linnaea 34: 80. 1865. Type. Mauritius. in sylvis montosis, 1833, *W. Bojer s.n*. (lectotype, designated here: G-DC [G00311198]!; isolectotypes: G [G00446409]!, M [M0110371]!). Mauritius. 1857, *L. Bouton s.n.* (syntype: G-DC [G00311197]!).
Oxydectes boutoniana (Müll.Arg.) Kuntze, Revis. Gen. Pl. 2: 611. 1891. Type. Based on Croton boutonianus Müll.Arg.
Oxydectes grangerioides (Bojer ex Baill.) Kuntze, Revis. Gen. Pl. 2: 610. 1891. Type. Based on Croton grangerioides Bojer ex Baill.

##### Type.

Mauritius. s.d., *J. Néraud s.n.* (lectotype, designated here: G [G00446387]!; possible isolectotype: P [P00404205]!). Mauritius. à l’embrasure, au-dessous du Pouce, s.d. , *L.M.A. Du Petit-Thouars s.n.* (syntypes: P [P00404204]!, P [P0040179]!). Mauritius. crêtes de la montagne de Port-Louis, vers l’extrémité au-dessus de l’anse Courtois, Oct 1849, *L.H. Boivin s.n.* (syntype: P [P00404203]!). Mauritius. 1850, *J.N.E. Vesco s.n.* (syntype: P [P00404180]!). Mauritius. *W. Bojer s.n.* (original material: MAU [1402; specimen not seen], possible type: G [G00446413]!, M [M0110457]!).

##### Habit and distribution.

Shrubs; Mauritius.

##### Notes.

Baker (1877) distinguished *Croton boutonianus* from *C. grangerioides* in his key by the former having entire leaves and pistillate flowers with petals and the latter obscurely crenulate leaves and pistillate flowers that lack petals. Leandri (1939) accepted *C. grangerioides*, but appears to have overlooked mentioning *C. boutonianus*, even as a synonym. Coode (1982) treated *C. boutonianus* as a synonym of *C. grangerioides*, describing the petals of the staminate flowers as small and delicate, and the crenulation of the leaf margins as variable. We follow here the taxonomy of Coode (1982) in treating *C. boutonianus* as a synonym of *C. grangerioides*.

#### 63. Croton greveanus

Taxon classificationPlantaeMalpighialesEuphorbiaceae

Baill., Bull. Mens. Soc. Linn. Paris 2: 849. 1890

Croton greveanus var. borealis Leandri, Ann. Mus. Colon. Marseille, sér. 5, 7(1): 45. 1939. Type. Madagascar. Prov. Mahajanga: région littorale, entre Masoarivo et Ambato, Nov 1932, *J. Leandri 536* (lectotype, designated here: P [P00133803]!; isolectotypes: G [G00446389]!, P [P00133804]!, P [P00133805]!). Madagascar. Prov. Mahajanga: Tsingy du Bemara, 9^e^ Réserve Naturelle, Tsiombivositra, 10 Nov 1932, *J. Leandri 517* (syntype: P [P00133802]!). Madagascar. Prov. Mahajanga: Trano Passage, *J. Leandri 264* (P [P00133800], P [P00133802]!). Madagascar. Prov. Mahajanga: Savalika, 11 Dec 1932, *J. Leandri 341* (P [P00133785]!, P [syntype: P00133786]!). Madagascar. Prov. Mahajanga: , Firingalava, Dec 1898, *H. Perrier de la Bâthie 777* (syntypes: P [P00133788]!, P [P00133789]!). Madagascar. Prov. Mahajanga: causse d’Ankara, bois rocailleux calcaire de Kamakama, Dec 1901, *H. Perrier de la Bâthie 9798* (syntype: P [P00154464]!).
Croton antanosiensis var. pubescens Radcl.-Sm., Gen. Croton Madag. Comoro 43. 2016, **syn. nov.** Type. Madagascar. Prov. Toliara: 44 km Tulear-Ihosy, 12 Nov 1967, *L. Bernardi 11411* (lectotype, designated here: G [“Hb. G 0044814”]!; isolectotypes: G [“Hb. G 0044804”]!, P [P00154375]!).
Croton boinensis var. parcelepidotus Radcl.-Sm., Gen. Croton Madag. Comoro 78. 2016, **syn. nov.** Type. Madagascar. Prov. Mahajanga: Ampasimandroro, Maintirano, 12 May 1956, *Service Forestier 16322-SF* (lectotype, designated here: P [P00347747]!; isolectotype: P [P00154435]!).

##### Type.

Madagascar. Prov. Toliara: Bé-Kapaké [Bekopaka], ad. riv. Morondava, s.d., *H. Grevé 239* (lectotype, designated here: P [P00404488]!; isolectotypes: P [P00133768]!, P [P00133769]!, P [P00133770]!, P [P00133771]!).

##### Habit and distribution.

Large shrubs or small trees; western, central, and southern Madagascar (Fianarantsoa, Mahajanga, Toliara).

#### 64. Croton guerelae

Taxon classificationPlantaeMalpighialesEuphorbiaceae

Leandri, Adansonia, sér. 2, 9: 507. 1969 [1970]

##### Type.

Madagascar. Prov. Antananarivo: forêt basse a feuilles persistantes, restes de forêt du Mt. Ambohiby (SE de Tsiroanomandidy), 1600 m, 11-16 Nov 1952, *J. Leandri et al. 1790* (lectotype, designated here: P [P00312376]!; isolectotypes: K [K001044845]!, P [P00404478]!, P [P00404479]!).

##### Habit and distribution.

Shrubs; central Madagascar (Antananarivo, Toamasina).

#### 65. Croton heteranthus

Taxon classificationPlantaeMalpighialesEuphorbiaceae

Aug.DC., Bull. Herb. Boissier, sér. 2, 1: 566. 1901

Croton ivohibensis var. furfuraceus Radcl.-Sm., Gen. Croton Madag. Comoro 149. 2016, **syn. nov.** Type. Madagascar. Prov. Antsiranana: Sous-Préfecture d’Andapa, Bealampona, Befingotra, Réserve Anjanaribe-Sud on Rn Andapa-Bealanana, Antsahanifelana, near Ampiferantany, 14°47'45"S, 49°27'54"E, 22 May 1995, *D. Ravelonarivo* & *R. Rabesonina 816* (holotype: K!; isotype: MO!).
Croton scorpistogyne Radcl.-Sm., Gen. Croton Madag. Comoro 166. 2016, **syn. nov.** Type. Madagascar. Prov. Antsiranana: Ambohihalanana Ct., Antalaha Distr., 15 Dec 1956, Réserves Naturelles 8059-Rn (holotype: P [P00133301]!).

##### Type.

Madagascar. Prov. Toamasina: Maroa [Maroantsetra], forêts à l’intérieur de la baie d’Antongil, 1897, *A. Mocquerys 228* (holotype: G [G00018162]!; isotype: Z [Z-000015980]).

##### Habit and distribution.

Large shrubs or small trees; northeastern Madagascar (Antsiranana, Toamasina).

#### 66. Croton hildebrandtii

Taxon classificationPlantaeMalpighialesEuphorbiaceae

Baill., Bull. Mens. Soc. Linn. Paris 2: 847. 1890

Croton heterochrous Baill., Bull. Mens. Soc. Linn. Paris 2: 862. 1890, nom. illeg. non Croton heterochrous Müll.Arg. 1865.
Croton belintae Leandri, Cat. Pl. Madag., Euphorb.: 30. 1935, **syn. nov.** Type. Madagascar. Prov. Antsiranana: Vavatobé, Belinta, Feb 1880, *J.M. Hildebrandt 3326* (lectotype, designated here: P [P00127506]!; isolectotypes: G [G00018184]!, G [G00018185]!, JE [JE00015890], JE [JE00015891]!, K [K001040370]!, M [M-0110366], P [P00127507]!, P [P00133155]!, P [P00133156]!).

##### Type.

Madagascar. Prov. Antsiranana: Pasandava-bai [Bay], Kisimani, Jun 1879, *J.M. Hildebrandt 3013* (lectotype, designated here: G [G00075617]!; isolectotypes: G [G00075618]!, G [G00018186]!, JE [JE00000063]!, K [K000347497]!, M [M0110367]!), P [P00133157]!, P [P00133158]!, P [P00133159]!).

##### Habit and distribution.

Shrubs; northern and northwestern Madagascar (Antsiranana, Mahajanga).

##### Notes.

The type of *Croton belintae* differs from the typically lepidote plants of *C. hildebrandtii* only in the presence of prominent porrect rays emerging from the center of the lepidote scales, which gives the plant a more fuzzy-pubescent appearance. In all other characters, however, such as leaf shape, petiolar glands, and the small flowers with somewhat recurved pedicels, they are identical. We therefore treat *C. belintae* as a synonym of the earlier name *C. hildebrandtii*.

#### 67. Croton hovarum

Taxon classificationPlantaeMalpighialesEuphorbiaceae

Leandri, Ann. Mus. Colon. Marseille, sér. 5, 7(1): 40. 1939

Croton rubricapitirupis Leandri, Adansonia, sér. 2, 13: 173. 1973, **syn. nov.** Type. Madagascar. Prov. Fianarantsoa: Ambatomenaloha, à l’W d’Itremo, 20 Jan 1955, *R. Capuron 11580* (lectotype, designated here:P [P00380442]!; isolectotypes: P [P00154295]!, P [P00154296]!, P [P00154297]!, TEF [TEF000185]).
Croton anisatus var. hirsutus Radcl.-Sm., Gen. Croton Madag. Comoro 14. 2016, **syn. nov.** Type. Madagascar. Prov. Toamasina: Ambatondrazaka Distr., Ambatosoratra Canton, 26 Aug 1959, *Réserves Naturelles 10857* (holotype: P [P00133189]!).
Croton cassinoides var. alaotrensis Radcl.-Sm., Gen. Croton Madag. Comoro 10. 2016, **syn. nov.** Type. Madagascar. Prov. Toamasina: Ambatondrazaka Distr., Ambatosoratra Canton, near the shore of Lac Alaotra, 16 Oct 1958, *Réserves Naturelles 9633*-Rn (holotype: P [P00131518]!).
Croton greveanus var. ambositrensis Leandri ex Radcl.-Sm., Gen. Croton Madag. Comoro 39. 2016, **syn. nov.** Type. Madagascar. Prov. Fianarantsoa: Andina, environs d’ Ambositra, Dec 1921, *H. Perrier de la Bâthie 18606* (holotype: P [P00133790]!).
Croton hovarum var. lepidotus Radcl.-Sm., Gen. Croton Madag. Comoro 179. 2016, **syn. nov.** Type. Madagascar. sin. loc., Oct 1881, *R. Baron 678* (holotype: K [not seen]; isotype: P [P00133165]!).
Croton hovarum var. subglaber Radcl.-Sm., Gen. Croton Madag. Comoro 179. 2016, **syn. nov.** Type. Madagascar. Prov. Fianarantsoa: Rn 5, Ambalavao Distr., Sendrisoa Canton, 12 Nov 1953, *Réserves Naturelles 5871* (lectotype, designated here: P [P00133188]!; isolectotype: P [P00133187]!).
Croton ivohibensis Leandri, Ann. Mus. Colon. Marseille, sér. 5, 7(1): 37. 1939, **syn. nov.** Type. Madagascar. Prov. Fianarantsoa: chaine du Vohibory, à l’ouest d’Ivohibe, 1000-1300 m, 1 Nov 1924, *H. Humbert 3051* (lectotype, designated here: P [P00133264]!; isolectotypes: G [G00018157]!, K [K001040387]!, P [P00133263]!, P [P00133265]!, TAN [TAN000535]!).
Croton ivohibensis var. lepidotus Radcl.-Sm., Gen. Croton Madag. Comoro 149. 2016, **syn. nov.** Type. Madagascar. Prov. Fianarantsoa: Réserve Naturelle V, Antaniloha Canton, Ivohibe District, 29 Nov 1951, Réserves Naturelles 35-29 Rn (lectotype, designated here: P [P00133632]!; isolectotype: P00133631]!).

##### Type.

Madagascar. Prov. Antananarivo: Imerina, Ifanangoavana, Jan 1881, *J.M. Hildebrandt 3811* (lectotype, designated here: P [P00133177]!; isolectotypes: G [G00446390]!, G [G00446405]!, JE [JE00015925]!, K [K001040345]!, M [M0110359]!), P [P00133176]!). Madagascar. sin. loc., s.d., *R. Baron 675* (syntype: P [P00133164]!), *Baron 678* (syntypes: K [K001040346]!, P [P00133165]), *R. Baron 3151* (syntypes: K [K001040347]!, P [P00133166], P [P00133167]!). Madagascar. Prov. Antananarivo: 20 km E de Tananarive, sur la route de Tamatave, 30 Oct 1928, *R. Decary 6813* (syntypes: P [P00133171]!, G [G00018161]!, S [S07-14579]!), ibid. loc.. 30 Sep 1928, *R. Decary 6834* (syntypes: P [P00133172]!; MO [5598313]!). Madagascar. Prov. Antananarivo: au Nord d’Ankazobe, 9 Mar 1930, *Decary 7292* (syntypes: P [P00133173], GB [GB-0047692]!), ibid. loc., 11 Mar 1930, *R. Decary 7380* (syntypes: P [P00133174], MO [1602793]!). Madagascar. Prov. Antananarivo: Manankazo, au NE d’Ankazobe, Nov 1913, *H. Perrier de la Bâthie 9867* (syntypes: P [P0013383]!, P [P0013384]!, P [P0013385]!), ibid. loc., *H. Perrier de la Bâthie 9877* (P [P00133186]!). Madagascar. Prov. Toamasina: Andovoranto, Moramanga, bord de la Sahamarirana entre Ampasimpotsy et Bevalanirano, 24 Oct 1912, *R. Viguier & H. Humbert 989* (syntypes: P [P00422391]!, P [P00422392]!, K [K001040344]!). Madagascar. Prov. Fianarantsoa: Vakinankaratra, Ambatolampy, bois entre Tsinjoarivo et , 2 Oct 1912, *R. Viguier & H. Humbert 1924* (syntypes: P [P00133190]!, P [P00133191]!).

##### Habit and distribution.

Shrubs; central upland Madagascar (Antananarivo, Fianarantsoa, Toamasina).

##### Notes.

Leandri (1973a) distinguished *Croton rubricapitirupis* from *C. hovarum* almost exclusively by the sparser, lepidote pubescence on the leaf undersides of *C. rubricapitirupis*. *Croton hovarum* is quite variable in leaf size and degree of indumentum, and we do not consider this a sufficient distinction at the species level. In its monopodial branching, large accrescent female calyx, and finely crenate to serrate leaf margins, *C. hovarum* is a readily recognizable species in upland Madagascar. The syntype *Viguier & Humbert 989* corresponds to *C. hypochalibaeus*.

#### 68. Croton humbertii

Taxon classificationPlantaeMalpighialesEuphorbiaceae

Leandri, Ann. Mus. Colon. Marseille, sér. 5, 7(1): 22. 1939, as ‘humberti’

Croton ivohibensis var. alaotrensis Radcl.-Sm., Gen. Croton Madag. Comoro 148. 2016, **syn. nov.** Type. Madagascar. Prov. Toamasina: Befody – forêt de l’Est, Ambatondrazaka, 29 Aug 1952, *Service de Eaux et de Forêts de Madagascar 4052-SF* (holotype: P [P00133207]!).

##### Type.

Madagascar. Prov. Toamasina: forêt d’Analamazaotra, Dec 1932, *J. Leandri 709* (lectotype, designated here: P [P00133198]!; isolectotypes: P [P00133197]!, P [P00133199]). Madagascar. Prov. Toamasina: forêt d’Analamazaotra, 1000m, 19 Oct 1912, *R. Viguier & H. Humbert 805* (syntypes: P [P00133209]!, P [P00133210]!, B [B100153963]!), ibid. loc., 21 Oct 1912, *R. Viguier & H. Humbert 830* (G [G00018160]!, P [P00133210]!, P [P00133211]!, P [P00133212]), ibid. loc., Feb 1912, *H. Perrier de la Bâthie 9739* (syntypes: P [P00133200]!, P [P00133201]!, P [P00133202]!).

##### Habit and distribution.

Shrubs; eastern montane Madagascar (Toamasina).

#### 69. Croton humblotii

Taxon classificationPlantaeMalpighialesEuphorbiaceae

Baill., Bull. Mens. Soc. Linn. Paris 2: 846. 1890

Croton humblotii var. anjuanensis Leandri, Ann. Mus. Colon. Marseille, sér. 5, 7(1): 44. 1939, **syn. nov.** Type. Union of the Comoros: Anjouan, Apr 1905, *Lavanchie s.n.* (holotype: P [P00154408]!).

##### Type.

Mayotte [French Overseas Department]: forêt de Combani, 10 Oct 1884, *L. Humblot 1298* (lectotype, designated here: P [P00196068]!; isolectotypes: K [K000347496]!, K [K001040343]!, LD [LD1210694], LD [LD1210214], LG [LG000009002795]), P [P00196069], P [P00196070].

##### Habit and distribution.

Shrubs or trees; Comoro Islands, occurring on Mayotte and the three islands of the Union of the Comoros (Anjouan, Grande Comore, and Mohéli).

#### 70. Croton hypochalibaeus

Taxon classificationPlantaeMalpighialesEuphorbiaceae

Baill., Bull. Mens. Soc. Linn. Paris 2: 862. 1890, as ‘hypochalibaeum’

Croton squamiger var. acutifolius Müll.Arg. in A.P.de Candolle, Prodr. 15(2): 523. 1866, **syn. nov.** Type. Madagascar. in sylvis ins. Madag., s.d., *W. Bojer s.n.* (holotype: P [P00133274]!, isotype: M [M0110356]!).
Croton alceicornu Radcl.-Sm., Gen. Croton Madag. Comoro 70. 2016, **syn. nov.** Type. Madagascar. Prov. Toamasina: Ambatovy, 18°51'34"S, 48°18'25"E, 3 Mar 1997, *P. Rakotomalaza et al. 1220* (holotype: K!; isotype: MO!).
Croton antanosiensis var. fianarantsoae Radcl.-Sm., Gen. Croton Madag. Comoro 42. 2016, **syn. nov.** Type. Madagascar. Prov. Fianarantsoa: Ranomafana National Park, 7 km S of the National Road 25 W of Ranomafana, 21°15'30"S, 47°25'00"E, 31 Mar 1993, *D. Turk et al. 378* (holotype: K!; isotype: G [G00414720]!, MO!, P [P00418629]!).
Croton oligostemon Radcl.-Sm., Gen. Croton Madag. Comoro 45. 2016, **syn. nov.** Type. Madagascar. Prov. Antsiranana: Analamazava, Binara Range, SW of Daraina (Vohemar), 13°15'S, 49°38'E, 26 Apr 1990, *D. Meyers 90* (holotype: K!; isotypes G00414721!, MO!, P [P00433104]!).

##### Type.

Madagascar. sin. loc., s.d., *R. Baron 5635* (lectotype, designated by Kainulainen et al. 2016, pg. 344: K [K001040371]!; isolectotypes: P [P00133213]!, P [P00133661]!).

##### Habit and distribution.

Shrubs; montane forests of Madagascar (Antananarivo, Antsiranana, Fianarantsoa, Toamasina, Toliara).

##### Notes.

Leandri (1939) considered *Croton hypochalibaeus* to be a synonym of *C. noronhae*, whereas Radcliffe-Smith (2016) considered it to be a synonym of *C. jennyanus*. *Croton hypochalibaeus* was accepted by Kainulainen et al. (2016), based on a number of distinguishing morphological and ecological criteria. It is one of the most wide-ranging *Croton* species in Madagascar.

#### 71. Croton ihosianus

Taxon classificationPlantaeMalpighialesEuphorbiaceae

Leandri, Adansonia, sér. 2, 9: 508. 1970, as ‘ihosiana’

Croton barorum var. mangokyensis Leandri, Ann. Mus. Colon. Marseille, sér. 5, 7(1): 68. 1939, **syn. nov.** Type. Madagascar. Prov. Fianarantsoa: Vallée d’Ihosy, bassin du Mangoky, 800-1000 m, 29-30 Oct 1924, *H. Humbert 2992* (lectotype, designated here: P [P00301485]!; isolectotypes: P [P00404482]!, P [P00127484]!).
Croton bathianus var. ihosianus Radcl.-Sm., Gen. Croton Madag. Comoro 114. 2016, **syn. nov.** Type. Madagascar. Prov. Fianarantsoa: environs d’Ihosy, Mar 1934, *H. Humbert 14445* (holotype: P [P00127476]!).

##### Type.

Madagascar. Prov. Fianarantsoa: environs d’Ihosy, Dec 1963, *J. Bosser 18722* (lectotype, designated here: P [P00328080]!; isolectotypes: P [P00133216]!, TAN!).

##### Habit and distribution.

Shrubs; south-central Madagascar (Fianarantsoa).

#### 72. Croton incisus

Taxon classificationPlantaeMalpighialesEuphorbiaceae

Baill., Adansonia 1: 159. 1861, as ‘incisum’

Oxydectes incisa (Baill.) Kuntze, Revis. Gen. Pl. 2: 612. 1891. Type. Based on Croton incisus Baill.
Croton incisus var. minor Leandri, Notul. Syst. (Paris) 13: 183. 1948, **syn. nov.** Type. Madagascar. Prov. Toamasina: Reserve Naturelle de Betampona, près de Tamatave; 450 m, 19 Feb 1938, *H.J. Lam & B. Meeuse 6014* (holotype: P [P00133221]!; isotypes: G [G00446382]!, K!, L, S!).

##### Type.

Madagascar. sin. loc., s.d., *L.M.A. Du Petit-Thouars s.n.* (holotype: P [P00389622]!).

##### Habit and distribution.

Shrubs; eastern lowland Madagascar (Toamasina).

##### Notes.

Some indices, such as Govaerts et al. (2000), have listed Baillon (1891b) as the publication in which *C. incisus* was described; however, the correct citation is Baillon (1861).

#### 73. Croton indrisilvae

Taxon classificationPlantaeMalpighialesEuphorbiaceae

Kainul., B.W.van Ee & P.E.Berry, Candollea 71: 338. 2016 [23 Nov 2016]

Croton commiphoroides Radcl.-Sm., Gen. Croton Madag. Comoro 21. 2016. [23 Dec 2016], **syn. nov.** Type. Madagascar. Prov. Toamasina: Périnet-Analamazaotra, 8-9 Aug 1961, *Service Forestier 20317* (holotype: P [P00133230]!).

##### Type.

Madagascar. Prov. Toamasina: Alaotra-Mangoro Region, Moramanga district, Analamazaotra National Park, on trail E of visitor center, 18°56'45"S, 48°25'33"E, 975 m, 11 Aug 2015, *B. van Ee, P.E. Berry & H. Razafindraibe 2175* (holotype: MICH [MICH1513201]!; isotypes: G, MO, TAN).

##### Habit and distribution.

Small shrubs; eastern montane Madagascar (Toamasina - Andasibe area).

#### 74. Croton inops

Taxon classificationPlantaeMalpighialesEuphorbiaceae

Baill., Bull. Mens. Soc. Linn. Paris 2: 864. 1890

##### Type.

Madagascar. Prov. Toliara: pays arides des Antandroi, Fort Dauphin, Jun-Jul, received Sep 1890, *G.F. Scott-Elliot 2986* (lectotype, designated here: P [P00133237]!; isolectotype: K [K000422592]!).

##### Habit and distribution.

Small shrubs; southern Madagascar (Toliara).

#### 75. Croton isalensis

Taxon classificationPlantaeMalpighialesEuphorbiaceae

(Leandri) Leandri, Adansonia, sér. 2, 12: 71. 1972

Croton brevispicatus var. isalensis Leandri, Ann. Mus. Colon. Marseille, sér. 5, 7(1): 28. 1939. Type. Madagascar. Prov. Fianarantsoa: Isalo, 1910, *H. Perrier de la Bâthie 9788* (lectotype, designated by Leandri 1972a, pg. 71: P [P00389628]!). Madagascar. Prov. Fianarantsoa: Isalo, 900 m, Oct 1924, *H. Perrier de la Bâthie 16607* (syntypes: K [K001040354]!, P [P00133240]!). Madagascar. Prov. Toliara: plateaux et vallées de l’Isalo, gorges de la Sakamarekely et de la Sambalinieto, 500-1000 m, 19-25 Oct 1924, *H. Humbert 2849* (syntypes: G [G00446377]!, G [G00446378]!, G [G00446379]!, K!, P [P00389626]!, P [P00389627]!, K [K001040353]! TAN [TAN000526]!).

##### Type.

Based on Croton brevispicatus var. isalensis Leandri

##### Habit and distribution.

Shrubs; southern Madagascar (Fianarantsoa, Toliara).

##### Notes.

In his publication elevating Croton brevispicatus var. isalensis to *C. isalensis*, Leandri (1972a) called the *Perrier de la Bâthie 9788* specimen at P the holotype. We interpret this as a lectotypification of the taxon, and given that there appears to only be a single duplicate of *Perrier de la Bâthie 9788* at P there is no need for a second-step lectotypification.

#### 76. Croton isomonensis

Taxon classificationPlantaeMalpighialesEuphorbiaceae

Leandri, Ann. Mus. Colon. Marseille, sér. 5, 7(1): 48. 1939

##### Type.

Madagascar. Prov. Toliara: vallée de la Manambolo, rive droite (basin du Mandrare) aux environs d’Isomono (confluent de la Sakamalio), Mont Morahariva (Mahamena), 1000–1400 m, Dec 1933, *H. Humbert 13247* (lectotype, designated here: P [P00133243]!; isolectotypes: G [G00018158]!, K [K001040341]!, P [P00133244]!, TAN [TAN000534]!). Madagascar. Prov. Toliara: vallée de la Sakamalio, affluent de la Manambolo (basin du Mandrare), 900-1100 m, Dec 1933, *H. Humbert 13367* (syntypes: P [P00133245]!, P [P00133246]!). Madagascar. Prov. Toliara: bassin de reception de la Mananara, affluent du Mandrare, pentes occidentales des montagnes entre l’Andohahela et l’Elakelaka au Vatazo (S d’Imonty), 900-950 m, Feb 1934, *H. Humbert 14076* (syntypes: P [P00133249]!, P [P00133250]!). Madagascar. Prov. Toliara: bassin de reception de la Mananara, affluent du Mandrare, pentes occidentales des montagnes entre l’Andohahela et l’Elakelaka entre Ampahiso et Mahamavo, 800 m, Jan-Feb.1934, *H. Humbert 13768* (syntype: G [G00018159]!).

##### Habit and distribution.

Large shrubs to small trees; southeastern Madagascar (Toliara).

##### Notes.

Leandri (1939) cited four Humbert collections as syntypes of *Croton isomonensis*: *13247*, *13367*, *14070*, and *13768*. We have not located a *Humbert 14070* specimen, but the *Humbert 14076* collection has the verbatim collection locality as given in Leandri (1939) for *Humbert 14070*. We surmise that the ‘*14070*’ in Leandri (1939) is a typographic error for ‘*14076*.’

#### 77. Croton jennyanus

Taxon classificationPlantaeMalpighialesEuphorbiaceae

Gris ex Baill., Adansonia 1: 160. 1861, as ‘jennyanum’

Croton squamiger Baill., Adansonia 1: 168. 1861, as ‘*squamigerum*’. Type. Madagascar. Prov. Antsiranana: sin. loc., s.d., *J.M.C. Richard 576* (lectotype, designated here: P [P00133578]!; isolectotype: P [P00133579]!). Madagascar. Prov. Antsiranana: Diégo-Suarès et baie de Rigny, 1837, *J.M.C. Richard 176* (syntype: P [P00133577]!). Madagascar. Prov. Antsiranana: Cap d’Ambre, 1847-1852, *L.H. Boivin 2658* (syntype: P [P00133273]!).
Croton squamiger var. obtusifolius Müll.Arg. in A.P.de Candolle, Prodr. 15(2): 523. 1866, nom. inval. Type. Based on Croton squamiger Baill.
Oxydectes jennyana (Gris ex Baill.) Kuntze, Revis. Gen. Pl. 2: 612. 1891. Type. Based on Croton jennyanus Gris ex Baill.
Oxydectes squamigera (Baill.) Kuntze, Revis. Gen. Pl. 2: 613. 1891. Type. Based on Croton squamiger Baill.

##### Type.

Madagascar. Prov. Antsiranana [Diana Region]: vers les bas-fonds humides, lorsqu’on s’élève par les torrents au dessus des montagnes qui se trouvent au nord du port Lugwata [Diego Suarez], 1833, *J.P. Goudot s.n.* (holotype: G [G00434419]!; isotypes: P [P00133278]!, P [P00154519]!).

##### Habit and distribution.

Shrubs; northern and western Madagascar (Antsiranana, Mahajanga).

##### Notes.

We follow here the precedent of Leandri (1939) and Radcliffe-Smith (2016) in treating *Croton squamiger* as a synonym of *C. jennyanus*, but we differ in treating *C. hypochalibaeus* as a distinct, more highland species rather than as another synonym of *C. jennyanus* (see Kainulainen et al., 2016). According to our interpretation, *C. jennyanus* is restricted to lower elevations in northern Madagascar (Montagne des Français, Sahafary, Daraina, Ankarana), as well as in far midwestern Madagascar on or near tsingy formations (Bemaraha).

Concerning the type of *Croton squamiger*, three other sheets of *Boivin 2658* correspond to *C. brevispicatus*, so this is clearly a mixed collection. In the description of *C. squamiger*, Baillon (1861) divided the species into two infraspecific taxa, a and b. These were subsequently named by Müller (1866) as C. squamigerus var. obtusifolius (the typical variety) and C. squamigerus var. acutifolius, respectively. The latter has been recognized here to be synonymous with *C. hypochalibaeus*.

We have corrected the termination of the specific epithet to “*squamiger*” [masc. gender, Adj. group A nom., see Stearn (1992: 91) and Art. 23.5 and Art. 60.9 Ex. 24 (ICN 2012; *Croton ciliato-glanduliferum* Ortega corrected to *C. ciliatoglandulifer*).

Radcliffe-Smith (2016) listed *Croton jennyanus* as being present in the Comoros Archipelago. The three collections he cited [*Pascal 928* (K, P) from Mayotte, and *Floret 1241* (P) and *1249* (P) from Mohéli] all correspond to *C. humblotii*.

#### 78. Croton kimosorum

Taxon classificationPlantaeMalpighialesEuphorbiaceae

Leandri, Ann. Mus. Colon. Marseille, sér. 5, 7(1): 29. 1939

Croton kimosorum var. pubescens Radcl.-Sm., Gen. Croton Madag. Comoro 73. 2016, **syn. nov.** Type. Madagascar. Prov. Toliara: NW of Tôlañaro, Andohahela Réserve Intégrale, 24°57'S, 46°39'E, 23 Dec 1993, *S. Malcomber 2642* (holotype: K!; isotype: MO!).

##### Type.

Madagascar. Prov. Toliara: vallé de la Sakamalio, affluent de la Manambolo (basin du Mandrare), 500-800 m, Dec 1933, *H. Humbert 13320* (holotype: P [P00133294]!; isotypes: G [G00018156]!, K [K001040384]!, TAN [TAN000536]!).

##### Habit and distribution.

Shrubs; southern Madagascar (Toliara).

#### 79. Croton lasiopyrus

Taxon classificationPlantaeMalpighialesEuphorbiaceae

Baill., Bull. Mens. Soc. Linn. Paris 2: 926. 1891

##### Type.

Madagascar: “Central Madagascar”, Oct 1882, *R. Baron 1951* (lectotype, designated by Kainulainen et al. 2016, pg. 350: P [P00133406]!, isolectotypes: K [K001040378]!, P [P00133407]!). Madagascar: “Central Madagascar”, Oct 1882, *R. Baron 2114* (syntypes: K [K001040377]!, P [P00133408]!); “Central Madagascar”, s.d., *R. Baron 4078* (K [K001040376]!). Madagascar. Prov. Toliara: Fort-Dauphin, s.d., *G.F. Scott-Elliot 1557* (syntype: P [P00133052 packet in upper right]!).

##### Habit and distribution.

Shrubs; eastern montane forests (Antananarivo, Toamasina).

##### Notes.

See the note above under *Croton cassinioides* and its synonym *C. delphinianus* regarding the *Scott-Elliot 1557* specimen (P00133052, upper right), which was also cited by Baillon (1891a) as a syntype of *C. lasiopyrus*. *Croton cassinioides* and *C. lasiopyrus* are sufficiently different that they are not easily confused; the former has smaller (1.5–6 ×0.7–3 cm) elliptic leaves with dentate to subentire margins and grows in littoral zones near sea level, while the latter has larger (4–15.5 × 2.5–7 cm) obovate leaves with entire margins and grows in moist montane forests.

#### 80. Croton lichenisilvae

Taxon classificationPlantaeMalpighialesEuphorbiaceae

Leandri, Ann. Mus. Colon. Marseille, sér. 5, 7(1): 62. 1939

Croton lichenisilvae var. oligostemon Radcl.-Sm., Gen. Croton Madag. Comoro 198. 2016, **syn. nov.** Type. Madagascar. Prov. Toamasina: Anony, Sihanaka, 3 Sep 1937, *Herb. Jard. Bot. Tan. 2953* (holotype: P [P00131512]!).

##### Type.

Madagascar. Prov. Toamasina: Alaotra-Mangoro Region, environs d’Analamazaotra, s.d., 1000 m, *H. Perrier de la Bâthie 9637* (lectotype, designated here: P [P00389499]!; isolectotype: P [P00133451]!).

##### Habit and distribution.

Shrubs; eastern montane Madagascar (Toamasina).

#### 81. Croton loucoubensis

Taxon classificationPlantaeMalpighialesEuphorbiaceae

Baill., Adansonia 1: 155. 1861, as ‘loucoubense’

Croton adenophorus var. loucoubensis (Baill.) Müll.Arg. in A.P.de Candolle, Prodr. 15(2): 589. 1866. Type. Based on Croton loucoubensis Baill.
Croton adenophoroides Radcl.-Sm., Gen. Croton Madag. Comoro 117. 2016. Type. Madagascar. Prov. Antsiranana: Besinkara, Ambalafary, Andvakena: premier cours d’eau sur le chemin de Bekolosy, 14°04'S, 48°17'E, 500 m, 12 Nov 1994, *L. Gautier & P. Derleth 2529* (holotype: K!; isotypes: G!, MO!, P [P00433174]!).

##### Type.

Madagascar. Prov. Antsiranana: Diana Region, Nossibé, forêt de Loucoubé, Mar 1851, *L.H. Boivin s.n.* (lectotype, designated by Kainulainen et al. 2017b, pg. 390: P [P00133453]!). Mayotte [French Overseas Department]: Jun 1848, *L.H. Boivin 3382* (syntype: P [P00133452]!).

##### Habit and distribution.

Large shrubs or small trees; northern Madagascar (Antsiranana).

##### Notes.

Leandri (1939) treated *Croton loucoubensis* as a synonym of *C. adenophorus*, but his concept of *C. adenophorus*, as shown in his key and description, conforms to the type of *C. loucoubensis* as treated here. The syntype of *C. loucoubensis* from Mayotte is sterile and cannot definitively be placed in this species; it could also potentially belong to *C. mayottae*.

#### 82. Croton macrobuxus

Taxon classificationPlantaeMalpighialesEuphorbiaceae

Baill., Bull. Mens. Soc. Linn. Paris 2: 863. 1890

Croton sambiranensis Leandri, Ann. Mus. Colon. Marseille, sér. 5, 7(1): 41. 1939. Type. Madagascar. Prov. Antsiranana: Haut Sambirano, 500 m, Dec 1912, *H. Perrier de la Bâthie 9699* (lectotype, designated here: P [P00133571]!; isolectotypes: P [P00133572]!, P [P00404491]!).
Croton macrobuxus var. dolichobotrys Radcl.-Sm., Gen. Croton Madag. Comoro 176. 2016, **syn. nov.** Type. Madagascar. Prov. Toamasina: Ambatovy, NE of Moramanga, 18°51'07"S, 48°18'26"E, 28 Feb 1998, *G. McPherson 17500* (holotype: K; isotype: MO!).
Croton macrobuxus var. glandulifer Radcl.-Sm., Gen. Croton Madag. Comoro 176. 2016, **syn. nov.** Type. Madagascar. Prov. Toamasina: Ambatovy, 18°51'25"S, 48°17'50"E, 28 Feb 1997, *P. Rakotomalaza et al. 1194* (holotype: K; isotype: MO, P [P00433501]!).
Croton macrobuxus var. subfoliaceus Radcl.-Sm., Gen. Croton Madag. Comoro 177. 2016, **syn. nov.** Type. Madagascar. Prov. Toamasina: Torotorofotsy R., Berano, 15 air-km NE of Moramanga, 11 km E of Antanambao, between Ambatovy-South & Analamay-East, 18°50'32"S, 48°19'55"E, 20 Feb 1997, *P. Rakotomalaza*, *G. Razafimanantsoa* & *F. Andriatsiferana 1149* (holotype: MO!).
Croton macrobuxus var. substrigosus Radcl.-Sm., Gen. Croton Madag. Comoro 177. 2016, **syn. nov.** Type. Madagascar. Prov. Antananarivo: -Manjakandriana, Ankatsohazo, 29 Oct 1968, *R. Razafindrambao H534R* (holotype: P [P00154348]!).
Croton nitidulus var. microphyllus Radcl.-Sm., Gen. Croton Madag. Comoro 173. 2016, **syn. nov.** Type. Madagascar. Prov. Antsiranana: Reserve Naturelle Marojejy, along the trail to the summit of Marojejy Est, 14°26'S, 49°15'E, 10 Oct 1988, *J. Miller et al. 3524* (holotype: K!; isotype: MO!).
Croton nitidulus var. pubescens Radcl.-Sm., Gen. Croton Madag. Comoro 173. 2016, **syn. nov.** Type. Madagascar. Prov. Antsiranana: Marojejy, N d’Andapa, 14°29'S, 49°38'E, coll. 21-22 Jan 1994, *F. Rasoavimbahoaka et al. 30* (holotype: K!; isotype: MO!).

##### Type.

Madagascar. Madag. Centr., s.d., *R. Baron 3063* (lectotype, designated here: K [K001040373]!; isolectotype: P [P00133472]!).

##### Habit and distribution.

Shrubs; upland forests of Madagascar (Antananarivo, Antsiranana, Fianarantsoa, Mahajanga, Toamasina, Toliara).

##### Notes.

Both Croton nitidulus var. microphyllus and C. nitidulus var. pubescens from high elevations in the Marojejy massif are smaller-leaved plants than most other specimens of *C. macrobuxus*. They should be studied in further detail to determine if they should be recognized as a distinct, related species.

#### 83. Croton maevaranensis

Taxon classificationPlantaeMalpighialesEuphorbiaceae

Leandri, Adansonia, sér. 2, 9: 501. 1970

##### Type.

Madagascar. Prov. Antsiranana: Massif de l’Ambohimirahavavy, rebord Sud du plateau de Marofamano, 6 Feb 1951, *Service des Eaux et Forêts de Madagascar 984-SF* (lectotype, designated here: P [P00389625]!; isolectotypes: K [K000422594]!, MO [sheet #04861162], P [P00389623]!, P [P00389624]!, TAN!, TEF [TEF000186]!).

##### Habit and distribution.

Trees; northern and northwestern Madagascar (Antsiranana, Mahajanga).

#### 84. Croton manampetsae

Taxon classificationPlantaeMalpighialesEuphorbiaceae

Leandri, Ann. Mus. Colon. Marseille, sér. 5, 7(1): 25. 1939

Croton manampetsae var. angustifolius Radcl.-Sm., Gen. Croton Madag. Comoro 27. 2016, **syn. nov.** Type. Madagascar. Prov. Toliara: Beza Mahafaly Reserve, near Betioky, 23°41'S, 44°35'E, 28 Oct 1987, *P.B. Phillipson 2473* (holotype: K; isotypes: MO, P [P00133494]!).
Croton manampetsae var. chaetogyne Radcl.-Sm., Gen. Croton Madag. Comoro 27. 2016, **syn. nov.** Type. Madagascar. Prov. Toliara: Fort Dauphin, Ranopiso, Manatalinjo, Réserve Naturelle Intégrale d’Andohahela, 24°49'S, 46°37'E, 26-30 Oct 1994, *S. Eboroke 875* (holotype: K; isotypes: MO, P [P00433495]!).

##### Type.

Madagascar. Prov. Toliara: Lac Manampetsa, Apr 1933, *H. Perrier de la Bâthie 19087* (lectotype, designated here: P [P00133491!); isolectotypes: K [K001040386]!, P [P00133492]!).

##### Habit and distribution.

Shrubs; southern Madagascar (Toliara).

##### Notes.

Except for Croton manampetsae var. lepidotus (see Incertae Sedis section), the varieties recognized by Radcliffe-Smith (2016) appear to be inconsequential forms of the same species, with minor variations in pubescence and leaf shape.

#### 85. Croton mauritianus

Taxon classificationPlantaeMalpighialesEuphorbiaceae

Lam., Encycl. 2: 206. 1786

Halecus mauritianus (Lam.) Raf., Sylva Tellur.: 62. 1838. Type: Based on Croton mauritianus Lam.
Klotzschiphytum mauritianum (Lam.) Baill., Étude Euphorb.: 383. 1858. Type: Based on Croton mauritianus Lam.
Oxydectes mauritiana (Lam.) Kuntze, Revis. Gen. Pl. 2: 612. 1891. Type: Based on Croton mauritianus Lam.

##### Type.

Réunion [French Overseas Department]: Île de Bourbon, s.d., *P. Commerson s.n.* (holotype: P-LA [P00382069]!; isotypes: G-DC [G00311200]!, G [G00446393]!, G [G00446394]!, MPU [MPU014846]!, MPU [MPU014847]!, MPU [MPU014848]!, P [P00121732]!, P [P00404317]!, P-JU Catal. 16377!, P [P00404321]!, P [P00404316]!, P [P00404318]!; possible isotypes, K [K001040358]!, P [P00404319]!, P [P00404320]!).

##### Habit and distribution.

Large shrubs or small trees; restricted to the island of Réunion.

##### Notes.

Lamarck clearly attributed this species to the Île de Bourbon, the former name of Réunion, so it is unclear why he named it “mauritianus.” At the time of Lamarck's publication, both Réunion (Île de Bourbon) and Mauritius (Île de France) were occupied by the French, and Réunion was administered out of Port Louis, Mauritius. So perhaps Lamarck used the name in a general sense for the islands administered out of Mauritius.

*Croton mauritianus* Lam. is the type of Croton sect. Klotzschiphytum Baill. (Baillon, 1861), which is one of the few described sections of *Croton* with an Old World species as its type.

#### 86. Croton mavoravina

Taxon classificationPlantaeMalpighialesEuphorbiaceae

Leandri, Ann. Mus. Colon. Marseille, sér. 5, 7(1): 25. 1939

Croton capuronii Leandri, Bull. Soc. Bot. France 103: 604. 1957 [‘1956’]. Type. Madagascar. Prov. Toliara: Anosy Region: environs de Bevilany, route Ambovombe-Fort Dauphin, 23 Sep 1953, *R. Capuron 8493bis* (lectotype, designated here: P [P00312141]!; isolectotype: P [P00347487]!). Madagascar. Prov. Toliara: environs de Fort Dauphin, près de Bevilany, 14 Sep 1928, *H. Humbert & C.F. Swingle 5673* (syntypes: G, K, P [P00133508]!).
Croton mavoravina var. gracilis Radcl.-Sm., Gen. Croton Madag. Comoro 31. 2016, **syn. nov.** Type. Madagascar. Prov. Toliara: Beza Mahafaly Reserve near Betioky, 23°40'S, 44°35'E, 5 Jan 1988, *P.B. Phillipson 2785* (holotype: K; isotypes: MO, P [P00133515]!).
Croton mavoravina var. gymnolepis Radcl.-Sm., Gen. Croton Madag. Comoro 31. 2016, **syn. nov.** Type. Madagascar. Prov. Toliara: District d’Amboasary, Canton de Tranomaro, Ambatomika, 25 May 1957, *G. Cours 5221* (holotype: P [P00133507]!).
Croton mavoravina var. imanombensis Radcl.-Sm., Gen. Croton Madag. Comoro 32. 2016, **syn. nov.** Type. Madagascar. Prov. Toliara: Imanombo, Nov 1952, *J.M. Bosser 3837* (holotype: P [P00133495]!).
Croton mavoravina var. rotundifolius Radcl.-Sm., Gen. Croton Madag. Comoro 32. 2016, **syn. nov.** Type. Madagascar. Prov. Toliara: Beza Mahafaly Reserve near Betioky, hills E of the Sakamena River, valley of the Ambinda stream, 23°40'S, 44°39'E, 26 Oct 1987, *P.B. Phillipson 2450* (holotype: K; isotypes: MO, P [P00133513]!).

##### Type.

Madagascar. Prov. Toliara: de Tsivory à Anadabolava, Mandrare moyen, 300-400 m, Dec 1933, *H. Humbert 12319* (lectotype, designated here: P [P00312342]!; isolectotype: P [P00133510]!).

##### Habit and distribution.

Shrubs; southern Madagascar (Toliara).

##### Notes.

A fourth variety of *Croton mavoravina* that was described by Radcliffe-Smith (2016) as C. mavoravina var. thysanolepis is not part of this species at all, but rather is a synonym of *C. crossolepis*.

#### 87. Croton mayottae

Taxon classificationPlantaeMalpighialesEuphorbiaceae

P.E.Berry & Kainul., Candollea 72: 392. 2017

Croton regeneratrix var. mayottensis Radcl.-Sm., Gen. Croton Madag. Comoro 202. 2016. Type. Mayotte [French Overseas Department]: Rassi Maoussi, 30 m, 24 Apr 997, *O. Pascal 915* (holotype: K!; isotypes: BR, G!, K, MAO!, MO!, P [P00144592]!, WAG).

##### Type.

Mayotte [French Overseas Department]: Grande-Terre, Chiconi, village, 16 Jan 2001, *F. Barthelat, M’Changama & A.B. Sifary 225* (holotype: P [P00229211]!; isotypes: G!, K!, MAO!, MO!).

##### Habit and distribution.

Shrubs; endemic to the island of Mayotte in the Comoro Islands.

#### 88. Croton menabeensis

Taxon classificationPlantaeMalpighialesEuphorbiaceae

Leandri, Adansonia, sér. 2, 12: 68. 1972

Croton subaemulans var. minor Leandri, Ann. Mus. Colon. Marseille, sér. 5, 7(1): 82. 1939, **syn. nov.** Type. Madagascar. Prov. Mahajanga: causse d’Ankara, Kamakama, Dec 1900, *H. Perrier de la Bâthie 9814* (lectotype, designated here: P [P00133377]!; isolectotype: P [P00133376]!). Madagascar. Prov. Mahajanga: embouchure de la , Tsiampihy, 15 Oct 1932, *J. Leandri 298* (syntypes: K [K001040361]!, P [P00133370]!).
Croton delicatulus Radcl.-Sm., Gen. Croton Madag. Comoro 20. 2016, **syn. nov.** Type. Madagascar. Prov. Mahajanga: station forestière d’Ampijoroa, ca. 3 km N d’Andranofasika, 16°20'S, 46°51'E, 8 Apr 1984, *L. Dorr* & *L. Koenders 2962* (holotype: P [P00131505]!; isotypes: K!, MO!).
Croton neoholstiifolius Radcl.-Sm., Gen. Croton Madag. Comoro 120. 2016, **syn. nov.** Type. Madagascar. Prov. Mahajanga: Tsingy de Bemaraha, Antsalova-Tsiandro Berano, 18°39'S, 44°44'E, 24 Nov 1992, *J. Labat, T. Deroin, R. Edmond, H. Rabarion & O. Laivao 2153* (holotype: K!; isotype: P [P05606030]!).

##### Type.

Madagascar. Prov. Mahajanga: Antsingy, vers Andobo (E d’Antsalova), vers Tsiandro, 300 m, 5-8 Feb 1960, *J. Leandri & P. Saboureau 3021bis* (lectotype, designated here: P [P00389507]!; isolectotype: P [P00132918]!).

##### Habit and distribution.

Shrubs; western Madagascar (Mahajanga, Toliara).

#### 89. Croton menarandrae

Taxon classificationPlantaeMalpighialesEuphorbiaceae

Leandri, Adansonia, sér. 2, 10: 189. 1970

Croton menarandrae var. pubescens Radcl.-Sm., Gen. Croton Madag. Comoro 87. 2016, **syn. nov.** Type. Madagascar. Prov. Toliara: Environs d’Ampandrandava, entre Bekily et Tsivory, Oct 1942, *A. Seyrig 139bis* (holotype: P [P00154452]!).

##### Type.

Madagascar. Prov. Toliara: Berge Menarandra, Ampanihy, 25 Sep 1953, *Service Forestier 8273* (lectotype, designated here: P [P00347525]!; isolectotypes: P [P00132921]!, TEF [TEF000187]!).

##### Habit and distribution.

Shrubs; southern Madagascar (Toliara).

#### 90. Croton meridionalis

Taxon classificationPlantaeMalpighialesEuphorbiaceae

Leandri, Ann. Mus. Colon. Marseille, sér. 5, 7(1): 59. 1939

Croton sublinearis Leandri, Ann. Mus. Colon. Marseille, sér. 5, 7(1): 60. 1939. Type. Madagascar. Prov. Toliara: Lahimanara, env. d’Ambovombe, 8 Jun 1931, *R. Decary 8991* (holotype: P [P00133597]!; isotypes: G [G00018136]!, K [K001040363]!, S [S07-16870], TAN).
Croton tranomarensis Leandri, Ann. Mus. Colon. Marseille, sér. 5, 7(1): 60. 1939, **syn. nov.** Type. Madagascar. Prov. Toliara: Anosy Region, Tranomaro, au NE d’Ambovombe, 19 Jun 1931, *R. Decary 9022* (lectotype, designated here: P [P00133389]!; isolectotypes: G [G00018135]!, K [K001040392]!, S [S07-16871], TAN [TAN000544]).
Croton meridionalis var. pseudolepidotus Radcl.-Sm., Gen. Croton Madag. Comoro 194. 2016, **syn. nov.** Type. Madagascar. Prov. Fianarantsoa: 47-49 km SE of Ihosy on the road to Ivohibe, 5 Nov 1967, *L. Bernardi 11179* (holotype: K!; isotypes: G!, P [P00154376]!).

##### Type.

Madagascar. Prov. Toliara: bassin supérieur du Mandrare (Sud-Est), vallée de la Manombolo, 300-400 m, 23-24 Nov 1928, *H. Humbert 6798* (lectotype, designated here: P [P00132930]!; isolectotypes: G [G00018153]!), P [P00132929]!). Madagascar. Prov. Toliara: plateaux et vallées de l’Isalo, environs de Fanjahira 300-600 m, 9-12 Oct 1924, *H. Humbert 2737* (syntype: P [P00132928]!). Madagascar. Prov. Toliara: Vallée de l’Onilahy, aux environs de Tongobory, 100-200 m, 1-8 Oct 1924, *H. Humbert 2737* (syntypes: G [G00018154]!, P [P00132927]!).

##### Habit and distribution.

Shrubs; southern Madagascar (Fianarantsoa, Toliara).

##### Notes.

Most individuals of this species have pairs of falcate, foliaceous, clasping stipules and stellate pubescence. Plants with similar stipules, but usually broader and more silvery-lepidote lower leaf surfaces were described under Croton meridionalis var. latifolius and C. meridionalis var. stipularis, which may represent a different species (see Incertae Sedis).

#### 91. Croton miarensis

Taxon classificationPlantaeMalpighialesEuphorbiaceae

Leandri, Adansonia, sér. 2, 9: 504. 1970

Croton peltieri Leandri, Adansonia, sér. 2, 10: 184. 1970, **syn. nov.** Type. Madagascar. Prov. Toliara: Lac Manampetsa (“Tsimanampetsotsa”), 23 Oct 1940, *R. Decary 16060* (lectotype, designated here: P [P00338571]!; isolectotypes: P [P00154280]!, P [P00154281]!).
Croton peltieri var. hazofotsiensis Radcl.-Sm., Gen. Croton Madag. Comoro 74. 2016, **syn. nov.** Type. Madagascar. Prov. Toliara: Hazofotsy Reserve 11, 28 Apr 1971, *A. Richard 123* (holotype: K!).
Croton miarensis var. monadenius Radcl.-Sm., Gen. Croton Madag. Comoro 76. 2016, **syn. nov.** Type. Madagascar. Prov. Toliara: Cap Sainte-Marie et environs Nord du Cap, 17 Dec 1968, *Service Forestier 28552*-*SF* (lectotype, designated here: P [P00154277]!; isolectotype: P [P00154276]!).

##### Type.

Madagascar. Prov. Toliara: Miari, Nov 1956, *J. Bosser 10492* (holotype: P [P00312379]!, isotype TAN!).

##### Habit and distribution.

Shrubs; southern Madagascar (Toliara).

##### Notes.

This is a very distinctive species from southern Madagascar with silvery, ovate, long-petiolate leaves that usually have a single acropetiolar gland. It is also very unusual in having 1- or 2-locular capsules, and the branching is strongly dichotomous. All of the taxa placed here in synonymy share these features.

#### 92. Croton milanjensis

Taxon classificationPlantaeMalpighialesEuphorbiaceae

Leandri, Adansonia, sér. 2, 12: 66. 1972

##### Type.

Madagascar. Prov. Mahajanga: Milanja, près de Soalala, 18 Nov 1954, *Conservation de Réserves Naturelles 6863RN* (holotype: P [P00389506]!).

##### Habit and distribution.

Shrubs; western Madagascar (Mahajanga).

#### 93. Croton minimimarginiglandulosus

Taxon classificationPlantaeMalpighialesEuphorbiaceae

Radcl.-Sm., Gen. Croton Madag. Comoro 18. 2016.

Croton fianarantsoae var. ambremontanus Radcl.-Sm., Gen. Croton Madag. Comoro 153. 2016, **syn. nov.** Type. Madagascar. Prov. Antsiranana: National Park of Montagne d’Ambre, path from station to Bianamalao, 12°32'S, 49°10'E, 1120 m, 4 Jun 1989, *B. Du Puy, D. Du Puy, & G. Guy 218* (holotype: K!; isotype: P [P00060699]!).
Croton ivohibensis var. aesculops Radcl.-Sm., Gen. Croton Madag. Comoro 148. 2016, **syn. nov.** Type. Madagascar. Prov. Antsiranana: Reserve Spéciale d’Ankarana, 12°54'43"S, 49°06'39"E, 19 Feb 1994, *M. Andrianarisata et al. 43* (holotype: K!; isotypes: MO!, P [P00433266]!).
Croton ivohibensis var. ankaranaensis Radcl.-Sm., Gen. Croton Madag. Comoro 148. 2016, **syn. nov.** Type. Madagascar. Prov. Antsiranana: Reserve Naturelle Intégrale d’Ankarana, 12°51'S, 49°05'E, 18 May 1987, *M. Nicoll* & *J.P. Abraham 684* (holotype: K!; isotypes: MICH [MICH1514788]!, MO!, P [P00133271]!).
Croton thouarsianus var. macrocalyx Radcl.-Sm., Gen. Croton Madag. Comoro 183. 2016, **syn. nov.** Type. Madagascar. Antsiranana: Ankarana Reserve, Tsingy area, 12°54'42"S, 49°06'42"E, 22 May 1993, *C.C.H. Jongkind* & *S. Rapanarivo 960* (holotype: K!; isotypes: MICH [MICH1514787]!, MO!, P [P00433151]!).

##### Type.

Madagascar. Prov. Antsiranana: au sud d’Antsiranana, près de Joffreville dans le Parc National de Montagne d’Ambre, 12°27'S, 49°13'E, 3-10 Aug 1993, *O. Andrianantoanina* & *Rocsceohclher 283* (holotype: K!; isotype: MO!).

##### Habit and distribution.

Shrubs; northern Madagascar (Antsiranana).

##### Notes.

This species is restricted to Montagne d’Ambre and tsingy habitats in the Ankarana Special Reserve. It has somewhat anisophyllous terminal leaves like *C. thouarsianus*, but the calyx of the pistillate flowers is much larger and more accrescent in fruit, with a long pedicel. Plants vary considerably in pubescence, with some hirsute stems in the type of *C. thouarsianus* var. macrocalyx. Quoting from Radcliffe-Smith (2016), “The admittedly rather cumbersome name, of 10 syllables & 24 letters, can be justified on the grounds of the distinctive minute ogivaliform marginal glands to be found on the otherwise entire leaf-margins of this species. The name is not occupied in the genus.” No surprise there!

#### 94. Croton mocquerysii

Taxon classificationPlantaeMalpighialesEuphorbiaceae

Aug.DC., Bull. Herb. Boissier, sér. 2, 1: 565. 1901, as ‘moquerysi’

##### Type.

Madagascar. Prov. Toamasina: Maroa [Maroantsetra], forêts à l’intérieur de la baie d’Antongil, 1897, *A. Mocquerys 256* (holotype: G [G00018152]!; isotype: Z [Z-000015988]!).

##### Habit and distribution.

Shrubs or small trees; eastern lowland Madagascar (Antsiranana, Toamasina).

#### 95. Croton mongue

Taxon classificationPlantaeMalpighialesEuphorbiaceae

Baill., Adansonia 1: 158. 1861

Oxydectes mongue (Baill.) Kuntze, Revis. Gen. Pl. 2: 612. 1891. Type. Based on Croton mongue Baill.
Croton oreades Leandri, Ann. Mus. Colon. Marseille, sér. 5, 7(1): 72. 1939, **syn. nov.** Type. Madagascar. Prov. Toamasina: Analamazaotra, 800 m, Dec, *H. Perrier de la Bâthie 5287* (lectotype, designated here: P [P00389513]!; isolectotypes: P [P00389512]!, P [P00389514]!). Madagascar. Prov. Fianarantsoa: haute vallée de la Rienana, province de Farafangana, 3 Oct 1926, *R. Decary 5563* (syntypes: K [K001040348]!, P [P00133687]!). Madagascar. Prov. Fianarantsoa: Sud-Est de Fianarantsoa, 1000-1200 m, 27 Oct 1926, *R. Decary 5842* (syntypes: P [P00133688]!, S [S07-17117]!).
Croton oreades var. occidentalis Leandri, Ann. Mus. Colon. Marseille, sér. 5, 7(1): 73. 1939, **syn. nov.** Type. Madagascar. Prov. Mahajanga: plateau de Miangaka [Marangaka, ], 1000 m, Dec 1922, *H. Perrier de la Bâthie 15130* (lectotype, designated here: P [P00133691]!; isolectotype: P [P00133690]!).
Croton mongue var. vatambensis Leandri, Ann. Mus. Colon. Marseille, sér. 5, 7(1): 72. 1939. Type. Madagascar. Prov. Toliara: Vatambe, Fort-Dauphin, 7 Sep 1932, *R. Decary 10601* (holotype: P [P00132950]!, isotypes: K [K001040389]!, TAN [TAN000537]!).
Croton mongue var. borealis Radcl.-Sm., Gen. Croton Madag. Comoro 131. 2016, **syn. nov.** Type. Madagascar. Prov. Antsiranana: Réserve Naturelle Intégrale no. 12 de Marojejy, le long d’un affluent de la rivière Manantenina, 11 km NW de village Manantenina, 14°26'S, 49°44'E, 1220 m, 27 Oct 1996, *P.J. Rakotomalaza, N. Messmer & D. Ravelonarivo 793* (holotype: K!; isotypes: MO!, P [P00433430]!).
Croton oreades var. borealis Radcl.-Sm., Gen. Croton Madag. Comoro 132. 2016, **syn. nov.** Type. Madagascar. Prov. Mahajanga: Befandriana-Nord, Matsoandakana, Réserve Spéciale Anjanaharibe-Sud, village d’Anjiamazava, suivant la route Nationale d’Andapa-Bealanana, piste vers le Nord approchant le sommet de Bevitsika, 14°42'S, 49°27'E, 1100 m, 14 Dec 1994, *D. Ravelonarivo & R. Rabesonina 551* (holotype: K!; isotype: MO!).
Croton oreades var. craspedadenius Radcl.-Sm., Gen. Croton Madag. Comoro 132. 2016, **syn. nov.** Type. Madagascar. Prov. Antsiranana: Andapa, Ambalamanasy II, Andasibe , suivant la piste entre Andasibe et Andranovola, dans la Réserve Naturelle Intégrale de Marojejy, 14°31'S, 49°38'E, 603 m, 1-3 Feb 1994, *F. Rasoavimbahoaka et al. 86* (holotype: K!; isotypes: MO!, P [P00433034]!).
Croton oreades var. periphoradenius Radcl.-Sm., Gen. Croton Madag. Comoro 133. 2016, **syn. nov.** Type. Madagascar. Prov. Fianarantsoa: Ampamaherana, 29 Sep 1949, *Service Forestier 2033-SF* (holotype: P [P00132967]!).

##### Type.

Madagascar. sin. loc., s.d., *L.A. Chapelier s.n.* (lectotype, designated here: P [P00132944]!; isolectotype: P [P00132945]!).

##### Habit and distribution.

Trees; montane forests of Madagascar (Antananarivo, Antsiranana, Fianarantsoa, Mahajanga, Toamasina, Toliara).

##### Notes.

It is surprising to us that neither Leandri (1939) nor Radcliffe-Smith (2016) realized that *Croton oreades* is not distinct from *C. mongue*. This is the largest tree species among Malagasy *Croton*, and it is widespread in moist, montane forests.

#### 96. Croton multicostatus

Taxon classificationPlantaeMalpighialesEuphorbiaceae

Müll.Arg., Linnaea 34: 79 (1865)

Oxydectes multicostata (Müll.Arg.) Kuntze, Revis. Gen. Pl. 2: 612. 1891. Type: Based on Croton multicostatus Müll.Arg.
Croton vernicosus Baker in J. Linn. Soc., Bot. 22: 519. 1887. Type. Madagascar. sin. loc., s.d., *R. Baron 4935* (lectotype, designated here: K [K000347500]!, isolectotype: K [K000347498]!).
Croton sclerodorus Baill., Bull. Mens. Soc. Linn. Paris 2: 968. 1891, as ‘*sclerodorum*’. Type. Madagascar. sin. loc., s.d., *R. Baron 4735* (holotype: P [P00133318]!).

##### Type.

Madagascar. Prov. Toliara: Fort Dauphin, no collector indicated, (holotype: P-JU [Catalogue # 16338]!; isotype: *Madagascar j. maut. No. 39*, P-LA [P00382066]!).

##### Habit and distribution.

Large shrubs or small trees; southeastern Madagascar (Toliara, possibly also in Fianaranstsoa).

##### Notes.

See Berry and Van Ee (2011) for a discussion of how Müller (1865) mistook the type locality of *Croton multicostatus* for the Caribbean (Hispaniola) rather than for Madagascar. In that paper, we attributed both the P-JU and P-LA sheets as being a Philibert Commerson collection, but more likely these were collected by Franz Wilhelm Sieber, who visited Madagascar between 1822 and 1825, and included them under his series “j. maut.”, as indicated on the P-LA sheet.

#### 97. Croton muricatus

Taxon classificationPlantaeMalpighialesEuphorbiaceae

Vahl in E.F.Geiseler, Croton. Monogr.: 47. 1807

Oxydectes muricata (Vahl) Kuntze, Revis. Gen. Pl. 2: 612. 1891. Type. Based on Croton muricatus Vahl in E.F.Geiseler
Croton denisii Leandri, Bull. Mus. Natl. Hist. Nat., sér. 2, 3: 367. 1931, as ‘*denisi*’, **syn. nov.** Type. Madagascar. Prov. Toliara: Behara, 9 Jul 1926, *R. Decary 4320* (holotype: P [P00389632]!).
Croton bastardii var. meridionalis Radcl.-Sm., Gen. Croton Madag. Comoro 204. 2016, **syn. nov.** Type. Madagascar. Prov. Toliara: Antanimora, Dec 1959, *J. Bosser 13913* (holotype: P [P00133042)!; isotype: [P00133041]!).
Croton decaryi var. subglaber Radcl.-Sm., Gen. Croton Madag. Comoro 124. 2016, **syn. nov.** Type. Madagascar. Prov. Toliara: Antanimora, Dec 1959, *J. Bosser 13913* (holotype: P [P00133041)!; isotype: P [P00133042]!).

##### Type.

Madagascar. sin. loc., s.d., *P. Commerson s.n.* (holotype: P-JU [Catal. # 16373]!), isotype: P [P00312343]!).

##### Habit and distribution.

Shrubs; southern Madagascar (Toliara).

##### Notes.

Different sheets of the same collection were designated as types of Croton bastardii var. meridionalis and C. decaryi var. subglaber by Radcliffe-Smith (2016). Both clearly belong to the Adenophorus Group and are placed here in synonymy.

#### 98. Croton myriaster

Taxon classificationPlantaeMalpighialesEuphorbiaceae

Baker, J. Bot. 20: 268. 1882

Croton calomeris Baill., Bull. Mens. Soc. Linn. Paris 2: 860. 1890. Type. Madagascar. Madag. centr., s.d., *R. Baron 5929* (lectotype, designated here: P [P00132997]!; isolectotypes: K [K001040364]!, P [P00132998]!).
Croton myriaster var. austromadecassus Leandri, Ann. Mus. Colon. Marseille, sér. 5, 7(1): 58. 1939, as ‘*austromadecassa*’. Type. Madagascar. Prov. Fianarantsoa: Pic d’Ivohibe (Bara), 1500-2000 m, 5-11 Nov 1924, *Humbert 3198* (lectotype, designated here: P [P00224708]!; isolectotypes: P [P00338294]!, TAN [TAN000538]!). Madagascar. Prov. Fianarantsoa: Befotaka (Prov. de Farafangana), 20 Aug 1926, *R. Decary 5147* (syntype: P [P00133001]). Madagascar. Prov. Fianarantsoa: Massif d’Andringitra, 1700 m, Sep 1911, *H. Perrier de la Bâthie 9762* (syntype: P [P00133016]!). Madagascar. Prov. Fianarantsoa: haute vallée de la Rienana (Bassin du Matitanana), 1000-1400 m, 18-22 Nov 1924, *H. Humbert 3624* (syntype: P [P00133005]!). Madagascar. Prov. Toliara: Massif du Beampingaratra, vallée de la Maloto, 800-1500 m, 31 Oct-1 Nov 1938, *H. Humbert 6328* (syntypes: P [P00133008]!, US [US01050275]!). Madagascar. Prov. Toliara: Massif de l’Andohahela, 1800-1979 m, 21-22 Oct 1928, *H. Humbert 6213* (syntype: P [P00133007]!). Madagascar. Prov. Fianarantsoa: Massif de l’Ikongo (Prov. de Farafangana), 17 Oct 1926, *R. Decary 5692* (syntype: P [P00133002]!). Madagascar. Prov. Fianarantsoa: Massif de l’Ikongo (Prov. de Farafangana), 17 Oct 1926, *R. Decary 5779* (syntype: P [P00133003]!).
Croton regeneratrix var. ranomafanae Radcl.-Sm., Gen. Croton Madag. Comoro 202. 2016, **syn. nov.** Type. Madagascar. Prov. Fianarantsoa: W of Ranomafana, Parc National Ranomafana, 21°16'S, 47°28'E, 900-1100 m, 8-10 Sep 1992, *S. Malcomber & R. Rakoto 1551* (holotype: K!; isotype: MO!).

##### Type.

Madagascar. sin. loc., s.d., *R. Baron 223* (lectotype, designated here: K [K000422585]!; isolectotypes: [K000422584]!; P [P00132995]!).

##### Habit and distribution.

Trees; montane forests of Madagascar (Antananarivo, Antsiranana, Fianarantsoa, Mahajanga, Toamasina, Toliara).

#### 99. Croton nitidulus

Taxon classificationPlantaeMalpighialesEuphorbiaceae

Baker, J. Linn. Soc., Bot. 20: 253. 1883

Croton microprunus Baill., Bull. Mens. Soc. Linn. Paris 2: 861. 1890. Type. Madagascar. Prov. Toamasina: Alaotra-Mangoro Region, Didy, 14 Aug 1889, *L.D.M. Catat 1812* (holotype: P [P00132942]!).
Croton macrochlamys Baill., Bull. Mens. Soc. Linn. Paris 2: 863. 1890. Type. Madagascar. sin. loc., s.d., *R. Baron 4074* (holotype: K [K001040393]!).
Croton fuscirameus Baill., Bull. Mens. Soc. Linn. Paris 2: 927. 1891. Type. Madagascar. “Central Madagascar, received Dec 1883, *R. Baron 2988* (lectotype, designated here: K [K001040379]!; isolectotype: P [P00154398]!).
Croton nitidulus var. meridionalis Leandri, Ann. Mus. Colon. Marseille, sér. 5, 7(1): 39. 1939. Type. Madagascar. Prov. Fianarantsoa: Chaîne du Vohibory, à l’Ouest d’Ivohibe, 1000-1300 m, 1 Nov 1924, *H. Humbert 3051bis* (lectotype, designated here: P [P00133622]!; isolectotypes: G [G00446406]!), K [K001040388, K001040390]!. Madagascar. Prov. Fianarantsoa: haute vallée de l’Iantara, bassin du Manampatra, 500-800 m, 16-17 Nov 1924, *H. Humbert 3433* (syntype: P [P00133623]!).
Croton nitidulus var. parvifolius Leandri, Ann. Mus. Colon. Marseille, sér. 5, 7(1): 39. 1939, as ‘*parvifolia*’. Type. Madagascar. Prov. Antsiranana: Analamahitso (haut Bemarivo), 1000 m, Aug 1907, *H. Perrier de la Bâthie 9534* (holotype: P [P00133648]!).
Croton nitidulus var. spatulatus Leandri, Ann. Mus. Colon. Marseille, sér. 5, 7(1): 40. 1939, as ‘*spatulata*’. Type. Madagascar. Prov. Toamasina: Bassin de l’Onive, Mangoro, forêt d’Andasibe, 1000 m, Nov 1911, *H. Perrier de la Bâthie 9659* (lectotype, designated here: P [P00133626]!; isolectotypes: P [P00133627]!, P [P00133628]!).
Croton nitidulus var. fuscirameus (Baill.) Radcl.-Sm., Gen. Croton Madag. Comoro 171. 2016. Type. Based on Croton fuscirameus Baill.
Croton bracteatus subsp. manongarivensis Radcl.-Sm., Gen. Croton Madag. Comoro 137. 2016, **syn. nov.** Type. Madagascar. Prov. Antsiranana: Manongarivo Massif, above Ambodisakoana, E of Ankaramy, 14°05'S, 48°20'E, 17 Oct 1994, *G. McPherson* & *H. van der Werff 16377* (holotype: K!; isotype: MO!).
Croton nitidulus subsp. angustiglans Radcl.-Sm., Gen. Croton Madag. Comoro 170. 2016, **syn. nov.** Type. Madagascar. Prov. Toamasina: Route d’Anjiro-Moramanga (M28), Nov 1938, *G. Cours 813* (holotype: P [P00148095]!).
Croton nitidulus subsp. bekolosiensis Radcl.-Sm., Gen. Croton Madag. Comoro 170. 2016, **syn. nov.** Type. Madagascar. Prov. Antsiranana: Réserve Spéciale de Manongarivo, R. Bekolosy, 1180 m, 14°02'S, 48°18'E, 16 May 1995, *L. Gautier* & *C. Chatelain LG2681* (holotype: K!; isotype: G!).

##### Type.

Madagascar. Madag. Centr., s.d., *R. Baron 1302* (lectotype, designated here: K [K000347491]!; isolectotype: P [P00133607]!). Madagascar. Madag. Centr., s.d., *R. Baron 1349* (syntypes: K [K000347490]!, P [P00133610]!, P [P00133611]!).

##### Habit and distribution.

Shrubs; montane forests of eastern and central Madagascar (Antsiranana, Antananarivo, Fianarantsoa, Toamasina, Toliara).

#### 100. Croton nobilis

Taxon classificationPlantaeMalpighialesEuphorbiaceae

Baill., Adansonia 1: 148. 1861, as ‘nobile’

Oxydectes nobilis (Baill.) Kuntze, Revis. Gen. Pl. 2: 612. 1891. Type. Based on Croton nobilis Baill.

##### Type.

Madagascar. sin. loc.,s.d., *L.M.A. Dupetit-Thouars s.n.* (lectotype, designated here: P [P00133651]!; isolectotypes: P [P00133652]!, P [P00312353]!). Madagascar. Prov. Toliara: Anosy Region, southern slopes of Col de Maningotry, road to Ranomafana, 150 m, 16 Feb 2009, *B. van Ee, P.E. Berry, B.L. Dorsey & H. Razanatsoa 938* (epitype, designated here: MICH [MICH1514784]!; additional duplicates: MO, P, TAN).

##### Habit and distribution.

Trees; submontane moist forests of southeastern Madagascar (Toliara).

##### Notes.

The existing syntypes of *Croton nobilis* at P are all large-leaved (most around 18 × 7 cm), sterile branches. They are consistent with oversized sucker shoots that can sometimes be found on basal growth or regrowth of cut trees. The leaves on these specimens also resemble sucker leaves of *C. chrysodaphne*, which comes from eastern coastal Madagascar. However, Baillon (1861) clearly stated in the protologue that the staminate flowers of *C. nobilis* have five petals (the normal state in *Croton*), whereas *C. chyrsodaphne* usually has ten (Berry et al. 2011). Also, the pistillate sepals of *C. chrysodaphne* are described by Baillon (1861) as being oblong and entire, whereas those of *C. nobilis* are broad, squat, and reduplicate (Berry, pers. obs.). The lectotype chosen here contains a packet labeled “Flores,” with only a small fragment of an inflorescence with an irregularly flattened and straight rachis, but lacking any recognizeable floral parts that can be reconstructed. The inflorescences of *C. chrysodaphne* tend to be more slender, subterete, and curved, and this fragment does not fit that profile. Finally, Du Petit-Thouars spent six months in the Fort Dauphin area between 1792 and 1793 (Dorr 1997), so this would be consistent with the limited localities where *C. nobilis* occurs to the northwest of Fort Dauphin. We therefore conclude that the Dupetit-Thouars collections are consistent with the tree species that has subsequently been collected in moist, mid-elevation forests in the area that is now part of Andohahela National Park, named *C. nobilis*. To stabilize better this concept of *C. nobilis*, we designate here a modern epitype that has also been photographed *in situ* (viz., Fig. 1B) and sequenced for molecular phylogenetic studies (Haber et al. 2017).

#### 101. Croton noronhae

Taxon classificationPlantaeMalpighialesEuphorbiaceae

Baill., Adansonia 1: 162. 1861

Oxydectes noronhae (Baill.) Kuntze, Revis. Gen. Pl. 2: 612. 1891. Type. Based on Croton noronhae Baill.

##### Type.

Madagascar. Prov. Toamasina: Côte est, s.d., *L.A. Chapelier s.n.* (lectotype, designated here: P [P00133669]!; isolectotypes: MPU [MPU014849]!, P [P00133668]!). Madagascar. Prov. Toamasina: Foulpointe, *F. Noronha s.n.* (syntypes: G [G00018150]!, G-DC [G00311743]; ibid. loc., *W. Bojer s.n.* (syntypes: K [K001040398]!, P [P00133664]!). Madagascar. sin. loc., *L.M.A. Du Petit-Thouars s.n.* (syntypes: P [P00133680]!, P [P00133681]!).

##### Habit and distribution.

Shrubs; eastern coastal Madagascar (Fianarantsoa, Toamasina).

##### Notes.

A *Bojer s.n.* specimen at G-DC (G00311742) has a label that states “Insula Mauritius, M. Bojer 1833,” and is indicated as a type. The only Bojer specimen cited in the protologue of *Croton noronhae* (Baillon 1861) is identified as being from Foulpointe, Madagascar, and is cited from P (“h. Mus.”). Although the Geneva specimen appears to be correctly identified as *C. noronhae*, that species is not known from Mauritius. Perhaps it was from a plant native to Madagascar that was cultivated in Mauritius, as was the case with the type of *C. anisatus*.

#### 102. Croton nudatus

Taxon classificationPlantaeMalpighialesEuphorbiaceae

Baill., Adansonia 1: 168. 1861, as ‘nudatum’

Oxydectes nudata (Baill.) Kuntze, Revis. Gen. Pl. 2: 612. 1891. Type. Based on Croton nudatus Baill.
Croton boivinanus var. brevifolius Radcl.-Sm., Gen. Croton Madag. Comoro 12. 2016. Type. Madagascar. Prov. Antsiranana: Fivonandra Antsiranana II, 79 km au Sud d’Antsiranana par route RN6, et 15 km à l’Est de l’ancien chantier du Colas à Marotaolana, Campement à l’Est du village d’Ampantsona, 12°51'20"S, 49°18'10"E, 394–551 m, 3–6 Jun 1997, *O. Andrianantoanina & R. Bezara 1068* (holotype: K!; isotypes: G!, MO!, P [P00433267]!).
Croton hirsutissimus Radcl.-Sm., Gen. Croton Madag. Comoro 99. 2016. Type. Madagascar. Prov. Antsiranana: versant Est du Massif de l’Ankerana (partie S du Massif de Mafokovo, au N de Vohémar), 50-400 m, 17 Dec 1966, *Service Forestier 27363*-*SF* (holotype: P [P00154439]!).
Croton menabeensis var. furfuraceus Radcl.-Sm., Gen. Croton Madag. Comoro 101. 2016. Type. Madagascar. Prov. Mahajanga: Sofia Region, Antsohihy District, Antsatrana, Bora, 17 Jul 1970, *Service Forestier 30011* (holotype: P [P00154438]!).

##### Type.

Madagascar. Prov. Antsiranana: Diana Region, Baie de Diego-Suarès, Dec 1848, *L.H. Boivin 2659* (holotype: P [P00389498]!). Madagascar. Prov. Antsiranana: Diana Region, Antsiranana II, Orangea, road going uphill from military checkpoint at entrance to Orangea, 12°14'08"S, 49°21'40"E, 50 m, 25 Oct 2009, *B. van Ee, P.E. Berry, K.J. Wurdack, E.A. Haber & H. Razafindraibe 1081* (epitype, designated by Kainulainen et al. 2017b, pg. 374: MICH [MICH1517189]!; additional duplicate: TAN).

##### Habit and distribution.

Shrubs; northern and western Madagascar (Antsiranana, Mahajanga).

#### 103. Croton orangeae

Taxon classificationPlantaeMalpighialesEuphorbiaceae

Kainul. & P.E.Berry, Candollea 72: 394. 2017

##### Type.

Madagascar. Prov. Antsiranana: Diana Region, Ramena, Ankorikakely, Baie des Sakalava, 12°16'40"S, 49°23'01"E, 25 m, 9 Dec 2004, *J. Razafitsalama et al. 692* (holotype: MICH [MICH1517188]!; isotypes: CNARP, MO!, P [P05484901]!, TAN).

##### Habit and distribution.

Shrubs; northern Madagascar (Antsiranana).

#### 104. Croton plurispicatus

Taxon classificationPlantaeMalpighialesEuphorbiaceae

P.E.Berry, Kainul. & B.W.van Ee, Candollea 71: 342. 2016

##### Type.

Madagascar. Prov. Toamasina. Alaotra-Mangoro Region: Moramanga District, in primary moist montane forest along road heading south from highway Route Nationale 2 towards Lakato, ca 9.8 km south by line-of-sight from Rn 2, 19°03'05"S, 48°21'32"E, 1030-1060 m, 13 Aug 2015, *B. van Ee, P.E. Berry & H. Razafindraibe 2198* (holotype: MICH [MICH1513198]!; isotypes: MAPR!, MO!, MICH [MICH1513199]!, P,! TAN!).

##### Habit and distribution.

Large shrubs or small trees; montane forests of eastern Madagascar (Toamasina).

#### 105. Croton promunturii

Taxon classificationPlantaeMalpighialesEuphorbiaceae

Leandri, Adansonia, sér. 2, 10: 187. 1970

##### Type.

Madagascar. Prov. Toliara: Cap Sainte Marie, 1-150 m, 5-7 Mar 1955, *H. Humbert & R. Capuron 29220* (lectotype, designated here: P [P00312372]!; isolectotypes: K [K000422596]!, P [P00389510], P [P00389511]!).

##### Habit and distribution.

Shrubs; southern Madagascar (Toliara).

#### 106. Croton radiatus

Taxon classificationPlantaeMalpighialesEuphorbiaceae

P.E.Berry & Kainul., Candollea 71: 339. 2016 [23 Nov 2016]

Croton dasygyne Radcl.-Sm., Gen. Croton Madag. Comoro 19. 2016 [23 Dec 2016], **syn. nov.** Type. Madagascar. Prov. Toamasina: Chutes du Maningory, Lac Alaotra, s.d., *Herb. Jard. Bot. Tan. 3769 (S-39)* (holotype: P [P00131514]!).

##### Type.

Madagascar. Prov. Toamasina: Alaotra-Mangoro Region, Moramanga District, Commune Ambohibary, Fokontany Ampitambe, forêt Sahaevo, 18°49'59"S, 48°17'47"E, 1118 m, 11 Dec 2006, *J. Razanatsoa & T. Marcellin 279* (holotype: MICH [MICH1458525]!; isotypes: MO!, TAN!).

##### Habit and distribution.

Shrubs; eastern montane forests of Madagascar (Toamasina).

#### 107. Croton rakotoniainii

Taxon classificationPlantaeMalpighialesEuphorbiaceae

Leandri, Adansonia, sér. 2, 13: 295. 1973, as ‘rakotonianii’

##### Type.

Madagascar. Prov. Antsiranana: Ampitambarimena, Antalaha, 14 Mar 1955, *Réserves Naturelles de Madagascar (*) *7057* (lectotype, designated here: P [P00312373]!; isolectotypes: G [G00190674]!, K [K000422597]!, MO [sheet #04861160]), P [P00404493]!, P [P00404494]!).

##### Habit and distribution.

Shrubs; northern Madagascar (Antsiranana).

##### Notes.

The lectotype was annotated in Leandri's hand as “*Croton rakotoniainii*”, which is in accord with the collector's actual surname, so the spelling is corrected here from the variant it was published as.

#### 108. Croton regeneratrix

Taxon classificationPlantaeMalpighialesEuphorbiaceae

Leandri, Ann. Mus. Colon. Marseille, sér. 5, 7(1): 62. 1939

Croton regeneratrix var. perrierianus Leandri, Ann. Mus. Colon. Marseille, sér. 5, 7(1): 64. 1939, as ‘*perrieriensis*’. Type. Madagascar. Prov. Antananarivo: planté près d’Ambatolampy, provenant de l’Ankaratra, May 1921, *H. Perrier de la Bâthie 13769* (lectotype, designated here: P [P00133537]!; isolectotype: P [P00133538]!).

##### Type.

Madagascar. Prov. Fianarantsoa: versant nord du Pic d’Ivohibe, 1200-1400 m, 28 Sep 1926, *R. Decary 5708* (lectotype, designated here: P [P00133532]!; isolectotypes: K [K000422586]!, P [P00394468]).

##### Habit and distribution.

Trees; montane forests of central Madagascar (Antananarivo, Fianarantsoa).

#### 109. Croton sahafariensis

Taxon classificationPlantaeMalpighialesEuphorbiaceae

Kainul. & P.E.Berry, Candollea 72: 395. 2017

##### Type.

Madagascar. Prov. Antsiranana: Diana Region, Sahafary forest in the river basin, road off of Rn 6 to the E towards the “red tsingy”, 12°36'19"S, 49°26'23"E, 250 m, 26 Oct 2009, *B. van Ee, P.E. Berry, K.J. Wurdack, E.A. Haber, H. Razafindraibe & L.J. Razafitsalama 1089* (holotype: MICH [MICH1517187]!; isotypes: P!, TAN).

##### Habit and distribution.

Shrubs; northern Madagascar (Antsiranana).

#### 110. Croton sakamaliensis

Taxon classificationPlantaeMalpighialesEuphorbiaceae

Leandri, Ann. Mus. Colon. Marseille, sér. 5, 7(1): 54. 1939

Croton sakamaliensis var. microphyllus Leandri, Ann. Mus. Colon. Marseille, sér. 5, 7(1): 55. 1939, as ‘*microphylla*’. Type. Madagascar. Prov. Toliara: vallée de la Manambolo, rive gauche (Bassin du Mandrare) aux environs d’Isomono (confluent de la Sakamalio), Monts Kotriha et Isomonobe, 400-600 m, Dec.1933-Jan 1934, *H. Humbert 12826* (lectotype, designated here: P [P00133552]!; isolectotypes: K [K001040361]!, P [P00133551]!, P [P00133553]!).

##### Type.

Madagascar. Prov. Toliara: Vallée de Sakamalio, affluent de la Manambolo (Bassin du Mandrare), 500-800 m, Dec 1933, *H. Humbert 13322* (lectotype, designated here: P [P00389497]!; isolectotype: P [P00133549]!).

##### Habit and distribution.

Shrubs; southern Madagascar (Toliara).

#### 111. Croton salviformis

Taxon classificationPlantaeMalpighialesEuphorbiaceae

Baill., Bull. Mens. Soc. Linn. Paris 2: 926. 1891

Croton salviformis var. rufopunctatus Leandri, Ann. Mus. Colon. Marseille, sér. 5, 7(1): 57. 1939, as ‘*rufopunctata*’. Type. Madagascar. Prov. Toliara: Atsimo-Andrefana Region, du lac Manampetsa au delta de la Linta (côte Sud-Ouest), au N d’Itampolo, 1-10 m, 24-28 Aug 1928, *H. Humbert & C.W. Swingle 5380* (lectotype, designated here: P [P00133569]!; isolectotype: TAN [TAN000541]!). Madagascar. Prov. Toliara: Atsimo-Andrefana Region, du lac Manampetsa au delta de la Linta, 1-10 m, 17-24 Aug 1928, *H. Humbert & C.W. Swingle 5297* (syntypes: G [G00446401]!, K [K001040367]!, P [P00133567]!, P [P00133568]!).

##### Type.

Madagascar. Prov. Toliara: Anosy Region, Fort-Dauphin, s.d., *G.F. Scott-Elliot 2990* (holotype: P [P00133566]!, isotype: K [K000422583]!).

##### Habit and distribution.

Shrubs; southern Madagascar (Toliara).

#### 112. Croton scoriarum

Taxon classificationPlantaeMalpighialesEuphorbiaceae

Leandri, Adansonia, sér. 2, 12: 68. 1972

Croton bathianus var. ambatondrazakae Radcl.-Sm., Gen. Croton Madag. Comoro 114. 2016. Type. Madagascar. Prov. Toamasina: Menaloha (G-3), Distr. d’Ambatondrazaka, Nov 1937, *G. Cours 587* (holotype: P [P00127483]!).

##### Type.

Madagascar. Prov. Antsiranana-Mahajanga border: Centre, au lieu dit Analankeboka, à l’Ouest de Bealanana, 20 Nov 1966, *Service Forestier 27107-SF* (lectotype, designated by Kainulainen et al. 2017b, pg. 398: P [P00706283]!; isolectotypes: K [K000895678]!, P [P00706284]!, TEF [TEF000183]!).

##### Habit and distribution.

Shrubs or small trees; northern and central Madagascar (Antsiranana, Mahajanga, Toamasina).

#### 113. Croton stanneus

Taxon classificationPlantaeMalpighialesEuphorbiaceae

Baill., Bull. Mens. Soc. Linn. Paris 2: 850. 1890, as ‘stanneum’

Croton perrieri Leandri, Bull. Mus. Natl. Hist. Nat., sér. 2, 3: 369. 1931. Type. Madagascar. Prov. Mahajanga: Le Berizoka [Beritsoka], Oct 1897, *H. Perrier de la Bâthie 353* (lectotype, designated by Kainulainen et al. 2017b, pg. 378: P [P00404485]!; isolectotypes: K [K001040360]!, P [P00404484], P [P00404486]!, P [P00404487]!).
Croton baldauffii Leandri, Ann. Mus. Colon. Marseille, sér. 5, 7(1): 55. 1939, as ‘*baldauffi*’. Type. Madagascar. Prov. Toliara: forêt de Besomaty, entre le Fiherenana et l’Isahena (Mangoky), 750-800 m, Oct 1933, *H. Humbert 11249* (lectotype, designated by Kainulainen et al. 2017b, pg. 378: P [P00301487]!; isolectotype: P [P00127468]!).
Croton ikopae Leandri, Ann. Mus. Colon. Marseille, sér. 5, 7(1): 83. 1939. Type. Madagascar. Prov. Antananarivo: Analamanga Region, vallée de l’Ikopa, au NW d’Ankazobe, 15 Mar 1930, *R. Decary 7554* (lectotype, designated by Kainulainen et al. 2017b, pg. 378: P [P00154394]!; isolectotypes: K [K001040362]!, P [P00154395]!, P [P00154396]!, P [P00154397]!).
Croton crocodilorum var. platyaster Radcl.-Sm., Gen. Croton Madag. Comoro 112. 2016. Type. Madagascar. Prov. Toliara: forêt du Zombitsy, près de Sakaraha, Mar 1960, *M. Keraudren 510* (holotype: P [P00154485]!).
Croton parvifructus Radcl.-Sm., Gen. Croton Madag. Comoro 122. 2016. Type. Madagascar. Prov. Toliara: Forêt de Zombitsy, au NE de Sakaraha (150 km NE Tuléar), 600-800 m, 2 Nov 1960, *J. Leandri & J. Ratoto 3605* (holotype: P [P00132992]!).
Croton stanneus var. hirsutus Radcl.-Sm., Gen. Croton Madag. Comoro 64. 2016. Type. Madagascar. Prov. Fianarantsoa. Andranovola, near Lomanosiny, Antambohobe Canton, Ivohibe District, 13 Aug 1967, *Service Forestier 26381* (holotype: P [P00133592]!).

##### Type.

Madagascar. “Madag. centr.”, received Nov 1885, *R. Baron 3382* (holotype: K [K001040368]!, isotype: P [P00133580]!).

##### Habit and distribution.

Shrubs to small trees; across most of Madagascar (Antananarivo, Antsiranana, Fianarantosa, Mahajanga, Toamasina, Toliara).

##### Notes.

See Kainulainen et al. (2017b) for a discussion on the morphological variation and recircumscription of this widespread species.

#### 114. Croton submetallicus

Taxon classificationPlantaeMalpighialesEuphorbiaceae

Baill., Bull. Mens. Soc. Linn. Paris 2: 966. 1891, as ‘submetallicum’

Croton chapelieri var. longepetiolata Radcl.-Sm., Gen. Croton Madag. Comoro 152. 2016, **syn. nov.** Type. Madagascar. Prov. Toamasina: W of , Réserve Naturelle Intégrale Zahamena, Amboditamenaka Forest, 17°44'S, 49°00'E, 15–20 Sep 1993, *S. Malcomber B. Rasolondraibe, L.M. Randrianjanaka & J.P. Abraham 2511* (holotype: K!; isotypes: MO [MO-2990285]!, P [P00433109]!).
Croton fianarantsoae var. obovalifolius Radcl.-Sm., Gen. Croton Madag. Comoro 155. 2016, **syn. nov.** Type. Madagascar. Prov. Fianarantsoa: W of Ranomafana, 21°16'S, 47°28'E, 8–10 Sep 1992, *S. Malcomber* & *R. Rakoto 1550* (holotype: K; isotypes: MO, P [P00422460]!).
Croton nitidulus var. acuminatus Radcl.-Sm., Gen. Croton Madag. Comoro 169. 2016, **syn. nov.** Type. Madagascar. Prov. Toamasina: Itinéraire de Didy à Brickaville (forêt orientale), reçu Apr 1954, *G. Cours 4871* (lectotype, designated here: P [P00133641]!; isolectotypes: P [P00133639]!; P [P00133640]!; P [P00133642]!).
Croton nitidulus var. cinereus Radcl.-Sm., Gen. Croton Madag. Comoro 170. 2016, **syn. nov.** Type. Madagascar. Prov. Toamasina: Ambatovy, 18°51'34"S, 48°18'25"E, 3 Mar 1997, *P. Rakotomalaza et al. 1217* (holotype: K!; isotype: MO!).
Croton nitidulus var. hypopoliotes Radcl.-Sm., Gen. Croton Madag. Comoro 172. 2016, **syn. nov.** Type. Madagascar. “Central Madagascar”, received Dec 1883, *R. Baron s.n*. (holotype: K!).
Croton nitidulus var. macrophyllus Radcl.-Sm., Gen. Croton Madag. Comoro 172. 2016, **syn. nov.** Type. Madagascar. Prov. Toamasina: Itinéraire de Didy à Brickaville (forêt orientale), s.d., *G. Cours 4660* (lectoype, designated here: P [P00133616]!; isolectotypes: P [P00133615]!, P [P00133617]!).
Croton submetallicus var. tomentosus Radcl.-Sm., Gen. Croton Madag. Comoro 58. 2016, **syn. nov.** Type. Madagascar. Prov. Toamasina: forêt de l’Analamazaotra, reçu 3 Dec 1934, *E. Ursch 21* (holotype: P [P00133354]!).

##### Type.

Madagascar. sin. loc., s.d., *R. Baron 5286* (lectotype, designated here: K [K000422582]!; isolectotypes: K [K000422581]!, P [P00133327]!). Madagascar. Prov. Toamasina: Alaotra-Mangoro Region. Forest W of Eulophiella Hotel, south of Rn 2 and Andasibe, 18°59'08"S, 48°25'55"E, 900 m, 15 Aug 2015, *B. van Ee, P.E. Berry & H. Razafindraibe 2222* (epitype, designated here: MICH [MICH1514786]!; additional duplicates: G, K, MO, P, TAN, US).

##### Habit and distribution.

Shrubs or trees; eastern montane forests of Madagascar (Fianarantsoa, Toamasina).

##### Notes.

The type of *Croton submetallicus* is deficient for characterizing this species properly; it lacks pistillate flowers, which are very diagnostic. Therefore we are designating an epitype that is well distributed, has photographs in Tropicos®, and is also sampled molecularly. The only other described species name that could be applied here is *C. macrochlamys*, but it has a very poor type specimen, and we are treating it here as a synonym of *C. nitidulus* because of its smaller leaf size.

For Croton nitidulus var. acuminatus, Radcliffe-Smith (2016) designated *Cours 4871* at P as the holotype, but there are four nearly identical specimens of this collection at P, so we selected one of them here as lectotype. Likewise, for C. nitidulus var. macrophyllus, Radcliffe-Smith (2016) designated *Cours 4660* at P as the holotype, but since there are three duplicates of that collection there, we selected one of them as the lectotype.

#### 115. Croton tanalorum

Taxon classificationPlantaeMalpighialesEuphorbiaceae

Leandri, Ann. Mus. Colon. Marseille, sér. 5, 7(1): 38. 1939

##### Type.

Madagascar. Prov. Fianarantsoa: Ifandana (Prov. de Farafangana), 8 Sep 1926, *R. Decary 5070* (holotype: P [P00133355]!; isotypes: K [K001044840]!, TAN [TAN000543]!).

##### Habit and distribution.

Shrubs; eastern montane forests of Madagascar (Fianarantsoa).

#### 116. Croton tardeflorens

Taxon classificationPlantaeMalpighialesEuphorbiaceae

Leandri, Ann. Mus. Colon. Marseille, sér. 5, 7(1): 50. 1939

##### Type.

Madagascar. Prov. Mahajanga: Ménabé, Tsingy du Bemaraha (9^e^ Réserve), Feb 1933, *J. Leandri 941* (lectotype, designated here: P [P00133358]!; isolectotypes: K [K001040355]!, P [P00133359]!, P [P00133360]!). Madagascar. Prov. Mahajanga: Boïna, bords du Jabohazo près du Mont Tsitondroina, Dec 1902, *H. Perrier de la Bâthie 9799* (syntypes: P [P00133361]!, P [P00133362]!, P [P00133363]!).

##### Habit and distribution.

Shrubs; western Madagascar (Mahajanga).

#### 117. Croton thouarsianus

Taxon classificationPlantaeMalpighialesEuphorbiaceae

Baill., Adansonia 1: 167. 1861, as ‘thuarsianum’

Oxydectes thouarsiana (Baill.) Kuntze, Revis. Gen. Pl. 2: 613. 1891. Type. Based on Croton thouarsianus Baill.
Croton mocquerysii var. meridionalis Radcl.-Sm. Radcl.-Sm., Gen. Croton Madag. Comoro 160. 2016, **syn. nov.** Type. Madagascar. Toliara Province: Réserve Naturelle Intégrale # 11, Andohahela, à vicinité d’Eminiminy, 24°40'S, 46°48'E, 13–25 Jan 1993, *B. Randriamampionona* 77 (holotype: K!; isotypes: MO!, P [P00433050]!).
Croton thouarsianus var. robustior Radcl.-Sm., Gen. Croton Madag. Comoro 183. 2016, **syn. nov.** Type. Madagascar. Toliara Province: Préfecture de Fort Dauphin, Forêt d’Analalava, Manantenina, côte Est, 1 Nov 1990, *N. Dumetz 1378* (holotype: K!; isotype: MO!).

##### Type.

Madagascar: sin. loc., s.d., *L.M.A. Du Petit-Thouars s.n.* (lectotype, designated here: P [P00389504]!; isolectotype: P [P00389505]!).

##### Habit and distribution.

Shrubs; southeastern Madagascar (Toliara).

#### 118. Croton tiliifolius

Taxon classificationPlantaeMalpighialesEuphorbiaceae

Lam., Encycl. 2: 206. 1786, as ‘tiliaefolium’

Oxydectes tiliifolia (Lam.) Kuntze, Revis. Gen. Pl. 2: 613. 1891. Type. Based on Croton tiliifolius Lam.

##### Type.

Mauritius. collector unknown [likely *P. Commerson s.n.*] (holotype: P-LA [P00382046]!). Mauritius. s.d., *P. Commerson s.n.* (likely original material or isotype: P [P00121778]!).

##### Habit and distribution.

Trees; Mauritius.

##### Notes.

Lamarck (1786) stated in his protologue that *Croton tiliifolius* is found on the islands of Mauritius and Réunion (Isles de France et de Bourbon), but we have no evidence to support its native presence on Réunion. Lamarck (1786) cited a Commerson herbarium specimen from Mauritius, and that corresponds well to the specimen in the general herbarium at P [P00121778], whereas the specimen in the Lamarck Herbarium does not list a collector or locality.

#### 119. Croton toliarensis

Taxon classificationPlantaeMalpighialesEuphorbiaceae

B.W.van Ee & Kainul. nom. nov.

urn:lsid:ipni.org:names:77167303-1

Croton tranomarensis var. rosmarinifolius Radcl.-Sm., Gen. Croton Madag. Comoro 196. 2016. Type. Madagascar. Prov. Toliara: 38 km SW of Ampanihy, on road to Androka, 24°50'S, 44°25'E, 5 Feb 1990, *P.B. Phillipson J.-N. Labat, D. Du Puy & B. Du Puy 3434* (holotype: K! isotypes: DAV!, G!, MO!, P [P00433298]!).

##### Type.

Based on Croton tranomarensis var. rosmarinifolius Radcl.-Sm.

##### Habit and distribution.

Shrubs; southern Madagascar (Toliara).

##### Notes.

The specific epithet “*rosmarinifolius*” is previously occupied in *Croton* (*Croton rosmarinifolius* Salisb. (1796), which is itself an illegitimate replacement name for *C. cascarilla* (L.) L.), hence the new name coined here. In both this species as well as the similar *C. chlaenacicomes*, the leaf margins often become revolute when the plant is undergoing drought stress; that is most likely what caused the strongly inrolled margins on the type of C. tranomarensis var. rosmarinifolius.

#### 120. Croton trichotomus

Taxon classificationPlantaeMalpighialesEuphorbiaceae

Geiseler, Croton. Monogr.: 50. 1807

Croton pulchellus Baill., Adansonia 1: 161. 1861, as ‘*pulchellum*’. Type. Madagascar. sin. loc., s.d., *J. Martin s.n.* (holotype: G [G00446399]!; isotype: P [P00133530]!).
Oxydectes pulchella (Baill.) Kuntze, Revis. Gen. Pl. 2: 612. 1891. Type. Based on Croton pulchellus Baill.
Oxydectes trichotoma (Geiseler) Kuntze, Revis. Gen. Pl. 2: 613. 1891. Type. Based on Croton trichotomus Geiseler
Croton trichotomus var. pulchellus (Baill.) Leandri, Ann. Mus. Colon. Marseille, sér. 5, 7(1): 50. 1939. Type. Based on Croton pulchellus Baill.
Croton remotiflorus Radcl.-Sm., Gen. Croton Madag. Comoro 48. 2016, **syn. nov.** Type. Madagascar. Prov. Toliara: Réserve Intégrale # 11, Andohahela, vicinity of Eminiminy, 24°40'S, 46°48'E, 4-24 May 1993, *B. Randriamampionona 385* (holotype: K!; isotypes: DAV!, G [G00414974]!, MICH!, MO!).
Croton antanosiensis var. ambohibyi Leandri ex Radcl.-Sm., Gen. Croton Madag. Comoro 42. 2016, **syn. nov.** Type. Madagascar. Prov. Antananarivo: Mont Ambohiby, SE de Tsiroanomandidy, 1600 m, 11-16 Nov 1952, *J. Leandri*, *R. Capuron* & *A. Razafindrakoto 1775* (lectotype, designated here: P [P00154305]!; isolectotypes: P [P00154302]!, P [P00154303]!, P [P00154304]!).
Croton isomonensis var. microcarpus Radcl.-Sm., Gen. Croton Madag. Comoro 50. 2016, **syn. nov.** Type. Madagascar. Prov. Fianarantsoa: Antambohobe Canton, Ivohibe Distr., 6 Mar 1962, Réserves Naturelles 12150-Rn (holotype: P [P00154428]!).

##### Type.

Madagascar. “*Madagascar f. maut., N^o^ 38*” (lectotype, designated here: P-LA [P00382065]!).

##### Habit and distribution.

Shrubs; mainly an eastern coastal species of Madagascar in Toamasina and Toliara Provinces, as far south as the Fort Dauphin area, but also recorded from Antananarivo and Fianarantsoa Provinces in upland forests (as C. antanosiensis var. ambohibyi and C. isomonensis var. microcarpus).

##### Notes.

In the protologue of *Croton trichotomus*, Geiseler (1807) listed two different elements, *C. trichotomus* from Madagascar and *C. punctatus* from the Caribbean. We choose here as lectotype the collection in P-LA that Leandri (1939) attributes to P. Commerson. There is no sheet in P-JU that matches the plant of *C. trichotomus* on the P-LA sheet, but P-JU Catal. 16347 is a mixed collection that bears a label on the left-hand specimen [P00674048] that states, possibly in Geiseler's hand, [Croton] “punctatum Jacq.” followed below by “trichotomum Geiseler, Crot. Monogr.” However, this is a completely different plant from the one represented on the P-LA sheet, instead belonging to the Caribbean *C. flavens* L.

In our view, *Croton trichotomus* is primarily a littoral species of the eastern coast, but it also occurs in a number of more inland and higher elevation situations, which is an exception among Malagasy *Croton* species.

For Croton antanosiensis var. ambohibyi, Radcliffe-Smith (2016) designated *Leandri et al. 1775* at P as the holotype, but since there are four sheets of this number at P, we select one of them as lectotype.

#### 121. Croton tsiampiensis

Taxon classificationPlantaeMalpighialesEuphorbiaceae

Leandri, Ann. Mus. Colon. Marseille, sér. 5, 7(1): 79. 1939

Croton tsiampiensis var. ankaranensis Radcl.-Sm., Gen. Croton Madag. Comoro 110. 2016. Type. Madagascar. Prov. Antsiranana: Diana Region, Massif de l’Ankarana, 4 Nov 1990, *M. Bardot-Vaucoulon 224* (holotype: P00123707!).
Croton tsiampiensis var. macrophyllus Radcl.-Sm., Gen. Croton Madag. Comoro 110. 2016. Type. Madagascar. Prov. Mahajanga: Reserve Naturelle Bemaraha, Ambodiriana, ca. 9 km E of Antsalova, 18°39'S, 44°43'E, 100–125 m, 13–15 Dec 1990, *L. Gillespie 4139* (holotype: K!; isotype: MO).
Croton tsiampiensis var. microphyllus Radcl.-Sm., Gen. Croton Madag. Comoro 111. 2016. Type. Madagascar. Prov. Antsiranana: Diana Region, P.K. 10 de la route Diego Suarez-Orangea, 13 Dec 1963, *Service Forestier 22956* (holotype: P00123706!).

##### Type.

Madagascar. Prov. Mahajanga: Tsiampihy, près de l’embouchure de la , 15 Oct 1932, *J. Leandri 311* (lectotype, designated by Kainulainen et al. 2017b, pg. 399: P [P00389521]!; isolectotype: P [P00133302]!).

##### Habit and distribution.

Shrubs; northern and western Madagascar (Antsiranana, Mahajanga).

#### 122. Croton ustulatus

Taxon classificationPlantaeMalpighialesEuphorbiaceae

Radcl.-Sm., Gen. Croton Madag. Comoro 28. 2016

##### Type.

Madagascar. Prov. Toliara: 8-16 km E of Tuléar (Toliara) on road to Tananarive (Antananarivo), 50 m, 7 Feb 1975, *T. Croat 30998* (holotype: K!; isotypes: MO!, P [P00433216]!).

##### Habit and distribution.

Shrubs; southwestern Madagascar (Toliara).

#### 123. Croton vaughanii

Taxon classificationPlantaeMalpighialesEuphorbiaceae

Croizat, Trop. Woods 77: 14. 1944

##### Type.

Mauritius. Perrier, near the Mare aux Vacoas, 500 m, 12 May 1938, *R.E. Vaughan MAU accession number 863* (holotype: A [A00047560]; isotype: WIS [WIS00000605MAD]). Mauritius. [in flower] 14 Feb 1934, *R.E. Vaughan MAU accession number 863* (paratype: K [K000422600]!). Mauritius. [in fruit] 1 Apr 1934, *R.E. Vaughan MAU accession number 863* (paratype: K [K000422600]!), MAU, MAU).

##### Habit and distribution.

Shrubs; Mauritius.

##### Notes.

Croizat (1944) referenced collector information for characteristics of *C. vaughanii* that are not observable on the holotype at A (“*fide collectoris*”), and the holotype is accompanied by a copy of field notes referencing that the species drops its leaves in November and December, and that flowering takes place immediately afterward. According to information in the Mauritius Herbarium (MAU) database, in this case “863” is the accession number rather than the collection number. Given this, the sheets at K and MAU with the accession number 863 are not a part of the same gathering as the holotype at A and therefore are not syntypes. Even though Croizat (1944) did not explicitly refer to the “*Vaughan 863*” collections at K and MAU, we interpret them as being paratypes. Furthermore, the sheet at K labeled with the barcode K000422600 represents material from two gatherings on different dates and should be considered two distinct specimens.

*Croton vaughanii* is listed by the IUCN as Critically Endangered (Strahm 1998), and is known from only a single individual according to information on the label of *Haevermans et al. 558* (P [P00696110]), making it one of the rarest of all *Croton* species.

#### 124. Croton vatomandrensis

Taxon classificationPlantaeMalpighialesEuphorbiaceae

Leandri, Ann. Mus. Colon. Marseille, sér. 5, 7(1): 71. 1939

##### Type.

Madagascar. Prov. Toamasina: environs de Vatomandry, près de lagunes, Nov 1921, *H. Perrier de la Bâthie 14084* (lectotype, designated by Kainulainen et al. 2017a, pg. 39: P [P00248926]!; isolectotypes: P [P00154409]!, P [P00154410]!).

##### Habit and distribution.

Shrubs or small trees; eastern littoral forests of Madagascar (Fianarantsoa, Toamasina).

### Incertae Sedis

This section includes 22 taxa described by Radcliffe-Smith (2016) and three by Leandri (1939) for which we have not yet been able to determine if they are worthy of recognition; they will require further evaluation to decide whether they should be considered synonyms of earlier-described taxa, or if in some cases they are actually distinct species rather than mere varieties of existing species.

#### Croton ankarensis var. ankarafantsikae

Taxon classificationPlantaeMalpighialesEuphorbiaceae

Radcl.-Sm., Gen. Croton Madag. Comoro 139. 2016

##### Type.

Madagascar. Prov. Mahajanga: Ampijoroa, Ankarafantsika, 26 Oct 1970, *A. Richard 476* (holotype: K!).

##### Notes.

It is uncertain what this variety is, but probably does not belong to *Croton ankarensis*.

#### Croton bastardii var. bongolavae

Taxon classificationPlantaeMalpighialesEuphorbiaceae

Radcl.-Sm., Gen. Croton Madag. Comoro 204. 2016

##### Type.

Madagascar. Prov. Mahajanga: Bongolava Hills, Port Bergé, 15°40'S, 47°30'E, 23 Nov 1987, *E. Bisset 38* (holotype: K!).

##### Notes.

It is uncertain what this is, but it is probably not a member of *Croton bastardii*.

#### Croton bemaranus var. parvistipulatus

Taxon classificationPlantaeMalpighialesEuphorbiaceae

Radcl.-Sm., Gen. Croton Madag. Comoro 209. 2016

##### Type.

Madagascar. Prov. Toliara: 45 km N of Tulear on Morombe road E of junction to Manombo, 23°01'S, 43°36'E, 14 Dec 1988, *P.B. Phillipson 2884* (holotype: K!; isotype: MO!).

##### Notes.

*Croton bemaranus* is restricted to tsingy habitats in Antsiranana and Mahajanga Provinces and has entire leaves and elongate stipules. This variety does not fit the species at all and requires further study to determine its affinities.

#### Croton betiokensis var. haplostylis

Taxon classificationPlantaeMalpighialesEuphorbiaceae

Radcl.-Sm., Gen. Croton Madag. Comoro 102. 2016

##### Type.

Madagascar. Prov. Fianarantosoa: Ihosy-Ivohibe, 21 Dec 1965, *J. Peltier* & *M. Peltier 5534* (holotype: P [P00154466]!).

##### Notes.

This is a very scrappy specimen, making it difficult to determine what it may be. It does not appear to belong to *Croton betiokensis*, which is confined to southern Toliara Province.

#### Croton catatii var. schizolepis

Taxon classificationPlantaeMalpighialesEuphorbiaceae

Radcl.-Sm., Gen. Croton Madag. Comoro 44. 2016

##### Type.

Madagascar. Prov. Toliara: 45 km NE of Morondava on Beroboka road, 20°03'S, 44°37'E, 7/8 Dec 1990, *L. Gillespie 4117* (holotype: K!; isotype: MO!).

##### Notes.

This variety has much smaller leaves than typical *Croton catatii* and is only a small shrub, so it is not clear yet whether it fits within another species or of it may represent an undescribed species.

#### Croton daphniphylloides var. stellatipilus

Taxon classificationPlantaeMalpighialesEuphorbiaceae

Radcl.-Sm., Gen. Croton Madag. Comoro 165. 2016

##### Type.

Madagascar. Prov. Antsiranana: Marojejy, N of Mandena, 14°29'S, 49°49'E, 22 Apr 1993, *A. Randrianasolo 322* (holotype: K!; isotype MO!).

##### Notes.

It is unlikely this belongs to *C. chapelieri* (in which *C. daphniphylloides* is treated here as a synonym); it occurs in the Marojejy Reserve at 500 m elevation and is a 8 m tall tree, whereas *C. chapelieri* is confined to the eastern littoral zone near sea level. It could also be a northern form of *C. submetallicus*, with more acute leaves and lacking the submetallic leaf undersides of that species.

#### Croton daphniphylloides var. triplinervius

Taxon classificationPlantaeMalpighialesEuphorbiaceae

Radcl.-Sm., Gen. Croton Madag. Comoro 165. 2016

##### Type.

Madagascar. Prov. Antsiranana: Mt. Anjenabe, vallée inférieure de l’Androranga, affluent de la Bemarivo aux environs d’ Antongondriha, 3-7 Nov 1950, *H. Humbert* & *R. Capuron 24021* (holotype: K!; isotypes: MO!, P [P00433396]!).

##### Notes.

Radcliffe-Smith (2016) erroneously cited the type collection number as *24041*, which is a *Wielandia*. This may be the same entity as *C. daphnyphylloides var. stellatipilus*.

#### Croton elaeagni var. argyrocarpos

Taxon classificationPlantaeMalpighialesEuphorbiaceae

Radcl.-Sm., Gen. Croton Madag. Comoro 90. 2016

##### Type.

Madagascar. [Prov. Antananarivo?]: environs d’Ambohikely, s.d. [1952-1955], *J. Dequaire 27466* (holotype: P [P00154267]!).

##### Notes.

This does not appear to belong to *C. elaeagni*, and its placement is uncertain.

#### Croton elaeagni var. brevipedicellatus

Taxon classificationPlantaeMalpighialesEuphorbiaceae

Radcl.-Sm., Gen. Croton Madag. Comoro 90. 2016

##### Type.

Madagascar. [Prov. Antananarivo?]: environs d’Ambohikely, s.d. [1952-1955], *J. Dequaire 27460* (holotype: P [P00154282]!).

##### Notes.

This may be the same as the preceding variety.

#### Croton fianarantsoae var. petiolaris

Taxon classificationPlantaeMalpighialesEuphorbiaceae

Radcl.-Sm., Gen. Croton Madag. Comoro 155. 2016

##### Type.

Madagascar. Prov. Antsiranana: Andapa, Andrakata, Analamboahangy Marojejy RNI, aux environs de Manenobasy, 14°35'16"S, 49°41'20"E, 1171 m, 18-24 Jan 1995, *F. Rasoavimbahoaka 488* (holotype: MO; isotypes: P [P00433385]!, TAN).

##### Notes.

This may correspond to *Croton submetallicus*, but the leaves are acuminate and not submetallic on the undersides, and it occurs well north of the known distribution of that species.

#### Croton hovarum var. hirsutifructus

Taxon classificationPlantaeMalpighialesEuphorbiaceae

Radcl.-Sm., Gen. Croton Madag. Comoro 178. 2016

##### Type.

Madagascar. Prov. Toamasina: Valoala Triangulation Point, 17°25'S, 48°15'E, 6 Jan 1945, *A.M. Homolle 2193* (holotype: P [P00133178]!).

##### Notes.

This is a fruiting specimen with small pistillate sepals and rusty stellate pubescence that is probably not *C. hovarum*, but it cannot be placed yet in another species.

#### Croton ivohibensis var. integrifolius

Taxon classificationPlantaeMalpighialesEuphorbiaceae

Radcl.-Sm., Gen. Croton Madag. Comoro 149. 2016

##### Type.

Madagascar. Prov. Toliara: bassin de réception de la Mananara, affluent du Mandrare, pentes occidentales des montagnes entre l’Andohahela et l’Elakelaka, Mt Apiky au dessus de Mahamava, 800-900 m, Jan-Feb 1934, *H. Humbert 13821* (lectotype, designated here: P [P00133268]!; isolectotypes: P [P00133269]!, P [P00133270]!).

##### Notes.

This is likely an undescribed species from the lower end of evergreen forests in southeastern Toliara Province. The pistillate flowers have a very long pedicel, and the calyx is very well developed. It is likely the same as C. nitidulus var. eglandulosus and C. *thouarsianus var. angustifolius* (see below).

#### Croton ivohibensis var. macrocalyx

Taxon classificationPlantaeMalpighialesEuphorbiaceae

Radcl.-Sm., Gen. Croton Madag. Comoro 150. 2016

##### Type.

Madagascar. sin. loc., received Jan 1892, *R. Baron 6134* (holotype: K!; isotype: P [P00154509]!).

##### Notes.

This could be a form of *Croton hovarum*, but the leaves seem too coarsely serrate for that species, and not knowing where the specimen came from, hinders our ability to place it.

#### Croton ivohibensis var. polygynus

Taxon classificationPlantaeMalpighialesEuphorbiaceae

Radcl.-Sm., Gen. Croton Madag. Comoro 150. 2016

##### Type.

Madagascar. Prov. Toamasina: Lac Alaotra, *Herb. Jard. Bot. Tan. 3777* (holotype: P [P00133298]!).

##### Notes.

This is an interesting specimen that bears some resemblance to *Croton tanalorum*. Additional material is needed to assess this further. Another specimen of it is *Cours 631* (P [P00133297]).

#### Croton ivohibensis var. verticillatus

Taxon classificationPlantaeMalpighialesEuphorbiaceae

Radcl.-Sm., Gen. Croton Madag. Comoro 151. 2016

##### Type.

Madagascar. Prov. Fianarantsoa: Reserve Speciale de Pic d’ Ivohibe, Marovitsika Forest, 22°28'49"S, 46°56'49"E, 850 m, 17 Oct 2000, *P. Hoffmann et al. 223* (holotype: K; isotype: MO!).

##### Notes.

This does not appear to belong to *Croton hovarum* (where we include *C. ivohibensis*), and it needs further study to determine its affinities.

#### Croton lapiazicola var. longibracteatus

Taxon classificationPlantaeMalpighialesEuphorbiaceae

Leandri, Ann. Mus. Colon. Marseille, sér. 5, 7(1): 42. 1939, as ‘longibracteata’

##### Type.

Madagascar. Prov. Mahajanga: bassin supérieur du Bemarivo (), 800 m, Sep 1907, *H. Perrier de la Bâthie 9550* (lectotype, designated here: P [P00133404]!; isolectotypes: P [P00133403]!, P [P00133405]!).

##### Notes.

The type is somewhat similar to *Croton ericius*, but the leaves are much narrower and it is less hirsute than that species.

#### Croton manampetsae var. lepidotus

Taxon classificationPlantaeMalpighialesEuphorbiaceae

Radcl.-Sm., Gen. Croton Madag. Comoro 27. 2016

##### Type.

Madagascar. Prov. Toliara: environs de Tuléar (Toliara), 30 km Tulear-Tananarive, Mar 1960, *M. Keraudren 541* (holotype: P [P00133295]!).

##### Notes.

This variety differ from *Croton manampetsae* in that the leaves are mostly alternate (vs. opposite) and in the lepidote indumentum on the abaxial side of the leaves (vs. stellate trichomes). It will likely be described as a distinct species.

#### Croton meridionalis var. latifolius

Taxon classificationPlantaeMalpighialesEuphorbiaceae

Radcl.-Sm., Gen. Croton Madag. Comoro 193. 2016

##### Type.

Madagascar. Prov. Toliara: haute vallée du Mandrare près d’Andetra (Andotsy), 26 Nov 1928, *H. Humbert 6849* (holotype: G!; isotypes: K, P [P00418562]!).

##### Notes.

This plant is similar to *C. meridionalis*, except that it has considerably wider leaves that are silvery-lepidote on the lower surface and turn dark blackish green on the upper surface when dried. Further study is needed to determine if it should be recognized as a distinct species.

#### Croton meridionalis var. stipularis

Taxon classificationPlantaeMalpighialesEuphorbiaceae

Radcl.-Sm., Gen. Croton Madag. Comoro 194. 2016

##### Type.

Madagascar. Prov. Toliara: 45 km N of Tulear (Toliara) on road to Morombe E of junction to Manombo, 23°01'S, 43°36'E, 14 Dec 1988, *P.B. Phillipson 2885* (holotype: K!; isotypes: DAV!, MO!, P [P00123724]!).

##### Notes.

This is likely the same as the preceeding variety.

#### Croton nitidulus var. eglandulosus

Taxon classificationPlantaeMalpighialesEuphorbiaceae

Radcl.-Sm., Gen. Croton Madag. Comoro 171. 2016

##### Type.

Madagascar. Prov. Toliara: bassin de réception de la Mananara, affluent du Mandrare, entre Andohahela et Elakelaka, Mt. Apiky au-dessus de Mahamavo, Jan-Feb 1934, *H. Humbert 13848* (lectotype, designated here: P [P00133646]!; isolectotypes: G!, P [P00133647]!).

##### Notes.

This is probably the same undescribed species as Croton ivohibensis var. integrifolius and C. *thouarsianus var. angustifolius*.

#### Croton rubricapitirupis var. macrophyllus

Taxon classificationPlantaeMalpighialesEuphorbiaceae

Radcl.-Sm., Gen. Croton Madag. Comoro 158. 2016

##### Type.

Madagascar. Prov. Antsiranana: Andapa, pentes occidentales du Massif de Marojejy (Nord- Est), Bassin de la Lokoho, a l’Est d’ Ambalamanasy II, 500-800 m, 28 Nov-6 Dec 1948, *H. Humbert & R. Capuron 22126* (holotype: K; isotypes: P [P00433398]!, P [P00433399]!).

##### Notes.

The type of this variety is a large-leaved shrub with a very large, foliaceous pistillate calyx on a long pedicel. It may represent an undescribed species.

#### Croton thouarsianus var. angustifolius

Taxon classificationPlantaeMalpighialesEuphorbiaceae

Radcl.-Sm., Gen. Croton Madag. Comoro 183. 2016

##### Type.

Madagascar. Toliara Province: NW of Tolanaro, Reserve Naturelle Intégrale # 11 (Andohahela), NW of Eminiminy, beside River Itrotroky, 24°38'S, 46°46'E, 6–13 Feb 1993, *S. Malcomber et al. 2159* (holotype: K!; isotypes: G!, MO).

##### Notes.

This is probably the same undescribed species as Croton ivohibensis var. integrifolius and C. nitidulus var. eglandulosus.

#### Croton thouarsianus var. longifolius

Taxon classificationPlantaeMalpighialesEuphorbiaceae

Radcl.-Sm., Gen. Croton Madag. Comoro 183. 2016

##### Type.

Madagascar. Prov. Fianarantsoa: Réserve Speciale de Manombo, 23°01'43"S, 47°43'51"E, 26 Oct 2000, *P. Hoffmann et al. 270A* (holotype: K!; isotypes: BR, G, MO).

##### Notes.

This is probably an undescribed species from the inland area of the Manombo Reserve in Fianarantsoa Province close to the coast, but not related to *Croton thouarsianus*.

#### Croton tranomarensis var. isomoni

Taxon classificationPlantaeMalpighialesEuphorbiaceae

Leandri, Ann. Mus. Colon. Marseille, sér. 5, 7(1): 61. 1939

##### Type.

Madagascar. Prov. Toliara: Anosy Region, vallée de la Manombolo, rive droite (basin du Mandrare) aux environs d’Isomono (confluent de la Sakamalio), mont Morahariva, 1000-1200 m, Oct 1933, *H. Humbert 13105* (lectotype, designated here: P [P00133393]!; isolectotypes: G [G00446403]!, P [P00133391]!, P [P00133392]!; K [K001040390]!, TAN [TAN000546]).

##### Notes.

The leaves of these specimens are too small to conform to *Croton meridionalis*, and Radcliffe-Smith (2016) also expressed doubt that it belonged there but did not know where else to place it.

#### Croton tranomarensis var. minor

Taxon classificationPlantaeMalpighialesEuphorbiaceae

Leandri, Ann. Mus. Colon. Marseille, sér. 5, 7(1): 61. 1939

##### Type.

Madagascar. Prov. Toliara: Anosy Region, Ambovombe, Behara, 9 Jan 1931, *R. Decary 8370* (holotype: P [P00133390]!; isotypes: K [K001040391]!, TAN [TAN000545]).

##### Notes.

The leaves of the type of this variety are too small to conform to *Croton meridionalis* (for which *C. tranomarensis* is a synonym). Radcliffe-Smith (2016) expressed doubt that it belonged here, but he did not know where else to place it other than to suggest that it may be close to *C. salviformis* (presumably because of the small leaves and venose pistillate sepals). However, the holotype appears to have entirely pistillate inflorescences, suggesting that the plants may be dioecious. If so, it may correspond to an apparently undescribed species that we have also found south of Ranopiso at Mt. Vohitsiandriana in Toliara Province.

### “Names” not validly published

*Croton ambovombensis var. lepidotus* Radcl.-Sm., Gen. Croton Madag. Comoro 192. 2016, nom. inval. (Art. 40.7). This designation is not validly published because Radcliffe-Smith (2016) cited both the G specimen and the P specimen (based on the collection: Madagascar. Prov. Toliara: 6 km N de Faux Cap, Apr 1972, *P. Morat 3952* (G, K, P [P00154467]!) as the holotype, in contravention to Article 40.7 of the ICN (McNeill et al. 2012). Had it been validly published, it would have been placed in the Incertae Sedis section. This collection is of a linear-leaved plant with long virgate stems that lacks the orangeish stellate indumentum and strongly divaricate branching pattern of *C. ambovombensis*.

Croton arenicola f. pubescens Leandri, Ann. Mus. Colon. Marseille, sér. 5, 7(1): 58. 1939, nom. inval. Protologue lacking Latin description or diagnosis (McNeill et al. 2012; Art. 39.1). = *Croton toliarensis* B.W.van Ee & Kainul.

Croton leandrii var. pubescens Radcl.-Sm., Gen. Croton Madag. Comoro 188. 2016, nom. inval. (Art. 40.6). Leandri (1939, pg. 58) published "Croton arenicola f. pubescens Leandri," but as seen in the preceding paragraph, it was not validly published. Radcliffe-Smith (2016, pg. 188) later attempted to transfer it as "Croton leandrii var. pubescens (Leandri) Radcl.-Sm." Given that the intended basionym is not a validly published name, Radcliffe-Smith's new combination at a different rank is also invalid. Radcliffe-Smith (2016, pg. 188) did include a short diagnosis, which would have qualified as a new name if he had designated a holotype, but since he called the *Decary 9031* (K) specimen the "lectotype," Art. 40.6 is not satisfied for valid publication of that name.

### Excluded taxa

#### Croton haumanianus

Taxon classificationPlantaeMalpighialesEuphorbiaceae

J. Léonard, Bull. Agric. Congo Belge 48: 79. 1957

##### Type.

Democratic Republic of the Congo: Yaosuka [Yangambi], 26 Sep 1947, *J. Léonard 1448* (holotype: BR [BR00000889422]; isotypes: K [K000347428], YBI [YBI159606671]).

##### Notes.

*Croton haumanianus* is known from Madagascar from a single collection, *Bosser 17019* (P [P00133154]!, TAN), from the Station d’Essais “Caroline,” Ilaka-Est [Toamasina Province, south of Vatomandry]. According to Schmelzer (2007), *C. haumanianus* is commonly used as a shade plant in coffee and cacao plantations, which is likely the context of this report from Madagascar. No additional information is known as to whether this species has persisted in Madagascar.

#### Croton macrostachyus

Taxon classificationPlantaeMalpighialesEuphorbiaceae

Hochst. ex Delile, Voy. Abyssinie 3: 158. 1848

Oxydectes macrostachya (Hochst. ex Delile) Kuntze, Revis. Gen. Pl. 2: 610. 1891. Type. Based on Croton macrostachyus Hochst. ex Delile

##### Type.

[Ethiopia] Abyssinicum: prope Djeladjeranne, 10 Aug 1840, *W.P. Schimper 1665* (syntypes: BR [BR0000008252005], K [K000347438], K [K000347439], M [M0110345], M [M0110346], MO [1905790], MPU [MPU007279], P [P00540347], P [P00540348], P [P00540349]; possible isosyntype: MPU [MPU007276]). [Ethiopia]: in regione inferiori septentrionali montis Scholoda, 20 Jun 1837, *W.P. Schimper 196* (syntypes: BR [BR0000008252012], BR [BR0000008367969], HBG [HBG516429], HBG [HBG516430], K [K000347440], MPU [MPU007275], NY [NY00688543], S [S07-16861]).

##### Notes.

Radcliffe-Smith (1996) gave the range of *C. macrostachyus* as “throughout tropical Africa from Guinée eastwards to Ethiopia and southwards to Angola and Mozambique; also in Madagascar.” However, unlike for Malawi, Mozambique, Zambia, and Zimbabwe, he did not cite any specific specimens for Madagascar. *Gentry 11408* (MO, P [P00433498]) from Ankaratra, Antananarivo Province, Madagascar, was determined by Gentry as *C. macrostachyus*, but it is actually a specimen of the native *C. goudotii* Baill.

#### Croton sonorae

Taxon classificationPlantaeMalpighialesEuphorbiaceae

Torr., Rep. U.S. Mex. Bound. 2(1): 194. 1859

Croton furcellatus Baill., Bull. Mens. Soc. Linn. Paris 2: 967. 1891. Type. Mexico. Estado Sonora: [Ravines and mesas about] Guaymas, 1887, *E.W. Palmer 180* (holotype: P [P00404489]!; isotypes: GH [GH00303156]!, K [K000476756]!, US [00851399]!, US [00851400]!).

##### Type.

Mexico. Estado Sonora: Sierra de Nayos [Nariz], Jul 1855, *A.C.V. Schott III. 17* (holotype: NY [NY00246490]!; isotype: F [F0093623F]!).

##### Notes.

Due to mislabeled herbarium specimens, Baillon (1891a) described *Croton furcellatus* as coming from Madagascar, thinking that the type specimen had been collected by Scott-Elliot in southern Madagascar. As pointed out by Humbert (1948), Perrier de la Bâthie (1948), and Arènes (1948), a shipment of plants collected in 1887 by Edward Palmer in Mexico was sent from K to P in 1890 and was mislabeled as being Scott-Elliot collections from Madagascar. The only *Croton* among these, # 180, is a collection of *C. sonorae* Torr. from Guaymas, Sonora, Mexico (Watson 1889).

#### Croton tiglium

Taxon classificationPlantaeMalpighialesEuphorbiaceae

L., Sp. Pl. 2: 1004. 1753

Kurkas tiglium (L.) Raf., Sylva Tellur.: 62. 1838. Type. Based on Croton tiglium L.
Tiglium officinale Klotzsch, Nov. Actorum Acad. Caes. Leop.-Carol. Nat. Cur. 19 (Suppl. 1): 418. 1843. Type. Based on Croton tiglium L.
Croton officinalis (Klotzsch) Alston in H. Trimen, Handb. Fl. Ceylon 64 (Suppl.): 264. 1931, nom. superfl. Type. Based on Croton tiglium L.
Oxydectes tiglium (L.) Kuntze, Revis. Gen. Pl. 2: 614. 1891. Type. Based on Croton tiglium L.

##### Type.

Sri Lanka. (lectotype, first-step designated by Chakrabarty and Balakrishnan 1997, pg. 72, second-step designated by Philcox 1997, pg. 94: Herb. Hermann 2: 6, No. 343; left-hand specimen BM-SL [BM 000621512!]).

##### Notes.

There is an early collection of this species from Madagascar, namely *Boivin s.n.* (G), from the côte orientale de Madagascar, 1846-1852, but it has not been recorded since.

#### Lobanilia luteobrunnea

Taxon classificationPlantaeMalpighialesEuphorbiaceae

(Baker) Radcl.-Sm., Kew Bull. 44: 338 (1989)

Croton luteobrunneus Baker, J. Linn. Soc., Bot. 20: 254. 1883, as ‘*luteo-brunneum*.’ Type. Madagascar. sin. loc., s.d., *R. Baron 1770* (holotype: K [K000431062]!).

##### Type.

Based on *Croton luteobrunneum* Baker
